# A supramolecular cucurbit[8]uril-based rotaxane chemosensor for the optical tryptophan detection in human serum and urine

**DOI:** 10.1038/s41467-023-36057-3

**Published:** 2023-01-31

**Authors:** Joana Krämer, Laura M. Grimm, Chunting Zhong, Michael Hirtz, Frank Biedermann

**Affiliations:** 1 Institute of Nanotechnology (INT), Karlsruhe Institute of Technology (KIT), Hermann-von-Helmholtz Platz 1, 76344 Eggenstein-Leopoldshafen, Germany; 2 Karlsruhe Nano Micro Facility (KNMFi), Karlsruhe Institute of Technology (KIT), Hermann-von-Helmholtz Platz 1, 76344 Eggenstein-Leopoldshafen, Germany

**Keywords:** Interlocked molecules, Fluorescent probes, Biosensors, Supramolecular chemistry

## Abstract

Sensing small biomolecules in biofluids remains challenging for many optical chemosensors based on supramolecular host-guest interactions due to adverse interplays with salts, proteins, and other biofluid components. Instead of following the established strategy of developing alternative synthetic binders with improved affinities and selectivity, we report a molecular engineering approach that addresses this biofluid challenge. Here we introduce a cucurbit[8]uril-based rotaxane chemosensor feasible for sensing the health-relevant biomarker tryptophan at physiologically relevant concentrations, even in protein- and lipid-containing human blood serum and urine. Moreover, this chemosensor enables emission-based high-throughput screening in a microwell plate format and can be used for label-free enzymatic reaction monitoring and chirality sensing. Printed sensor chips with surface-immobilized rotaxane-microarrays are used for fluorescence microscopy imaging of tryptophan. Our system overcomes the limitations of current supramolecular host-guest chemosensors and will foster future applications of supramolecular sensors for molecular diagnostics.

## Abstract (web summary)

Sensing small biomolecules in biofluids using host-guest chemosensors remains challenging, in part due to the impact of interfering components. Here, the authors develop a dual-macrocyclic rotaxane for tryptophan detection which can function in biofluids such as human serum and urine.

## Introduction

Sensing small biomolecules, such as amino acids and their derivatives, is important in molecular diagnostics and personalized medicine^1–5^. However, the detection and quantification of biomarkers is so far mostly limited to clinical laboratories that are equipped with technical instruments such as high-performance liquid chromatography coupled with mass spectrometry (HPLC-MS) or nuclear magnetic resonance (NMR) capacities^6–8^. Simple-to-use, fast-responding, and inexpensive chemosensors operating through molecular recognition principles in combination with easy-to-use instrumental setups are thus highly sought-after alternatives^9–13^. Optical chemosensor-based devices for routine metal cation sensing (via ionophores) and glucose monitoring (via boronic acids) have already reached clinics and personal homes^1,14–16^. These successes are the exception rather than the rule and benefit from the comparably high concentration (in the mM range) of these target analytes. Detecting biologically or medically relevant metabolites in biofluids remains challenging as reported optical chemosensors based on host–guest chemistry show deficiencies regarding affinity, selectivity, signal transduction, and stability^1,17,18^. In particular, the various bioactive small molecules present in biofluids, combined with high concentrations of interfering proteins and salts, cause a highly complex matrix environment that fundamentally differs from the typically used solvent mixtures, deionized water, or low salt buffers^1,19^. Furthermore, the absorptivity and autofluorescence of urine and blood (serum) pose additional challenges for optical-based sensing methods.

The amino acid tryptophan (Trp) is an important metabolite as it is an essential building block in protein biosynthesis, a precursor for serotonin and melatonin, and the initial molecule of the kynurenine pathway^20,21^. Moreover, many metabolites and neurotransmitters, essential for regulating inflammations, energy homeostasis, and behavior, are routed in the tryptophan metabolism^22–24^. Consequently, Trp is an analyte of high interest as well as a therapeutic target, as its concentration levels in blood and urine correlate, e.g., with cardiovascular and neurodegenerative diseases as well as the risk of sepsis progression (see Supplementary Table 1 for selected examples)^20,21,23,25^.

State-of-the-art HPLC methods used to determine Trp levels in biofluids require cumbersome treatments, e.g., deproteinization of blood serum^25^, and therefore have little prospects for future routine use in point-of-care units, ambulances, or general medical practices. The development of synthetic receptors for tryptophan based on the principle of molecular recognition is an attractive design strategy to enable its recognition and quantification in biological matrices. Apart from the amino and carboxylate units common to all amino acids, tryptophan has no chemically reactive side chain. Its selective molecular recognition by a synthetic receptor requires the installation of noncovalent binding motifs that promote cation–π or π–π stacking interactions with the indole ring of Trp and hydrophobic interactions within the cavity^26,27^. Several optical chemosensors based on supramolecular host–guest interactions with synthetic host species such as pillar[*n*]arenes, cucurbit[*n*]urils, calix[*n*]arenes, crown ethers, or cyclodextrins have been reported to bind tryptophan in solution (mostly low saline buffers), see Table 1 and Supplementary Table 2^28–39^. Some selected systems already offer sufficient selectivity for the optical detection of tryptophan in complex media^28–37^. However, these systems often lack the required sensitivity for tryptophan, as they show only weak binding affinities (log *K*_a_ < 3)^37,39^ in low salt buffers, while others appeared to be restricted to organic media or aqueous organic mixtures^28,32,33,36,38^. Furthermore, only a few reports considered (simulated or deproteinized) blood serum as medium^34,39^. To the best of our knowledge, the detection of Trp in real, untreated (i.e., non-deproteinized) blood serum has not been investigated.

**Table 1:** Comparison of representative synthetic binders for tryptophan

Synthetic binder	Optical signal	Reported medium	log *K*_a_	Concentration range	ref.
Crown ether-type receptor^a^	Absorbance	Chloroform	~4.0	µM range (~30 µM)	^35^
Pillar[5]arene-type receptor	Emission	H_2_O:DMSO (7:3), EtOH:H_2_O (98:2)	4.0, 5.7	µM range (0–160 µM), µM range (0–130 µM)	^32,33^
Binary CB8•dye complexes	Emission	H_2_O, low saline buffers	2.0–3.0 (dye: MDPP, MDAP), ~5.0 (dye: DPT)	µM range	^29–31^
Helicene-modified β-cyclodextrin	Emission, circular dichroism	Aqueous buffer, pH 7.3	~2.5	mM range (0–4.0 mM)	^37^
18-Crown-6-modified perylene bisimide	Emission	ACN:MeOH (9:1)	~3.7	mM range (0–4.5 mM)	^36^
Calix[4]arene fluoroionophore	Emission	ACN, deproteinized blood serum (serum diluted 2:1 with acid)	9.0	nM–low µM range	^34^
γ-CD-CB6-cowheeled[4]rotaxane	Phosphorescence	Saline buffer, simulated serum^b^	2.8	µM range (0–200 µM)	^39^
CB8-viologen rotaxane	Emission	Low saline buffer, ACN	4.2	µM range (50 µM)	^28^
Rotaxane **1**	Emission	Saline buffers, biofluids	4.0	Low µM range	This work

See Supplementary Fig. 19 for the corresponding chemical structures and Supplementary Table 2.

^a^TrpOMe binding was investigated.

^b^Mixture of various amino acids.

**Fig. 1:**
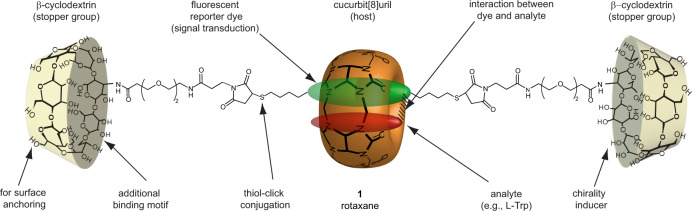
Design principle of rotaxane 1. The established binding preference and selective emission quenching of CB8•dye complexes with electron-rich aromatic analytes were exploited for the molecular engineering of a biofluid-applicable chemosensor that can detect the health-relevant amino acid Trp in human blood serum and urine. The hydrophilic and bulky β-cyclodextrin moieties act as stopper groups that prevent CB8•dye disintegration in biofluids, protect rotaxane **1** from adverse interactions with serum proteins, and enable its covalent anchoring to a surface for sensor chip preparation.

In the pursuit of a molecular recognition-based optical chemosensor for tryptophan that is both stable and operational in biofluids, we were inspired by supramolecular rotaxanes^28,40–43^, which combine various functionalities with superior tolerance to complex media. For example, the rotaxane structure not only prevents disintegration of the host-dye moiety but also restricts aggregation of the dye due to the mechanical bonding of the dye within the host^44^. Rotaxanes have received attention as molecular machines^45–47^, as supramolecular catalysts^48^, and as binders for inorganic anions^49^. In contrast to most rotaxanes, in which the axle component almost fills the host cavity completely, we developed a rotaxane design that allows for Trp binding alongside a chromophoric and fluorescent dye axle component in a hydrophobic cavity host (Fig. 1). This setup enables molecular interactions between the host and dye axle, thereby fostering affinity, selectivity, and signal transduction.

Unlike its smaller analogs, the macrocyclic cucurbit[8]uril (CB8) can simultaneously bind two aromatic compounds inside its cavity in aqueous media^50–54^. Thus, self-assembled CB8•dye complexes have been used for the complexation and detection of aryl-containing metabolites, taking advantage of optical features in ternary complex formation^17,53–55^. However, both the disintegration of CB8•dye complexes in saline biofluids due to the binding of metal cation to CB8^56^, and the competitive displacement of the dye molecule from the host cavity by hydrophobic guests (such as steroids, peptides, or biogenic amines) has typically restricted their use to deionized water or low saline buffers^17^. Moreover, the known intercalation of CB8•dye complexes into the binding pockets of serum albumin proteins^57^ is a severe drawback for the intended use in blood serum.

Herein, we demonstrate that rotaxanation of CB8 with a reporter dye and β-cyclodextrins as stopper groups provides a fluorescent macrocyclic chemosensor that is stable in complex media and can detect tryptophan in biofluids, i.e., human blood serum and urine, in the physiological concentration range. Furthermore, the utility of the rotaxane for chirality sensing, enzymatic reaction monitoring, and sensor chip preparation is showcased.

## Results

### Rotaxane design, synthesis, and characterization

Many fascinating supramolecular studies of cucurbit[*n*]uril-based rotaxanes and pseudorotaxanes have appeared since the early days of CB*n* (re)discovery^58–61^. In 2011, the first CB8-based rotaxane that can complex additional aromatic guests in aqueous and organic media was presented^28^. Its axle component, viologen, is non-emissive and provides only weak absorbance changes in the presence of Trp. This rotaxane has thus only been employed for fundamental binding studies but has no prospects for real sensing applications. First, we attempted to develop analogous chemosensing rotaxanes with a fluorescent dicationic reporter dye such as di-alkylated 2,7-diazapyrene (DAP) as the axle component. The functionalization of DAP proved to be more challenging than that of viologens, as copper-catalyzed alkyne-azide click conditions were not tolerated. In contrast, copper-free click reactions with strained alkynes yielded almost insoluble materials (see Supplementary Methods).

**Fig. 2:**
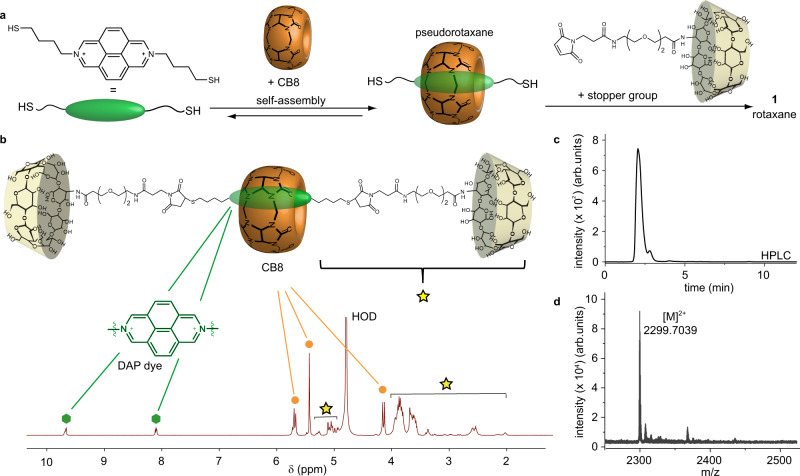
Formation and characterization of rotaxane 1. **a** Schematic representation of the synthetic route yielding rotaxane **1**. **b** ^1^H NMR of rotaxane **1** in D_2_O with chemical shift (δ) in parts per million (ppm) (green hexagons: signals of the aromatic DAP dye, orange dots: CB8 signals, yellow star: linker and β-CD signals). **c** Analytical HPLC trace of **1** (30% ACN in water, 0.1% trifluoroacetic acid). **d** ESI-MS of rotaxane **1** (1:1 ACN/H_2_O, 1% formic acid).

**Fig. 3:**
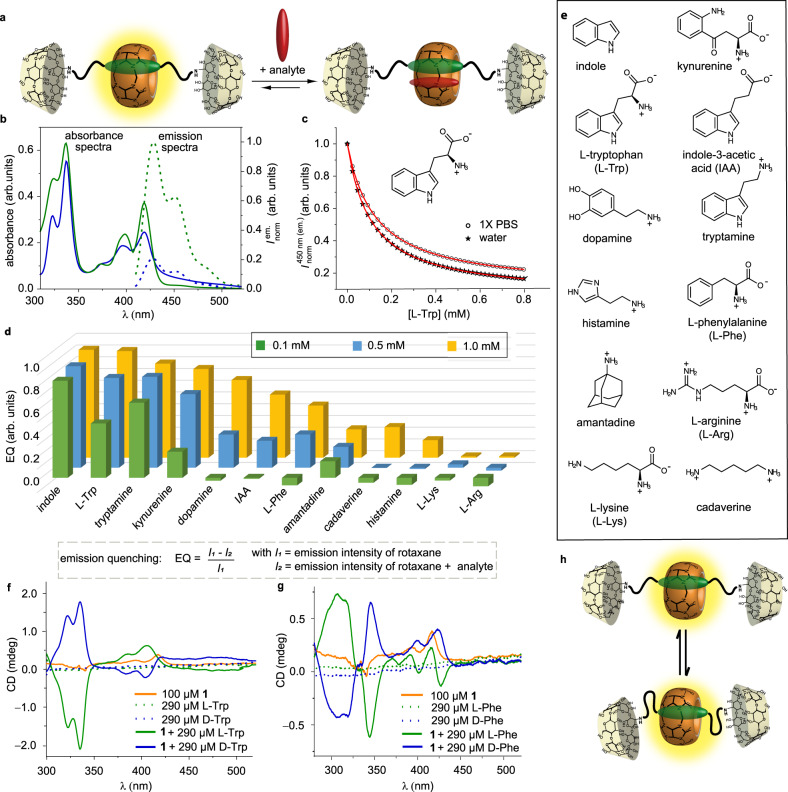
Host–guest binding with rotaxane 1. **a** Schematic representation of analyte binding by rotaxane **1**. **b** Absorbance (solid line) and normalized emission (dotted line) spectra of rotaxane **1** in the absence (green) and presence of Trp (blue) in water with *λ*_ex_ = 393 nm. **c** Binding isotherms of rotaxane **1** (*c* = 2.5 µM) and Trp in either water (stars) or 1X PBS (empty dots) and the corresponding fit (red line) of the obtained data (*λ*_ex_ = 393 nm, λ_em_ = 450 nm), measured in at least *n* = 3 independent replicates. **d** Emission quenching of rotaxane **1** (*c* = 1.0 µM) upon adding 0.1 mM (green), 0.5 mM (blue), and 1.0 mM (yellow) of bioactive analyte in 1X PBS (*λ*_ex_ = 393 nm, *λ*_em_ = 450 nm), measured in at least *n* = 4 independent replicates. **e** Chemical structures of bioactive analytes tested in this study. **f**–**g** Electronic circular dichroism (ECD) spectra of rotaxane **1** (*c* = 100 µM) in the presence of **f** l-Trp (green line) or d-Trp (blue line) and **g** l-Phe (green line) or d-Phe (blue line). For comparison, the ECD spectra of the enantiomeric amino acids are shown as dotted lines, data obtained from *n* = 1 measurement (20 accumulations). **h** Possible mechanism for chirality induction through the interaction of β-cyclodextrin stopper groups with the DAP reporter dye and the CB8 portal areas. Source data are provided as a Source Data file.

Consequently, we investigated alternative conjugation strategies that overcome the susceptibility of DAP dyes to strong bases and reducing agents. Indeed, fluorescent cucurbit[8]uril-rotaxane **1** can be prepared by a convergent synthetic route through a thiol-maleimide click coupling (Fig. 2). β-Cyclodextrin (β-CD) was identified as a suitable stopper group, ensuring the aqueous solubility of the rotaxane and offering possibilities for its further covalent (through OH bonds) and noncovalent (through its hydrophobic cavity) modification. Moreover, based on the available literature data on the interaction of cyclodextrin-drug formulations with serum albumin^62^, we expected that the installation of cyclodextrin stopper groups as part of the chemosensor would effectively prevent the undesirable encapsulation of the CB8•dye moiety into the hydrophobic binding pockets of albumin^57^, and thus would support the applicability of the resulting rotaxane in untreated blood serum. Therefore, a maleimide-functionalized polyethylene glycol (PEG) linker was coupled through its *N*-hydroxysuccinimide-functionality to 3A-amino-3A-deoxy-(2A*S*,3A*S*)-β-cyclodextrin to yield a thiol-reactive stopper group. Furthermore, the aromatic DAP core was functionalized with thio-butyl linkers. Finally, the pseudorotaxane, formed by self-assembling CB8 with the DAP axle, was covalently linked to two stopper groups via a Michael thiol-ene reaction. Rotaxane **1** was then obtained after purification by preparative HPLC and characterized by ^1^H NMR and diffusion-ordered spectroscopy (DOSY), ESI-MS, dynamic light scattering (DLS) (see Fig. 2 and Supplementary Figs. 1 and 2), as well as optical spectroscopy (Fig. 3). This design feature enables the utilization of characteristics such as chirality induction, which are typical for β-CD, see below. DLS measurements revealed self-aggregation of the chemosensing system upon heating from 20 to 50 °C, a known phenomenon for cyclodextrins in water^63^. Nevertheless, rotaxane **1** can be used for sensing applications within this temperature range as its responsiveness and binding affinities toward the desired analytes are not significantly influenced by its temperature-induced aggregation, shown in Supplementary Fig. 3 and Supplementary Table 3.

### Host–guest binding properties

First, the analyte sensing functionality of rotaxane **1** was tested by an emission-based analyte assay with indole and adamantanol (see Supplementary Fig. 4). The response of rotaxane **1** toward indole (i.e., the emission quenching of rotaxane **1** in the presence of the analyte indole) remained similarly strong in 1X phosphate-buffered saline (1X PBS) compared to that in water. Competitive binders such as adamantanol cannot displace the dye from the rotaxane-host cavity independently of their stronger binding affinity toward CB8 due to the interlocked system with the installed stoppers as anchor groups. In a second step, the host–guest binding properties of rotaxane **1** with Trp and its analogs, i.e., indole and tryptamine, were investigated in detail by emission-based titration experiments in water, 1X PBS, and human urine (Fig. 3 and Supplementary Figs. 5–8). The observed emission intensity decrease indicated analyte binding close to the DAP dye inside the CB8 cavity. In general, the simultaneous complexation of an electron-deficient aromatic dye molecule and an electron-rich aromatic molecule inside the host cavity leads to a parallel, face-to-face π–π stacking of both molecules, thereby giving rise to charge-transfer (CT) interactions^17,64^. Combined with the nonclassical hydrophobic effect, i.e., high-energy cavity water release^65^, this host-mediated π–π interaction leads to great properties in terms of binding strength and signal transduction capabilities of such CB8•dye complexes. Binding affinities (*K*_a_, see Table 2) were determined by fitting the titration curves to a 1:1 binding model (Fig. 3c). Indole, tryptamine, and Trp caused strong emission quenching of rotaxane **1** in aqueous media and human urine and showed almost identical *K*_a_ values in water and 1X PBS for each analyte. These promising results evidenced that the rotaxanation strategy endows the chemosensor with a superior salt tolerance compared to noncovalent chemosensor designs. For comparison, self-assembled macrocycle•dye chemosensors that are operational in aqueous media for Trp detection show a deteriorated performance in saline buffers and blood serum, shown in Supplementary Note 1.

**Table 2:** Binding affinities (log *K*_*a*_) of rotaxane 1 for indoyl-based analytes in aqueous media at 25 °C

Analyte/medium	Water	1X PBS	Urine (1:2 in 1X PBS)
Indole	5.42	5.48	4.44
l-Trp	3.93	3.95	3.36
		3.99^a^	
		4.10^b^	
Tryptamine	3.69	4.14	-

The estimated error of log *K*_a_ is 0.2.

^a^In the presence of 250 µM cadaverine.

^b^In the presence of 250 µM l-arginine.

Furthermore, the selectivity of rotaxane **1** was evaluated in a microwell plate format in 1X PBS by exposing rotaxane **1** to different bio-relevant analytes, such as aliphatic amino acids, amino acid derivatives, and polyamines, while recording the emission intensity. The analytes were selected as they are commonly found in human biofluids and could act as potential interferents (Fig. 3d, e). In addition, the Parkinson’s drug amantadine was chosen since it is a known strong binding guest for CB8 (log *K*_a_ = 8.9)^66^. Using Eq. (1), the obtained emission intensity of rotaxane **1** before (*I*_1_) and after analyte addition (*I*_2_) was transferred into the emission quenching efficiency of each analyte for rotaxane **1**.

1 $${{{{{\rm{Emission}}}}}}\,{{{{{\rm{quenching}}}}}}\,\left({{{{{\rm{EQ}}}}}}\right)=\frac{{I}_{1}-{I}_{2}}{{I}_{1}}=1-\frac{{I}_{2}}{{I}_{1}}$$

Aside from Trp, its structural analogs indole, tryptamine, and kynurenine caused emission quenching of rotaxane **1** in 1X PBS. However, their concentrations in blood serum and urine are significantly lower (<3 µM, see Supplementary Table 4) compared to Trp (53–77 µM in blood serum and 21–93 µM in urine). They are, therefore, not considered to be relevant interferants for the application of rotaxane **1** for Trp sensing in biofluids. In contrast, dopamine, indole-3-acetic acid (IAA), and phenylalanine (Phe) only showed significant emission quenching at non-physiologically high concentrations (>500 µM, see Supplementary Table 4 for physiological concentrations of the examined analytes). Finally, rotaxane **1** was almost unresponsive to aliphatic amino acids and polyamines as they are only weak binders. This was confirmed by an additional displacement assay with l-arginine and cadaverine (see Supplementary Fig. 8).

### Chirality sensing

Chirality plays an essential role in biology, and the occurrence of d-amino acids in living organisms is a useful indicator of various processes, including aging, diseases, or disorders^67,68^. Several molecular probes and supramolecular systems for chirality sensing of bioactive molecules have been devised to achieve higher assay throughputs with simpler handling and lower costs than contemporary chiral chromatographic methods^4,29,69–71^. Akin to self-assembled CB8•dye complexes that have been reported for chirality sensing of aromatic amino acids^29,72^, we expected that rotaxane **1** could be used for the differentiation between enantiomeric aromatic amino acids in combination with electronic circular dichroism (ECD) spectroscopy. Indeed, l-Trp can be distinguished from d-Trp and l-Phe from d-Phe upon complexation by rotaxane **1** through the emerging induced chiroptical fingerprints in the visible region of the ECD spectrum (Fig. 3f, g). Note that the slight chiroptical response of **1** and the asymmetry between the ECD spectra of **1**•l-Phe versus **1**•d-Phe can be attributed to the chirality induction caused by the chiral β-cyclodextrin stopper groups. This finding suggests that the β-cyclodextrin moieties can come close to the CB8•dye complex, as schematically depicted in Fig. 3h, and may present an additional feature by which the binding pocket of the chemosensor is protected from interferents present in biofluids. In addition, the β-cyclodextrin stopper groups offer the possibility to tune the distinct supramolecular architecture of the chemosensor.

### Tryptophan detection in biological environments

**Fig. 4:**
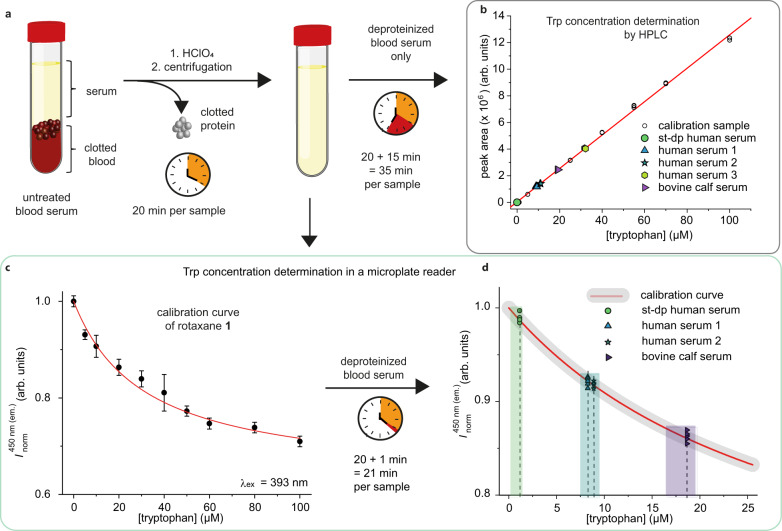
Tryptophan detection in deproteinized serum samples with rotaxane 1. **a** Schematic representation of the state-of-the-art workflow for Trp quantification in blood serum by HPLC, including the mandatory deproteinization step. **b** Trp concentration levels of deproteinized serum samples (1:1 diluted with 624 mM perchloric acid) obtained in an HPLC assay using an established calibration curve (single data points as black dots and linear fit as red line), with *n* = 3 independent measurements per serum sample and Trp concentration (*λ*_ex_ = 285 nm, *λ*_em_ = 353 nm). **c** Microplate reader-based calibration curve (single data points as black dots and nonlinear fit as red line) for Trp concentration determination with rotaxane **1** measured in *n* = 8 independent replicates; data are represented as mean values ± standard deviation (*λ*_ex_ = 393 nm, *λ*_em_ = 450 nm). **d** Correlated emission intensities of four blood serum samples given as single data points (green circle, blue and violet triangle, green hexagon, and blue star symbol), as Trp concentration ranges (colored boxes), and as mean values (dashed lines) for each serum sample, with *n* = 5 independent replicates per serum sample (*λ*_ex_ = 285 nm, *λ*_em_ = 353 nm). st-dp HS steroid-depleted human serum, BS bovine calf serum. Source data are provided as a Source Data file.

The stability in high salt-containing buffers and the good emission-response selectivity of rotaxane **1**, i.e., a signal change of at least 50% upon Trp binding, prompted us to investigate the sensing of l-Trp in blood specimens in a microwell plate format (Fig. 4). Initially, we studied the quantitative detection of Trp with rotaxane **1** in deproteinized blood serum. For this purpose, we created an emission-based calibration curve of Trp with rotaxane **1** by adding defined concentrations of Trp to a known deproteinized serum matrix and measuring the remaining emission intensity of **1**. In the next step, human and bovine blood serum samples were deproteinized according to standard clinical laboratory procedures^25^. (Note that human blood serum samples must be deproteinized in HPLC-based Trp determination methods to ensure reproducibility and avoid protein adsorption on the stationary phase.) The deproteinized serum samples were pipetted into microwell plates, and the emission intensity was recorded upon the addition of rotaxane **1** (*I*_1_). As an additional control, the autofluorescence (*I*_0_) of all serum samples was measured and used for baseline subtraction. After baseline correction and normalization, the obtained emission intensity values of each serum sample were correlated to Trp concentrations using the established calibration curve (Fig. 4c, d) and compared to the Trp levels determined by HPLC protocols^25^. The observed Trp concentration values of each unknown serum sample obtained by using rotaxane **1** were in good agreement with the HPLC-determined values (Fig. 4b) and showed a deviation of less than 15% (see Table 3 and Supplementary Fig. 9). These promising results supported us in testing quantitative Trp sensing in untreated blood serum as a next step.

**Table 3:** Trp concentrations of different blood sera were determined via HPLC (estimated error of 10% based on deviations within the repetitions) and emission-based microplate reader measurements of rotaxane **1** at 25 °C

Serum sample	[l-Trp] (µM) in serum (HPLC)	Intersect with calibration curve [l-Trp] (µM) in serum (microplate reader)	Concentration range [l-Trp] (µM) in serum (microplate reader)
Steroid-depleted human serum	<1.0 ± 0.1	2.2	0.6–2.8
Human serum 1	18.3 ± 0.2	16.6	15.1–19.1
Human serum 2	21.9 ± 0.2	18.8	16.1–19.5
Bovine calf serum	38.2 ± 0.4	37.2	32.9–39.6

**Fig. 5:**
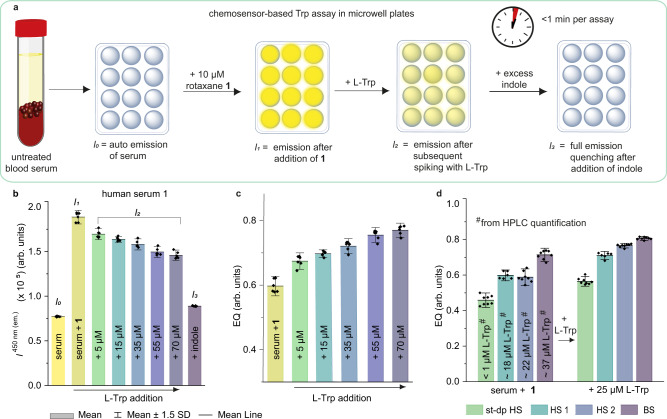
Tryptophan detection in untreated serum samples with rotaxane 1. **a** Schematic workflow for emission-based sensing of Trp with rotaxane **1** in untreated serum samples in a microwell plate format. **b** Bar graphs of the auto emission of human serum 1 (*I*_0,_ yellow), the emission intensity of rotaxane **1** in human serum 1 (*I*_1,_ light green), and the emission intensity of rotaxane **1** after additional spiking with 5–70 µM Trp (*I*_2_, green to gray), and after the addition of an excess of indole (*I*_3,_ violet). The initial Trp concentration of human serum 1 was 18 µM, and the total Trp concentration tested ranged from 18 to 88 µM throughout the assay, measured in *n* = 6 independent replicates. **c** Bar graphs of the emission quenching of rotaxane **1** in human serum 1 (light green) and after quantitative spiking with 5–70 µM Trp (green to gray), measured in *n* = 6 independent replicates and calculated according to Eq. (2). **d** Bar graphs of the emission quenching of rotaxane **1** in different serum samples and after quantitative spiking with 25 µM Trp. Initial Trp concentration values obtained by HPLC are depicted as labels for better comparison. Data points were measured in *n* = 6 independent replicates (HS1 and HS2) or *n* = 8 independent replicates (st-dp HS and BS). **b**–**d**: Bar graph heights represent the mean values of single data points (black dots), and error bars represent the standard deviation (*λ*_ex_ = 393 nm, *λ*_em_ = 450 nm). st-dp HS steroid-depleted human serum, BS bovine calf serum. Source data are provided as a Source Data file.

Therefore, the serum samples were used in their untreated form (i.e., serum samples that still contain proteins), and measurements were again conducted in a microwell plate format (see Fig. 5a for schematic procedure). The autoemission (*I*_0_), the emission upon addition of **1** (*I*_1_), and the emission reduction (*I*_2_) upon subsequent spiking with quantitative amounts of Trp in a range of 5–70 µM of Trp (Fig. 5b–d) were recorded. Finally, an indole excess was added to fully saturate the binding pocket and quench the chemosensor emission to its maximum (*I*_3_). The obtained raw emission intensities for the measurement of human serum sample 1 are shown in Fig. 5b. The recorded signal changes were converted into emission quenching (EQ) values between 0 and 1, to compare the serum samples to each other. Thus, the emission intensities (*I*_n_ with *n* = 1–2) were first corrected for the autoemission of the serum. Then, the EQ for each serum sample and the spiked serum samples (see Fig. 5c, d and Supplementary Fig. 10), was calculated according to Eq. (2) using the complete emission quenching of rotaxane **1** with indole (*I*_3_) and the emission intensity of rotaxane **1** in water (*I*_ref_) as references.

2 $${{{{{\rm{EQ}}}}}}\left({{{{\rm{serum}}}}}\right)=\frac{{({{I}_{n}-{I}_{0})}}-{I}_{{{{{\rm{ref}}}}}}}{{I}_{3}-{I}_{{{{{\rm{ref}}}}}}}$$

Pleasingly, the quenching efficiency trends obtained from our rotaxane-based optical assay not only correspond to the increasing Trp concentration upon quantitative spiking of the serum sample but also correlate with the HPLC-derived Trp levels. Moreover, Trp concentrations within the typical concentration ranges of healthy humans (~50–70 µM)^73^ and diseased patients (~20–45 µM, see Supplementary Table 1) are clearly distinguishable from each other. In addition, we tested the quantitative spiking of urine samples with Trp analogously to the above-described serum samples and obtained promising quenching efficiency trends (see Supplementary Figs. 11–12). Compared to HPLC routines, our chemosensor assay is faster (<1 min per sample) and enables the screening of many different untreated blood samples, further simplifying and shortening the assay protocol.

Moreover, rotaxane **1** was successfully used for label-free enzymatic reaction monitoring in real time, as demonstrated for the pepsin-catalyzed hydrolysis of the Trp-rich protein bovine serum albumin (BSA) in a biological saline buffer (Supplementary Fig. 13). For comparison, existing technologies offer only discontinuous data points and require time-consuming sample pre- and post-treatment steps. Besides, our results also demonstrate that supramolecular tandem assays^29,38,74^, previously mainly applied in low saline buffers, are extendable to real biofluids and complex media.

### Rotaxane microarrays for analyte detection

**Fig. 6:**
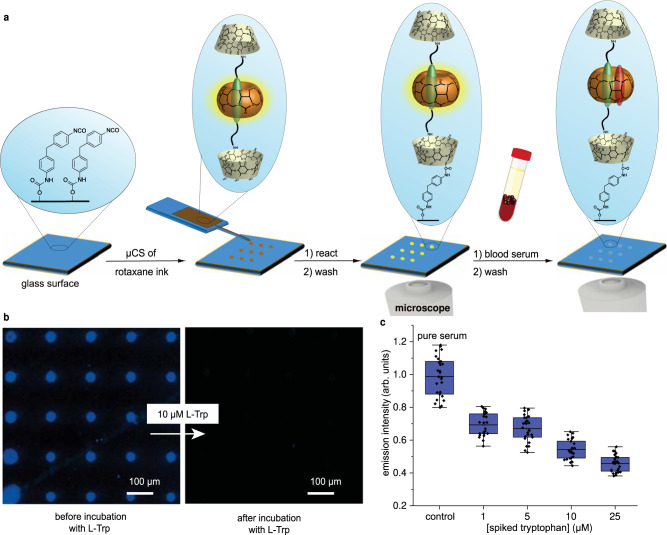
Immobilization of rotaxane 1 on glass surfaces by µCS. **a** Schematic representation of microarray printing of rotaxane **1** via microchannel cantilever spotting (µCS) on an isocyanate-covered surface and analyte detection by microarrays. **b** Fluorescence microscopy images (DAPI filter, *λ*_ex_ = 395 nm, 10 seconds exposure time) of a microarray before and after incubation with 10 μM Trp in 1X PBS showed a strong emission decrease, measured in *n* = 3 independent replicates. **c** Box plot of the emission intensity for a sensor chip prepared by µCS of **1** after incubation with different l-Trp concentrations in serum and with pure serum with an initial L-Trp concentration less than 1 µM as control. Box plots indicate the mean (middle line), 25^th^, 75^th^ percentile (blue box), and the standard deviation of all data points (single point, whiskers, with a coefficient of 1.5), *n* = 1 measurement per l-Trp concentration. Source data are provided as a Source Data file. Panel **a** adapted with permission from ref. ^80^. Copyright 2021 American Chemical Society.

The inherent functional groups of the designed rotaxane provide a synthetic handle for immobilization on functionalized glass surfaces^75^. Here, scanning probe lithography methods offer a flexible route to create micro- and even nanoscaled surface patterns of immobilized functional molecules^76^. To create microarrays of rotaxane **1**, we chose microchannel cantilever spotting (μCS), which allows for the deposition of femtoliter-sized droplets on surfaces that can act as vessels for coupling reactions^77,78^. An isocyanate-functionalized glass substrate was prepared and reacted with the OH-groups of the β-cyclodextrin stoppers (Fig. 6 and Supplementary Note 2). As an initial test of analyte detection, the microarrays were probed with indole and memantine in HEPES buffer (Supplementary Fig. 14) and compared to the binary CB8•MDAP chemosensor. Importantly, it was found that the rotaxane-functionalized microarrays are more selective than printed microarrays with the binary CB8•MDAP chemosensor complex. While both allow for the detection of indole, the hydrophobic and strongly CB8-binding guest memantine does not alter the emission of the rotaxane **1** microarray. Due to its size, memantine cannot bind next to the rotaxanated reporter dye. Moreover, it cannot displace the reporter dye due to its mechanical lock. In contrast, the CB8•MDAP sensor chips respond to the presence of memantine through dye (MDAP) displacement and thus share the undesirable cross-reactivity to other metabolites with similar bimolecular CB*n*•dye reporter pairs^1,17,79^.

Next, the sensitivity of the microarrays for tryptophan was explored. The microstructured and washable sensor chips allow for Trp detection down to submicromolar concentrations, as shown by fluorescence microscopy images and emission intensity quenching displayed in Fig. 6b (see also Supplementary Fig. 15). The remarkable sensitivity increase of rotaxane **1** by surface immobilization corresponds to our recent findings for indicator displacement chemosensors^80^. Finally, the applicability of the microarrays for Trp detection in untreated human blood serum was tested within two experimental design settings. Steroid-depleted human serum with an initial Trp concentration of less than 1 µM was spiked with controlled amounts of Trp to obtain serum samples with different Trp concentrations (see Fig. 6c and Supplementary Fig. 16). In a reverse approach, human serum (containing 58 µM of Trp determined by HPLC), as well as the same serum in different dilutions with 1X PBS, was incubated on the sensor chips (Supplementary Fig. 17). To further probe the biofluid applicability of the sensor chips with the immobilized rotaxane **1**, sensor microarrays were incubated with pure human urine (containing 60 µM Trp, determined by HPLC) and its dilutions with 1X PBS. In all cases, a dilution-dependent decrease in the fluorescence emission was observed (Supplementary Fig. 18). Overall, the results demonstrate that the microarrays can effectively detect Trp, even in complex media such as human serum or urine. The here shown working range of the sensor chips with the immobilized rotaxane **1** lies between 0 and 10 µM, which is significantly lower than that in solution-based assays. Biofluid samples of higher Trp concentrations can be diluted prior to measurement. Encapsulating such sensor chips into microfluidic channels can provide a convenient and low-volume sensing platform^80^. The possibility of immobilizing the Trp-binding rotaxane on surfaces opens up its potential for future integration into lab-on-a-chip designs with plasmonic biosensors^81^.

## Discussion

Due to its ubiquity in many biological processes and the diseases associated with those^22,23^, the amino acid tryptophan is an attractive target for the design of synthetic receptors. By comparing the binding parameters of Trp for synthetic receptors with the expected diagnostic needs, i.e., typical µM Trp concentration levels found in biofluids, we envisioned that self-assembled CB8•dye complexes provide an appropriate starting point for the development of a biofluid-compatible Trp-chemosensor. Therefore, we focused on rendering CB8•dye complexes applicable to biofluids, i.e., by pursuing a molecular engineering approach that preserved important advantages of those complexes, such as the nonclassical hydrophobic effect^65^ as driving force, the close proximity of dye and analyte in the CB8 cavity concomitant with a fast analyte response time, as well as the utility for label-free reaction monitoring and potential options to induce even more characteristics such as chirality induction by choosing suitable stopper groups. Indeed, the chosen rotaxane architecture enabled sensing of physiologically relevant Trp concentration levels in biofluids such as untreated blood serum and urine. By immobilizing the designed chemosensor, we were able to unlock low µM concentration ranges, providing additional platforms for (diluted) biofluid sensing.

The chemosensor can currently only be prepared in milligram scale by a multistep synthetic procedure and subsequent HPLC purification. Therefore, future efforts must be directed to overcome these synthetic challenges. In addition, albeit it is not specific for Trp, the chemosensor is sufficiently selective for the semiquantitative determination of medically relevant Trp concentrations. However, it remains to be seen if cross-reactivity to other indol-type analytes may become an issue if such compounds are present at higher micromolar concentrations in biofluids under specific circumstances, such as certain diseases. Therefore, additional concepts are needed when targeting biomarkers accompanied by more highly concentrated structurally similar interferents. For instance, additional recognition motifs can be installed on the dye and stopper component to enhance the chemosensor’s selectivity. Moreover, the encapsulation of the rotaxane in liposomes or polymersomes^82,83^ will enhance the binding affinity and selectivity and, at the same time, reduce the remaining impact of interferents present in biofluids. Finally, the immobilization of the rotaxane in polymeric matrices or the development of washable hydrogels should be explored in the future to improve its sensitivity by removing signal artifacts from the autoemission of biofluids.

Overall, the rotaxane design is a promising approach to overcoming the challenges of sensing in biofluids, and we hope that our model system will inspire the development of new classes of chemosensors.

## Methods

### Ethical Statement

Five urine samples, exclusively used as matrices for Trp spiking, were collected from voluntary healthy donors after detailed information about the study was given. Informed consent^84^ was obtained from all participants. No data on sex, gender, or age was collected since it was irrelevant to this study. The sample collection and analysis were performed in blinded experiments (three samples were randomly picked from five samples), and urine samples were used within one day after excretion. No health-relevant information about the spiked and non-spiked urine was gathered. All procedures performed in this study were in accordance with the formal statement of ethical principles published by the World Medical Association in the Declaration of Helsinki^85^ in 1964 and its later amendments or comparable ethical standards. The guidelines of the KIT Ethics Commission were followed.

### Materials

All chemicals and analytes were purchased from Merck, TCI, Sigma Aldrich, Acros Organics, and Alfa Aesar with at least the quality label ‘for synthesis’ and used without further purification. Dry solvents were stored over molecular sieves (3 Å or 4 Å) to ensure their aridity over long periods. Deuterated solvents were purchased from VWR Chemicals and Acros Organics. Buffer solutions were prepared following standard protocols. For 1X PBS (pH 7.4), buffer tablets from Cruz Chem were dissolved in 500 mL ultrapure water, and no further pH adjustment was carried out. Blood serum samples were purchased from Merck, Biowest, Seqens, and Cytiva. Pepsin (from porcine gastric mucosa powder) was purchased from Sigma Aldrich. Bovine serum albumin (BSA) was purchased from BioWest. Ultrapure deionized water was obtained from a Sartorius Arium® Pro Di water purification system with ASTM Type 1 water quality. All chemosensor and analyte stock solutions were prepared in ultrapure water or 1X PBS and stored at +4 °C.

Concentrations of stock solutions were determined by UV-Vis titration measurements based on the extinction coefficients of the analytes (dopamine:^86^ ε_280nm_ = 2670 M^−1^ cm^−1^; IAA:^87^ ε_280nm_ = 6000 M^−1^ cm^−1^; indole:^88^ ε_278nm_ = 5670 M^−1^ cm^−1^; MDAP: ε_393nm_ = 7800 M^−1^ cm^−1^; Phe:^89^ ε_257nm_ = 195 M^−1^ cm^−1^; tryptamine:^90^ ε_279nm_ = 5660 M^−1^ cm^−1^; Trp:^91^ ε_279nm_ = 5590 M^−1^ cm^−1^). All other analytes were precisely weighed in, dissolved, and diluted to the desired concentration accordingly.

### NMR spectroscopy

At room temperature, NMR spectra were recorded on a Bruker Avance 500 (^1^H NMR: 500 MHz; ^13^C NMR: 126 MHz). The chemical shift δ is expressed in parts per million (ppm), whereas the residual signal of the solvent was used as a secondary reference.^1^H NMR spectra were analyzed using MNova 14.2, and NMR assignment was done utilizing two-dimensional NMR spectra (COSY, HMBC, and HSQC), ^13^C NMR spectra were ^1^H-decoupled, and characterization of the ^13^C NMR spectra ensued through the DEPT technique. NMR spectra are shown in Supplementary Figs. 24–48 alongside the ESI-MS data.

### Diffusion-ordered spectroscopy (DOSY)

DOSY data were obtained on a Bruker AM 400 (400 MHz) at 298 K using a LED-bipolar gradient paired with 2 spoil gradients (ledbpgp2s), with 16 incremental steps (with 16 scans each) in the gradient strength, ramped from 2 to 98% of the maximum gradient strength. The gradient pulse length δ/2 (p30) and diffusion delay $\triangle$ (d20) were specifically optimized and set to 1.8 ms (δ = 3.6 ms) and $\triangle$ = 79.9 ms, respectively. Chemical shifts δ are expressed in parts per million (ppm) and calibrated to D_2_O as an internal standard. The spectra were calibrated and phased using TopSpin 3.1. The pseudo-2D DOSY plots were computed using Bruker Dynamic Center fitting the intensity decay.

### Dynamic light scattering (DLS)

DLS measurements were performed on a Malvern ZetaSizer Nano ZS using disposable acryl cuvettes. The chemosensor solution (150 µM in ultrapure water) was heated from 20 to 50 °C in 5 °C temperature steps using the automated heating cycle provided by the software. The intensity of the scattered light was measured at a fixed angle (173°). The wavelength of the laser light used for the light scattering experiments was set to 633 nm. Data analysis was performed according to standard procedures using the Nanosoftware V3.30. The values of viscosity and the refractive index were adapted from the provided software and plotted in Origin 9.8.

### Electrospray ionization mass spectrometry (ESI-MS)

ESI-MS experiments were carried out on a Bruker micrOTOF-Q (208–320 Vac, 50/60 Hz, 1800 VA) mass spectrometer equipped with an On-line NanoElectrospray ion source. The spectra were analyzed in MNova 14.2 and were interpreted by molecular peaks [M]^n+^ or peaks of protonated molecules [M + H]^n+^ and are shown with their mass-to-charge ratio (m/z). The solvents used were H_2_O, MeOH, and H_2_O:1% formic acid.

### Electronic circular dichroism (ECD) spectroscopy

ECD spectra were recorded on a Jasco J-1500 CD spectrometer equipped with a Peltier-thermostated cell holder and an automatic stirring unit using Jasco Spectra Manager Vers. 2. The ECD spectra reported were baseline corrected for water. All spectra were recorded at 25 °C.

### High-performance liquid chromatography (HPLC)

Analytical HPLC experiments were carried out on an LC-2000Plus HPLC system from Jasco equipped with a UV-2075 UV-Vis detector, an FP-920 fluorescence detector, and a Kromasil 100 C18 5 μm LC column (250 × 4.6 mm, Agela) at a flow rate of 1.0 mL/min. Preparative HPLC experiments were performed on the same system but equipped with a Kromasil 100 C18 5 μm LC precolumn (50 × 20 mm, Agela) and a Kromasil 100 C18 5 μm LC preparative column (250 × 50 mm, Agela) at a flow rate of 10 mL/min. All crude samples were dissolved in a mixture of water and ACN (v/v = 4/1) and applied as solutions. Data analysis was performed using ChromNav Vers. 2. DataAnalysis software from Jasco. Calibration curves were plotted in Origin 9.8. Note: At higher concentrations of rotaxane **1**, we noticed a small peak shoulder in the chromatogram, which we linked to the formation of aggregates. However, when the main peak fraction was collected and resubjected to HPLC analysis, a similar HPLC chromatogram was obtained, suggesting that the small peak shoulder is indeed not due to impurities.

### Optical spectroscopy

Absorbance spectra were measured on a Jasco V-730 double-beam UV-Vis spectrophotometer with an automatic stirring unit and were baseline corrected using Jasco’s Spectra Manager Software Vers. 2. Steady-state emission spectra were recorded on a Jasco FP-8300 fluorescence spectrometer equipped with a 450 W xenon arc lamp, double-grating excitation, emission monochromators, and a water-thermostated cell holder (STR-812) using Jasco’s Spectra Manager Software Vers. 2. Fluorescence-based titration experiments were performed manually or by an ATS-827 automatic titration unit, and the normalized emission intensity (*I*/*I*_max_) was fitted according to a 1:1 binding model by a least square fit to determine the binding affinities (see also Supplementary Figs. 5–8). All cuvettes were equipped with a stirrer allowing rapid mixing. Emission and excitation spectra were corrected for source intensity (lamp and grating).

### Monitoring of label-free enzymatic reactions

The enzymatic digestion of the protein bovine serum albumin (BSA) by pepsin was monitored by intensity changes of rotaxane **1** emission in real-time. Stock solutions of BSA and rotaxane **1**, in 1X PBS at pH 2.0, were mixed to reach a final concentration of 100 µg/mL BSA and 4.5 µM of rotaxane **1**. After equilibration (~100 s), the emission intensity was monitored over time (*λ*_ex_ = 393 nm, *λ*_em_ = 450 nm). Different volumes of a pepsin stock solution (in 1X PBS, pH 2.0) were added to reach pepsin concentrations in a range of 0–70 µg/mL. The time-resolved fluorescence traces were monitored for 20 min. As control experiments, a time course of a BSA-rotaxane mixture without pepsin addition was recorded, and a time course of the pepsin addition to rotaxane **1** without BSA was recorded, see also Supplementary Fig. 13.

### Microwell plate-based experiments

For all microwell plate-based assays, multimode microplate readers from Perkin Elmer (EnSight^TM^) and BMG Labtech (CLARIOstar® Plus), running Kaleido 3.0 and MARS 4.01 R2 as software, were used with black opaque OptiPlate^TM^-96 polystyrene microwell plates from Perkin Elmer. The instruments were equipped with monochromatic fluorescence intensity detection (top- and bottom-reading) and filter- and monochromator-based absorbance detection and temperature control. The fluorescence emission measurements were performed as full spectra or single wavelength measurements (*λ*_ex_ = 393 nm, *λ*_em_ = 450 nm) at 25 °C or in a range of 25–40 °C for temperature-dependent measurements. Data analysis for all microwell plate-based experiments was performed in Excel (Version 2209) and Origin 9.8.

#### Selectivity experiments

The emission response of rotaxane **1** upon adding aliquots of bioorganic analytes was determined in a microplate reader format at *λ*_em_ = 450 nm (*λ*_ex_ = 393 nm). Specifically, microwell plates were equipped with 100 µl of 3.0 µM rotaxane **1** in 1X PBS each. Subsequently, the analyte stock solutions were added to the wells and diluted with 1X PBS to reach a final volume of 300 µL to give a concentration of 1.0 µM for rotaxane **1**, and 100 µM, 500 µM, and 1.0 mM for the tested analyte. 1X PBS was used for blank correction, and the emission intensity of a 1.0 µM solution of rotaxane **1** was used as reference. All measured emission intensities were averaged over the repetitions performed (*n* ≥ 4) and blank corrected. The emission quenching values were then calculated according to Eq. (1) with *I*_1_ and *I*_2_ as emission intensity of rotaxane **1** (*c* = 1.0 µM) in the absence and presence of the analyte, respectively.

#### Influence of aliphatic analytes on Trp binding

The impact of nonbinding or weakly binding analytes such as l-arginine or cadaverine on the binding interaction of rotaxane **1** with Trp was examined by performing guest displacement measurements^92^. Therefore, 2 µM of rotaxane **1** was premixed with 250 µM of the potential interferent in 1X PBS in a volume of 150 µL per well, and the emission intensity (*λ*_ex_ = 393 nm, *λ*_em_ = 450 nm) was monitored before and after the addition of 0–581 µM Trp. The determined binding isotherms are shown in Supplementary Fig. 8 and Table 3.

#### Rotaxane assay for quantitative l-Trp detection in deproteinized blood serum samples

The quantitative l-Trp determination of four different deproteinized blood serum samples was carried out in a microplate reader format, and the serum samples were deproteinized according to standard methods. A calibration curve was obtained by measuring the quenching intensity of the stepwise addition of l-Trp aliquots (0–100 µM) to 10 µM of rotaxane **1** in a deproteinized serum sample, which initially contained less than 1 µM of l-Trp (st-dp human serum sample, see HPLC quantification). The measurements were done in 12 replicates in total, of which four replicates for each addition were not included in the average calculation for the calibration curve (i.e., analysis with *n* = 8 independent replicates). Five replicates of each deproteinized serum sample were measured before (auto emission of serum) and after mixing with 10 µM of rotaxane **1**. The recorded emission intensities were corrected for the auto emission of serum and the dilution factor. The l-Trp concentrations of the serum samples were determined by comparison of the emission quenching ratio to that of an independently obtained calibration curve, see Fig. 4.

#### Rotaxane assay for quantitative l-Trp determination in untreated blood serum samples

The quantitative emission quenching (EQ) of rotaxane **1** in the presence of two different blood serum samples was determined by using rotaxane **1** at a concentration of 10 µM in a microplate reader format at 450 nm (*λ*_ex_ = 393 nm). Blood serum samples were used directly without deproteinization (further called ‘untreated’). For each serum sample, at least five repetitions were conducted. Initially, for each serum sample, the wells of a 96-well plate were equipped with 150 µL of serum solution per well to obtain the autofluorescence intensity (*I*_0_). Then, 1.95 µL of a 780 µM rotaxane **1** stock solution was added to every well to reach a concentration of 10 µM of rotaxane **1**. After mixing, the emission intensity was obtained (*I*_*1*_). Subsequently, each well of the serum-chemosensor samples was spiked eight times with a l-Trp stock solution to reach l-Trp concentrations from 5 to 70 µM. After mixing, the emission intensity was obtained for each addition for every serum sample (*I*_*2*_). Finally, the total emission quenching of rotaxane **1** was reached by adding an excess of indole (300 µM) to each well (*I*_*3*_). The spiking of four different serum samples with 25 µM Trp was done similarly (see also Supplementary Fig. 10).

#### Rotaxane assay for quantitative l-Trp determination in urine samples

The emission quenching (EQ) of rotaxane **1** in the presence of two out of three different urine samples was determined by utilizing rotaxane **1** at a concentration of 10 µM in a microplate reader format at 450 nm (*λ*_ex_ = 393 nm). Urine samples were adjusted to pH 2.0 by the addition of 2 M hydrochloric acid and used as such for microplate reader measurements. Six repetitions were conducted for each urine sample, of which two values for each sample were excluded from the analysis due to errors in sample preparation. The microplate reader measurements and the data analysis were performed analogously to the measurements in serum. The bar graphs of the emission intensity decrease and the emission quenching are shown in Supplementary Fig. 12.

### HPLC-based quantification of l-Trp in serum and urine samples

Following literature procedures^25,93^, the concentration of l-tryptophan in different serum samples and urine samples was quantified using standard HPLC methods.

#### l-Trp in serum samples

A calibration curve with eight different l-Trp concentrations (*c*_final_ = 1, 5, 10, 25, 40, 55, 70, and 100 µM) was obtained with a standard stock solution of 4.9 mM of l-tryptophan in 312 mM perchloric acid. Each concentration of l-tryptophan was determined in triplicates using a mobile phase of 10% ACN in water with a flow rate of 1.0 mL/min and an injection volume of 20 µL. The calibration curve was obtained by plotting the integrated peak area of the fluorescence signal (*λ*_ex_ = 285 nm, *λ*_em_ = 353 nm) versus the standard concentrations. The slope and regression parameters were calculated by linear fitting. Five serum samples (human blood serum, steroid-depleted (st-dp) human blood serum, and bovine calf serum) with unknown Trp concentrations were deproteinized by adding an equal volume (6 mL) of 624 mM perchloric acid solution to 6 mL of the serum followed by careful mixing. The samples were stored on ice for 10 minutes to complete the precipitation and centrifuged at 6708 × g (10,000 rpm) at +4 °C for 10 minutes. Subsequently, the supernatant was separated from the protein, and after sample filtration, 20 µL of the supernatant was injected into the HPLC system for analysis. For concentration determination of l-Trp in the sera, the integrated peak areas in the chromatograms (fluorescence recording for *λ*_ex_ = 285 nm, *λ*_em_ = 353 nm) were analyzed according to the following equation (Note: factor 2 used as samples are 1:1 diluted with perchloric acid):

3 $${{{{{\rm{Trp}}}}}}\,{{{{{\rm{in}}}}}}\,{{{{{\rm{serum}}}}}}\left({{{{{\rm{\mu M}}}}}}\right)=\frac{\left({{{{{\rm{Peak}}}}}}\,{{{{{\rm{area}}}}}}\,{{{{{\rm{of}}}}}}\,{{{{{\rm{Trp}}}}}}\,{{{{{\rm{in}}}}}}\,{{{{{\rm{the}}}}}}\,{{{{{\rm{serum}}}}}}\,{{{{{\rm{sample}}}}}}\right)}{\left({{{{{\rm{Peak}}}}}}\,{{{{{\rm{area}}}}}}\,{{{{{\rm{of}}}}}}\,{{{{{\rm{Trp}}}}}}\,{{{{{\rm{in}}}}}}\,{{{{{\rm{the}}}}}}\,{{{{{\rm{standard}}}}}}\,{{{{{\rm{solution}}}}}}\right)}{{{{{\rm{x}}}}}}2$$

Triplicate measurements were performed for each serum sample, and the integrated peak areas were averaged. The determined l-Trp concentrations of each serum are shown in Supplementary Table 5. The calculated recoveries are shown in Supplementary Table 6.

#### l-Trp in urine samples

A calibration curve with six different l-Trp concentrations (final* c* = 5, 10, 25, 40, 55, and 70 µM) was obtained with a standard stock solution of 2.5 mM of l-tryptophan in synthetic urine (also called surine). Each concentration of l-tryptophan was determined in triplicates using a gradient elution program with a mobile phase consisting of eluent A (20 mM sodium acetate, 30 mM acetic acid, and 3% MeOH) and eluent B (20 mM sodium acetate/ 20 mM acetic acid, 10% ACN, and 10% MeOH) at a flow rate of 1.0 mL/min and an injection volume of 20 µL^93^. The calibration curve was obtained by plotting the intensity height of the received fluorescence signal (*λ*_ex_ = 295 nm, *λ*_em_ = 340 nm) versus the standard concentration. The slope and regression parameters were determined by linear fitting (see also Supplementary Fig. 11). Three urine samples with unknown Trp concentrations were determined directly on the day of sampling, and the pH value of the urine samples was adjusted to pH 2 with 2 M hydrochloric acid. The urine samples were centrifuged at 6708 x g (10,000 rpm) at +4 °C for 10 min to remove suspended particles, such as proteins, from the urine. Subsequently, the supernatant was separated from the precipitate, and the urine samples were mixed with an EDTA stock solution to reach a final concentration of 5 mM EDTA in urine (117 µL of 128 mM EDTA in 2283 µL urine). Finally, 20 µL of the sample solution was injected into the HPLC system for analysis after sample filtration. The obtained peaks of each urine sample were used for the concentration determination of l-Trp by integrating the peak area of the fluorescence signal (*λ*_ex_ = 295 nm, *λ*_em_ = 340 nm) according to the following equation:

4 $${{{{{\rm{Trp}}}}}}\,{{{{{\rm{in}}}}}}\,{{{{{\rm{urine}}}}}}\left({{{{{\rm{\mu M}}}}}}\right)=\frac{\left({{{{{\rm{Peak}}}}}}\,{{{{{\rm{area}}}}}}\,{{{{{\rm{of}}}}}}\,{{{{{\rm{Trp}}}}}}\,{{{{{\rm{in}}}}}}\,{{{{{\rm{the}}}}}}\,{{{{{\rm{urine}}}}}}\,{{{{{\rm{sample}}}}}}\right)}{\left({{{{{\rm{Peak}}}}}}\,{{{{{\rm{area}}}}}}\,{{{{{\rm{of}}}}}}\,{{{{{\rm{Trp}}}}}}\,{{{{{\rm{in}}}}}}\,{{{{{\rm{the}}}}}}\,{{{{{\rm{standard}}}}}}\,{{{{{\rm{solution}}}}}}\right)}$$

For each urine sample, at least duplicate measurements were conducted, and the intensity heights of the l-Trp signal were averaged. The determined l-Trp concentrations of each urine sample are shown in Supplementary Table 7 and Supplementary Fig. 11.

### Immobilization of rotaxane 1 on glass surfaces

A suitable isocyanate for immobilization was produced by activating a precleaned SiO_2_-glass surface with oxygen plasma (10 sccm O_2_, 0.2 mbar, 100 W, 2 min, ATTO system, Diener Electronics, Germany) to obtain a hydroxylated SiO_2_-OH layer. Then, the SiO_2_-OH surface was immersed in 1 mg/mL 4,4′-diisocyanato methylendibenzol (MDI) containing 1 µL/mL dibutyltin dilaurate (DBTDL) as a catalyst in anhydrous DMSO at 80 °C for 24 h. Finally, the substrate (SiO_2_-NCO) was rinsed with acetone for 2 min and dried with N_2_. Then, 0.5 µL of rotaxane ink (3 mg/mL in DMSO containing 10% DBTDL (v/v) and 10% PEG 600 (v/v) was applied to the reservoir of a microchannel cantilever^94^ (SPT-SC10S, Bioforce Nanosciences), mounted to the lithography setup (NLP2000, Nanoink), and spotted for defined durations (∼0.5–1 s) at a controlled humidity of 40%. After printing, the substrate was heated to 80 °C for 24 h, washed with ethanol, and dried with N_2_. Data analysis was performed using NIS-Elements. For one of the control experiments (Supplementary Fig. 14), thiolated glass surfaces with propargyl CB8 (CB8-Pro) arrays were prepared^80^.

### Analyte detection with rotaxane microarrays

Analyte solutions were prepared in 1X PBS (pH 7.4), st-dp human blood serum (*c*(Trp) <1 µM), or in 10 mM HEPES buffer (pH 7.0). The rotaxane-patterned substrates were covered with 20 μL analyte solution for 5 min, washed with water, and dried with N_2_. Fluorescence imaging was performed on a Nikon Eclipse Ti2 inverted fluorescence microscope (Nikon, Germany) equipped with an Intensilight illumination, a Nikon DS Qi2 camera, and a DAPI filter set (DAPI-U HQ, Nikon, Germany) with an excitation filter of 395/25 nm and a barrier filter of 460/50 nm. All data are expressed as mean ± standard deviation of three independent measurements. All concentration-dependent rotaxane microarray printing measurements were done once per concentration or dilution.

### Statistics and reproducibility

For all measurements, no statistical methods for sample size predetermination were used. The sample size of binding affinities (with *n* = 3 independent replicates and a minimum of 30 data points) was selected after careful evaluation of former binding studies conducted in our group, which gave good *R*^2^ values for the fits of the obtained data. For the measurements using serum samples, a sample size of five samples from three different suppliers was selected to get a wide variety of serum samples from different origins. For the urine measurements, a sample size of three (two samples used for microplate reader-based measurements and one for rotaxane microarrays) was chosen to show the system’s functionality in urine as a complex biomedium. For microplate reader-based measurements, single-point measurements were conducted at a fixed wavelength with one data point for each well. The size of independent replicates is individually stated in the figure captions for all measurements performed. For the immobilized rotaxane microarrays, one printing plate included several printing spots (= arrays) for the microarray measurements, each a technical replicate. The measurements of Supplementary Figs. 14a and 15a were repeated three times as independent replicates. All concentration-dependent measurements (Supplementary Figs. 16–18) were done once per concentration or dilution. However, several concentrations/dilutions (e.g., 5) were measured within each experiment. Also, several plates (e.g., 5) were printed for the experiments, as each concentration/dilution measurement had to be performed on a new plate. Therefore, we consider these measurements as independent replicates which prove the prints’ reproducibility. For the HPLC-based quantification of serum samples and the microplate reader-based calibration curve of deproteinized serum, as well as for the microplate reader-based urine measurements, a maximum of four data points were excluded due to technical errors during sample loading (HPLC) or sample preparation in wells (calibration curve of Trp in deproteinized blood serum and urine measurements). No data were excluded from the analysis for any other experiment. Doubly blinded procedures were used for all tested urine samples. The weekly preparation of fresh stock solutions of the rotaxane and the analytes ensured reproducibility. The stock solutions were stored in the fridge and used within one week. Titrations were conducted on different days, and the solutions of the microplate reader measurements were prepared individually in each well.

### Reporting summary

Further information on research design is available in the Nature Portfolio Reporting Summary linked to this article.

## Supplementary information

Supplementary Information

Reporting Summary

## Data Availability

All raw and processed data generated in this study, including data presented in the main manuscript and the Supplementary Information file, have been deposited in the Zenodo database (10.5281/zenodo.7434298)^95^. Binding parameters and chemical structures from this study are available on suprabank.org (10.34804/supra.20220128415). Synthetic procedures and data that support the findings of this study can be found in the Supplementary Information and are available on chemotion-repository.net. The identifiers for each compound are given in the Supplementary Information in the Section Supplementary Methods. Source data for the main manuscript figures are provided with this paper as a source data file.

## Source data

Source Data

## Code availability

The algorithm used for the fitting of binding isotherms is available from Zenodo (10.5281/zenodo.7434298)^95^.
