# Supplementary material for: A supramolecular cucurbit[8]uril-based rotaxane chemosensor for the optical tryptophan detection in human serum and urine
Source: Nat Commun. 2023 Jan 31;14:518. doi: 10.1038/s41467-023-36057-3 (PMC9889744; doi:10.1038/s41467-023-36057-3)
Supplement: Supplementary file 1 — Supplementary Information [file 41467_2023_36057_MOESM1_ESM.pdf]

A supramolecular CB8-β-CD-rotaxane chemosensor for the optical detection of tryptophan in human serum and urine

Joana Krämer[a], Laura M. Grimm[a], Chunting Zhong[a,b], Michael Hirtz[a,b*], and Frank Biedermann[a*]

[a] *Institute of Nanotechnology (INT), Karlsruhe Institute of Technology (KIT), Hermann-von-Helmholtz Platz 1, 76344 Eggenstein-Leopoldshafen, Germany*

[b] *Karlsruhe Nano Micro Facility (KNMFi), Karlsruhe Institute of Technology (KIT), Hermann-von-Helmholtz Platz 1, 76344 Eggenstein-Leopoldshafen, Germany*

Table of Content

Supplementary Methods **3**

1. Synthetic procedures 3
2. Additional synthetic routes that led to poorly water-soluble rotaxanes 6

Supplementary Figures **9**

1. Characterization of rotaxane 1 9
2. Binding affinity determination in a direct binding assay (DBA) 12
3. HPLC-based quantification of L-Trp in serum samples 15
4. Rotaxane assay for L-Trp determination in non-deproteinized blood serum samples 16
5. HPLC-based quantification of L-Trp in urine samples 17
6. Rotaxane assay for L-Trp determination in urine samples 17
7. Monitoring of label-free enzymatic reactions in real time 18
8. Rotaxane microarrays for analyte detection 19
9. Representative synthetic binders for Trp 23

Supplementary Notes **24**

1. Selection of commonly used non-covalent supramolecular chemosensors in several aqueous media and biomedia 24
2. Structured immobilization by microcontact printing of rotaxane 1 28
3. NMR and ESI MS spectra 30
4. Abbreviations 39

Supplementary Tables **40**

Supplementary References **42**

Supplementary Methods

Synthetic procedures

Synthetic procedures are openly accessible on Chemotion.org. The corresponding web links are given at the end of each synthetic step.

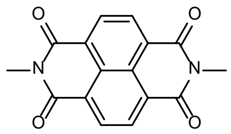

2,7-Dimethylbenzo[*lmn*][3,8]phenanthroline-1,3,6,8(2*H*,7*H*)-tetraone[1] (2): A three-neck flask equipped with a reflux condenser was filled with 120 mL aqueous methylamine (40.0 wt%, 1.39 mol, 74.6 eq) and 5.00 g of 1,4,5,8-naphthalene tetracarboxylic dianhydride (18.6 mmol, 1.00 eq) was slowly added under vigorous stirring. The orange-brown solution was refluxed for 3 h, cooled to room temperature, and stirred for 2 days. The formed precipitate was collected by filtration, washed with 100 mL methanol, and dried in vacuo. The product was isolated as a nude-colored solid with a yield of 3.09 g (10.5 mmol, 55.6%).

^{1}H NMR (500 MHz, chloroform-d_1): δ 8.78 (s, 4H, *H*-Ar), 3.61 ppm (s, 6H, CH_3); ^{13}C NMR (126 MHz, chloroform-d_1): δ 163.3 (C_q), 131.2 (C_q), 126.8 (C_q), 126.8 (CH), 27.6 ppm (CH_3).

https://dx.doi.org/10.14272/reaction/SA-FUHFF-UHFFFADPSC-FERUHHWVLZ-UHFFFADPSC-NUHFF-NUHFF-NUHFF-ZZZ.1

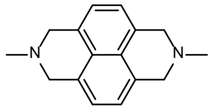

2,7-Dimethyl-1,2,3,6,7,8-hexahydrobenzo[*lmn*][3,8]phenanthroline[2] (3): A 250 mL three-neck flask was loaded with 5.30 g of anhydrous $AlCl_3$ (39.8 mmol, 2.34 eq) which was suspended in 250 mL dry THF. Under ice bath cooling, 4.50 g of $LiAlH_4$ (120 mmol, 7.05 eq) was added stepwise to the stirring suspension. After slow addition of 5.00 g of 2,7-dimethylbenzo[*lmn*][3,8]phenanthroline-1,3,6,8(2*H*,7*H*)-tetraone (**2**) (17.0 mmol, 1.00 eq), the solution turned red and was heated to reflux. The reaction mixture changed its color to green after 4 h and was cooled to room temperature. The quenching with 400 mL of ice water caused precipitation of a brown-grey solid, which was filtered off and dried in vacuo. The solid was transferred into a soxhlet extractor and extracted with 1.50 L chloroform for one day. The extract was evaporated and recrystallized from 100 mL pyridine, yielding 3.10 g of a yellow solid (12.9 mmol, 76.0%).

^{1}H NMR (500 MHz, chloroform-d_1): δ 7.12 (s, 4H, *H*-Ar), 3.88 (s, 8H, CH_2), 2.57 ppm (s, 6H, CH_3); ^{13}C NMR (126 MHz, chloroform-d_1): δ 131.8 (C_q), 127.4 (C_q), 121.7 (CH), 58.7 (CH_2), 45.6 ppm (CH_3).

https://dx.doi.org/10.14272/reaction/SA-FUHFF-UHFFFADPSC-VHNSKBKZGH-UHFFFADPSC-NUHFF-NUHFF-NUHFF-ZZZ

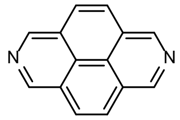

Benzo[*lmn*][3,8]phenanthroline[2] (DAP) (4): In a 250 mL flask equipped with a mechanical stirring bar, 7.70 g of selenium (97.5 mmol, 19.4 eq) and 1.20 g of 2,7-dimethyl-1,2,3,6,7,8-hexahydrobenzo[*lmn*][3,8]phenanthroline (**3**) (5.04 mmol, 1.00 eq) were stirred at 265 °C for 4 h. The black viscous mixture was heated to 300 °C for 1 h and then cooled to room temperature. Next, 100 mL of 1 M aqueous HCl was added and stirred under reflux for 30 minutes. This procedure was repeated three times in total, and after each boiling, the black solid was filtered off. The filtrates were combined, and aqueous NaOH (5 M) was added until pH 12.0 was reached. The formed yellow

precipitate was separated by filtration, dissolved in 1 M HCl, and precipitated again with 1 M NaOH. This procedure was repeated twice. Finally, the obtained precipitate was filtered off and dried in vacuo. The product was isolated as a yellow solid with a yield of 720 mg (3.52 mmol, 70.0%).

^{1}H NMR (500 MHz, chloroform-d_1): δ 9.48 (s, 4H, *H*-Ar), 8.17 ppm (s, 4H, *H*-Ar); ^{13}C NMR (126 MHz, chloroform-d_1): δ 145.5 (CH), 126.5 (C_q), 126.5 (C_q), 126.0 ppm (CH).

https://dx.doi.org/10.14272/reaction/SA-FUHFF-UHFFFADPSC-ZPIPUFJBRZ-UHFFFADPSC-NUHFF-NUHFF-NUHFF-ZZZ.1

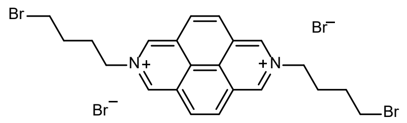

2,7-Bis(4-bromobutyl)benzo[*lmn*][3,8]phenanthroline-2,7-diium dibromide (5): A solution of 50.0 mg DAP (**4**) (245 µmol, 1.00 eq) was prepared in 10.0 mL dry DMF under nitrogen atmosphere. To the reaction solution, 2.20 mL 1,4-dibromobutane (3.96 g, 18.4 mmol, 75.0 eq) was added, and the reaction mixture was stirred at 75 °C for 48 h. After cooling, the yellow precipitate was filtered off, washed with 25 mL DMF, and dried under reduced pressure. The isolated product was obtained as a yellow solid with a yield of 71.4 mg (112 µmol, 46.0%).

^{1}H NMR (500 MHz, D_2O): δ 10.11 (s, 4H, *H*-Ar), 8.85 (s, 4H, *H*-Ar), 5.23 (t, J = 7.5 Hz, 4H, C*H₂*), 3.56 (t, J = 6.4 Hz, 4H, C*H₂*), 2.54 (quin, J = 7.5 Hz, 4H, C*H₂*), 2.04 ppm (quin, J = 7.5 Hz, 4H, C*H₂*); ^{13}C NMR (126 MHz, D_2O): δ 141.1 (CH), 130.0 (C_q), 129.9 (CH), 127.0 (C_q), 63.0 (CH_2), 33.0 (CH_2), 30.1 (CH_2), 28.5 ppm (CH_2); HRMS (ESI) (pos., H_2O) (m/z): $[M]^{2+}$ calcd. for $C_{22}H_{24}Br_2N_2^{2+}$, 238.0134; found, 238.0134.

https://dx.doi.org/10.14272/reaction/SA-FUHFF-UHFFFADPSC-LHBYXQPXZL-UHFFFADPSC-NUHFF-LUHFF-NUHFF-ZZZ

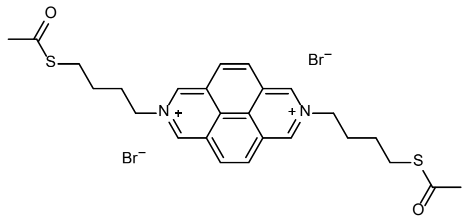

2,7-Bis(4-(acetylthio)butyl)benzo[*lmn*][3,8]phenanthroline-2,7-diium dibromide (6): In a vial, 113 mg of 2,7-bis(4-bromobutyl)-benzo[*lmn*][3,8]phenanthroline-2,7-diium dibromide (**5**) (178 µmol, 1.00 eq) was dissolved in 10.0 mL ultrapure water and 60.9 mg of potassium thioacetate (533 µmol, 3.00 eq) dissolved in 5.00 mL ultrapure water was added. The reaction solution was stirred for 2 days at room temperature under the exclusion of light. The solvent was removed under reduced pressure, and the product was obtained as a brown solid with a yield of ≥ 99% due to residual potassium thioacetate and used as such in the following synthetic step.

^{1}H NMR (500 MHz, D_2O): δ 10.10 (s, 4H, *H*-Ar), 8.85 (s, 4H, *H*-Ar), 5.21 (t, J = 7.4 Hz, 4H, C*H₂*), 2.96 (t, J = 7.4 Hz, 4H, C*H₂*), 2.35 (quin, J = 7.4 Hz, 4H, C*H₂*), 2.31 (s, 6H, C*H₃*), 1.75 ppm (quin, J = 7.4 Hz, 4H, C*H₂*); ^{13}C NMR (126 MHz, D_2O): δ 201.7 (C=O), 141.1 (CH), 130.0 (CH) 129.8 (C_q), 127.0 (C_q), 63.2 (CH_2), 30.2 (CH_2), 30.0 (CH_3), 27.8 (CH_2), 25.5 ppm (CH_2); HRMS (ESI) (pos., MeOH) (m/z): $[M]^{2+}$ calcd. for $C_{26}H_{30}N_2O_2S_2^{2+}$, 233.0869; found, 233.0867.

https://dx.doi.org/10.14272/reaction/SA-FUHFF-UHFFFADPSC-LNNFMCALYY-UHFFFADPSC-NUHFF-LUHFF-NUHFF-ZZZ

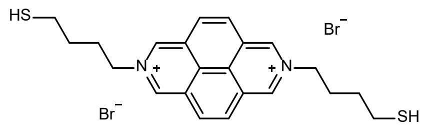

2,7-Bis(4-mercaptobutyl)benzo[*lmn*][3,8]phenanthroline-2,7-diium dibromide (7): 37.2 mg of 2,7-bis(4-(acetylthio)butyl)benzo[*lmn*][3,8]-phenanthroline-2,7-diium dibromide (**6**) (59.0 µmol, 1.00 eq) was dissolved in 5.00 mL dry MeOH and degassed with N_2 for 15 minutes. To the solution, 170 µL acetyl chloride (187 mg, 2.36 mmol, 40.0 eq) was added under a nitrogen atmosphere, and the mixture was stirred for 2.5 days at room temperature. The reaction mixture was quenched by the addition of water, causing the precipitation of a black solid. The solid was separated by filtration, and the solvent was removed from the filtrate. The product was isolated as a red-brown solid with a yield of 20.9 mg (38.4 µmol, 65.0%).

^{1}H NMR (500 MHz, D_2O): δ 10.10 (s, 4H, *H*-Ar), 8.86 (s, 4H, *H*-Ar), 5.21 (t, 4H, J = 7.4 Hz, CH_2), 2.63 (t, 4H, J = 7.0 Hz, CH_2), 2.40 (quin, 4H, J = 7.4 Hz, CH_2), 1.77 ppm (quin, 4H, J = 7.4 Hz, CH_2); ^{13}C NMR (126 MHz, D_2O): δ 141.1 (CH), 130.0 (CH), 129.8 (C_q), 127.0 (C_q), 63.3 (CH_2), 30.1 (CH_2), 29.5 (CH_2), 23.0 ppm (CH_2); HRMS (ESI) (pos., MeOH) (m/z): [M-H]$^+$ calcd. for $C_{22}H_{25}N_2S_2^+$, 381.1415; found, 381.1450.

https://dx.doi.org/10.14272/reaction/SA-FUHFF-UHFFFADPSC-DYWOOYJZBS-UHFFFADPSC-NUHFF-NUHFF-NUHFF-ZZZ

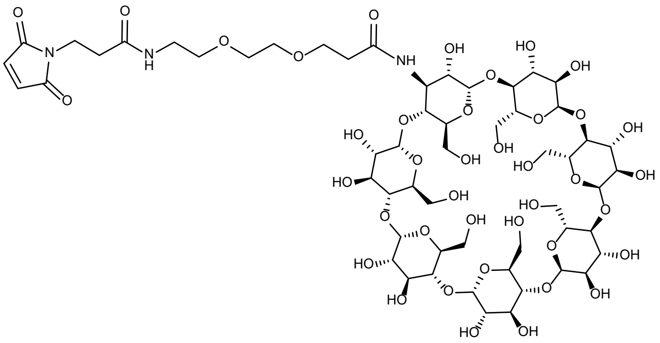

TEG-maleimide-β-CD (8):

In a vial, 57.1 mg of 3-Amino-3A-deoxy-(2A*S*,3A*S*)-β-cyclodextrin hydrate (50.0 µmol, 1.00 eq) and 23.8 mg of maleimide-PEG$_2$-NHS ester (56.0 µ mol, 1.20 eq) were combined and dried in vacuo to remove water from the solids as maleimides are sensitive to hydrolysis. The solids were dissolved in 10 mL dry DMF, and the mixture was degassed with nitrogen. After adding 100 µL of NEt_3 (73.0 mg, 721 µmol, 14.4 eq), the reaction solution was stirred overnight at room temperature. The solvent was removed under reduced pressure, and the residue was dissolved in 2.0 mL deionized water and purified by HPLC (gradient: 0-100% ACN in water, 0.1% TFA). The product was isolated as a colorless powder with a yield of 61.8 mg (42.7 µmol, 85.0%).

^{1}H NMR (500 MHz, D_2O): δ 6.88 (s, 2H, C*H*), 5.08-4.99 (m, 6H, C*H*), 4.94 (d, J = 5.8 Hz, 1H, C*H*), 4.28 (s (br), 1H, C*H*), 3.95-3.53 (m, 52H, C*H*, CH_2), 3.34-3.32 (m, 2H, CH_2), 2.60-2.51 ppm (m, 4H, C*H*, CH_2); ^{13}C NMR (126 MHz, D_2O): δ 174.3 (C_q), 172.9 (C_q), 172.1 (C_q), 134.5 (CH), 102.0 (CH), 101.9 (CH), 81.3 (CH), 81.1 (CH), 80.9 (CH), 73.2 (CH), 73.1 (CH), 72.7 (CH), 72.3 (CH), 72.0 (CH), 71.9 (CH), 71.8 (CH), 71.4 (CH), 69.5 (CH_2), 68.9 (CH_2), 66.7 (CH_2), 60.2 (CH_2), 51.0 (CH), 38.9 (CH_2), 34.7 (CH_2), 34.5 ppm (CH_2); HRMS (ESI) (pos., MeOH) (m/z): [M+H]$^+$ calcd. for $C_{56}H_{89}N_3O_{40}H^+$, 1444.5095; found, 1444.5083.

https://dx.doi.org/10.14272/reaction/SA-FUHFF-UHFFFADPSC-UFNDHZJPQR-UHFFFADPSC-NUHFF-NUOFM-NUHFF-ZZZ

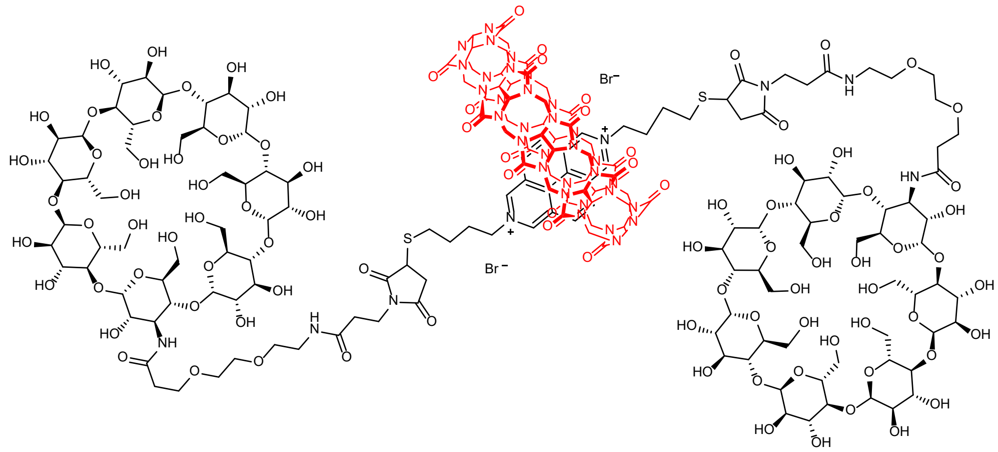

Rotaxane (1): In a vial, 50.0 mg of cucurbit[8]uril (CB8) (38.0 µmol, 1.10 eq) was dissolved in 50 mL ultrapure water and 14.5 mL of a 2.4 mM aqueous solution of 2,7-bis(4-mercaptobutyl)benzo[*lmn*][3,8]phenanthroline-2,7-diium dibromide (**7**) (18.9 mg, 35.0 µmol, 1.00 eq) were added to the solution. Before synthesis, the thiol concentration (2 eq per dye molecule) of **7** was determined through the Ellman test.[3] The mixture was diluted with 8.00 mL of sodium phosphate buffer, pH 7.7, causing a color change from yellow to bright green. After sonication for 15 minutes, 111 mg of TEG-maleimide-β-CD (**8**) (77.0 µmol, 2.20 eq) was added, and the mixture was stirred at room temperature overnight, resulting in a slow color change from green back to yellow. The solvent was removed under reduced pressure, and the residue was washed with 1:1 EtOH: water three times with subsequent removal of the formed colorless solid by centrifugation. The combined solutions were dried in vacuo, and purification was done by HPLC (mobile phase: 17% ACN in water (0.1% TFA) for 45 min, then 100% ACN for 15 min). The product was obtained as a yellow solid with a yield of 59.4 mg (12.4 µmol, 35.0%).

^{1}H NMR (500 MHz, D_2O): δ 9.67 (br, 4H, C*H*), 8.11 (br, 4H, C*H*), 5.67 (d, J = 14.9 Hz, 16H, *CH₂*), 5.44 (s, 16H, *CH₂*), 5.27-4.93 (m, 20H, C*H*, C*H₂*), 4.16 (d, J = 15.4 Hz, 16H, *CH₂*), 3.95-3.55 (m, 110H, C*H*, *CH₂*), 3.41-3.36 (m, 4H, C*H*), 3.30-2.81 (m, 2H, C*H₂*), 2.61-2.53 (m, 14H, C*H*, C*H₂*), 2.03 ppm (br, 2H, C*H*); HRMS (ESI) (pos., 1:1 ACN/H_2O, 1% formic acid) (m/z): $[M]^{2+}$calcd. for $C_{182}H_{252}N_{40}O_{96}S_2^{2+}$, 2299.2765; found, 2299.7039.

https://dx.doi.org/10.14272/reaction/SA-FUHFF-UHFFFADPSC-YRODUCXAHM-UHFFFADPSC-NUHFF-NDLGW-NUHFF-ZZZ

Additional synthetic routes that led to poorly water-soluble rotaxanes

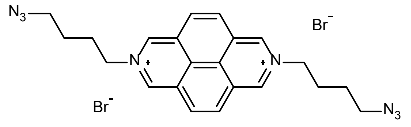

2,7-Bis(4-azidobutyl)benzo[*lmn*][3,8]phenanthroline-2,7-diium dibromide (9): A solution of 25.0 mg of 2,7-bis(4-bromobutyl)benzo[*lmn*][3,8]phenanthroline-2,7-diium dibromide (**5**) (39.4 µmol, 1.00 eq) was prepared in 2.5 mL deionized water and mixed with a solution of sodium azide (17.1 mg, 262 µmol, 6.60 eq) pre-dissolved in 2.5 mL ultrapure water. The reaction mixture was heated to

80 °C for 3 h before it was concentrated in vacuo. The product was isolated as a yellow solid with a yield of 19.1 mg (34.1 µmol, 87.0%).

^{1}H NMR (500 MHz, D_2O): δ 10.03 (s, 4H, *H*-Ar), 8.77 (s, 4H, *H*-Ar), 5.14 (t, J = 7.4 Hz, 4H, C*H₂*), 3.36 (t, J = 6.6 Hz, 4H, C*H₂*), 2.28 (quin, J = 7.4 Hz, 4H, C*H₂*), 1.68 ppm (quin, J = 7.4 Hz, 4H, C*H₂*); ^{13}C NMR (126 MHz, D_2O): δ 141.1 (CH), 130.0 (C_q), 129.8 (C_q), 63.3 (CH_2), 50.3 (CH_2), 28.8 (CH_2), 24.9 ppm (CH_2).

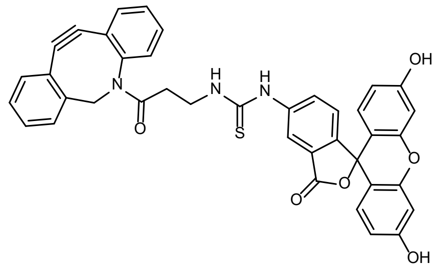

DBCO-fluorescein[4] (10): 14.8 mg of 5-isothiocyanate fluorescein (39.0 µmol, 1.10 eq) and 9.80 mg of DBCO-amine (35.0 µmol, 1.00 eq) were dissolved in a mixture of acetonitrile and carbonate buffer, pH 10.0 (v:v 3:1). The mixture was stirred for 7 days at room temperature, and the reaction progress was controlled via TLC (ethyl acetate as mobile phase, colored with Seebach solution). The reaction solvent was removed under reduced pressure. The crude product was used without further purification; no NMR spectra were recorded.

MS (ESI) (pos., MeOH) (m/z): $[M+Na]^+$ calcd. for $C_{39}H_{27}N_3O_6SNa^+$, 688.1513; found, 688.1647.

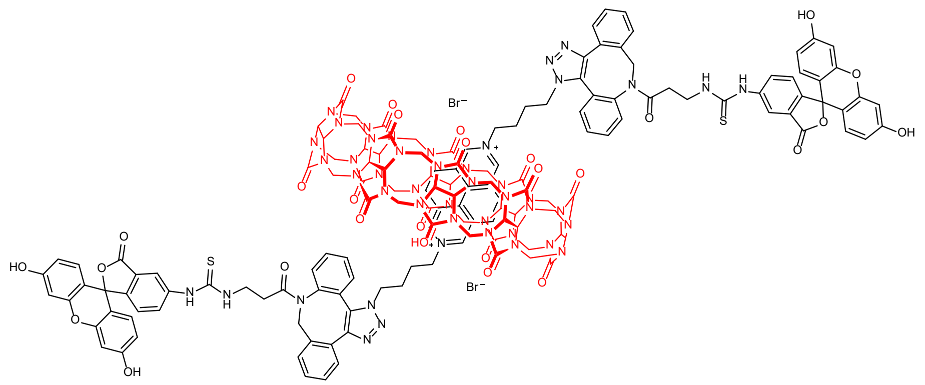

DBCO-fluorescein rotaxane (11): In a reaction flask, 3.60 mg of 2,7-bis(4-azidobutyl)benzo[*lmn*][3,8]-phenanthroline-2,7-diium dibromide (**9**) (9.00 µmol, 1.00 eq) was dissolved in ultrapure water and 14.3 mg of CB8 (10.8 µmol, 1.20 eq) was added as solid. The reaction mixture was stirred for 30 minutes at room temperature. Subsequently, 18.0 mg DBCO-fluorescein (**10**) (27.0 µmol, 3.00 eq) was added, and the reaction mixture was stirred for 4 days at room temperature. A red precipitate was formed and filtered off. The remaining reaction solution was overlaid with 7.5 mL methanol and stored in the fridge for 1 day. The formed precipitate was filtered off and combined with the previously gained red solid. NMR analysis was not possible due to the low solubility of the product.

MS (ESI) (neg., DMSO/H_2O 1:1) (m/z): $[M]^{2+}$ calcd. for $C_{148}H_{126}N_{46}O_{28}S_2^{2+}$, 1529.8493; found, 1530.4543.

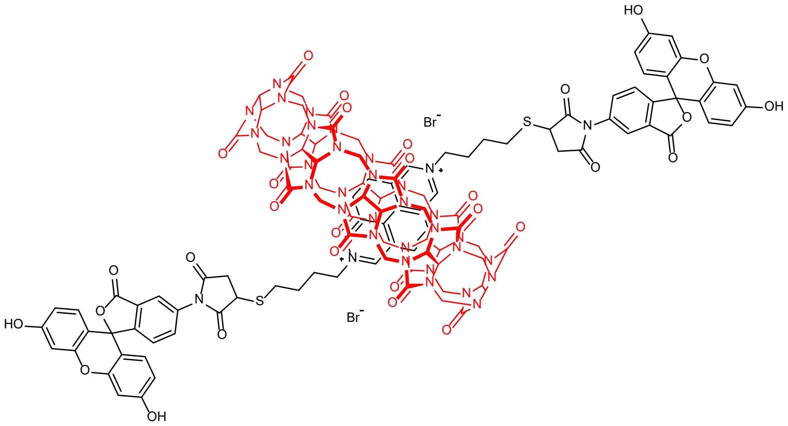

FITC-rotaxane (12): 949 µg of 2,7-bis(4-(acetylthio)butyl)benzo[*lmn*][3,8]phenanthroline-2,7-diium dibromide (**7**) (1.75 µmol, 1.00 eq) was dissolved in ultrapure water and 3.50 mg of CB8 (2.63 µmol, 1.50 eq) was added as solid. The reaction mixture was stirred at room temperature for 30 minutes. Fluorescein-5-maleimide was dissolved in dry DMSO with a final concentration of 1 mM. 3.50 mL (equal to 1.50 mg, 3.5 µmol, 2.00 eq) of this 1 mM solution were added to the aqueous reaction mixture, which was stirred for 3 days. The reaction mixture was dried in vacuo, redissolved in water, and lyophilized. Several purification attempts failed, such as normal phase column chromatography and crystallization. HPLC purification proceeded by running a gradient from 20% ACN to 60% ACN in water containing 0.1% TFA. NMR analysis was not possible due to the low solubility of the product.

HRMS (ESI) (pos., DMSO/H_2O 3:1) (m/z): $[M]^{2+}$ calcd. for $C_{118}H_{110}N_{36}O_{30}S_2$, 1282.8432; found, 1282.8538.

Supplementary Figures

1. Characterization of rotaxane 1

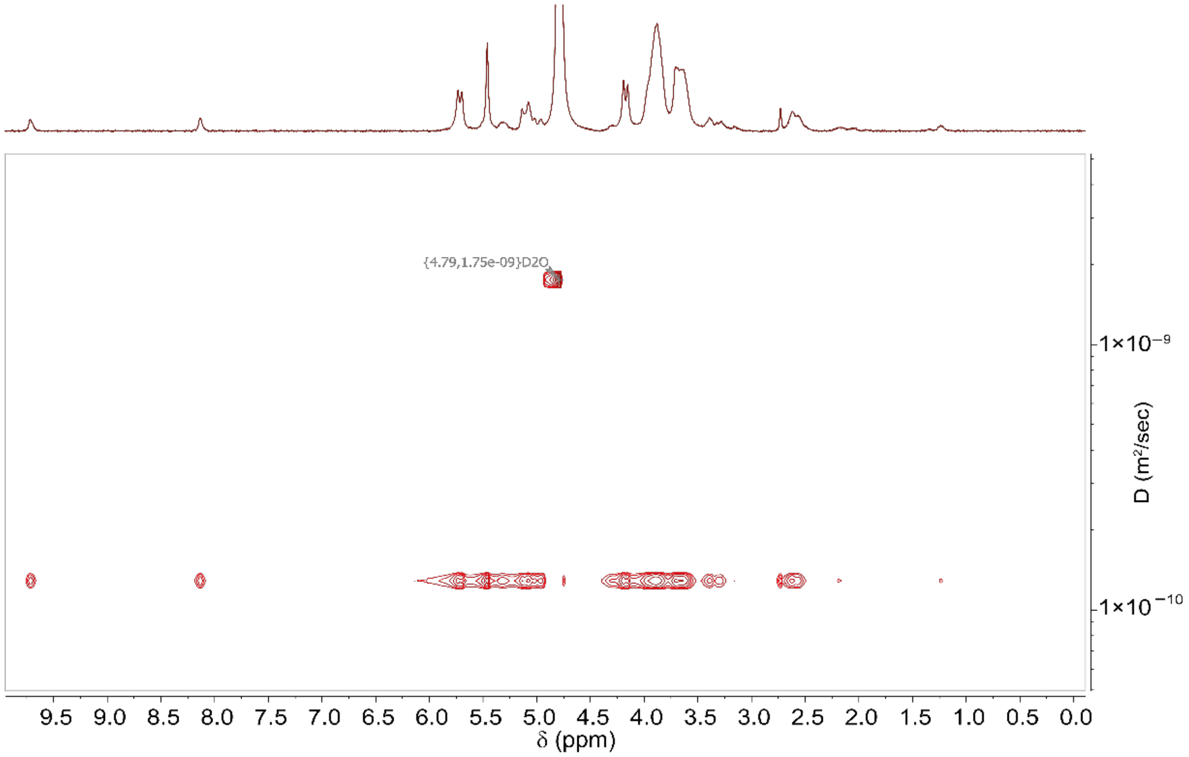

Supplementary Fig. 1. 2D DOSY spectrum of rotaxane **1** recorded in D_2O. All ^1H NMR signals show the same diffusion coefficient, which indicates that they correspond to one single molecule, *i.e.*, the desired rotaxane.

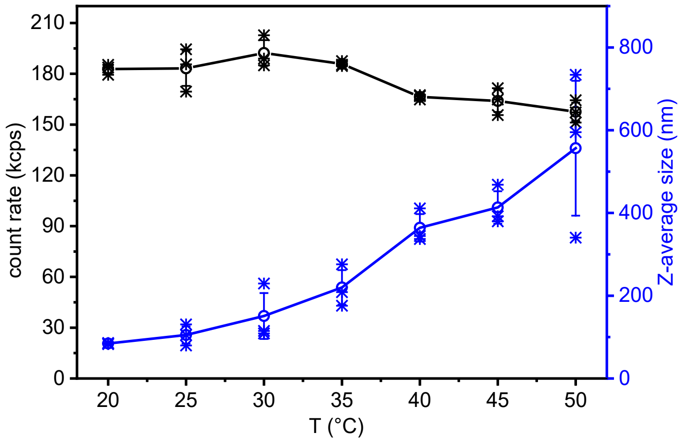

Supplementary Fig. 2. Temperature-dependent count rates (black) and Z-average size (blue) of rotaxane **1** within a temperature range of 20 to 50 °C. Single data points of independent replicates (n = 3) are shown as stars, whereas empty circles represent the mean values of the individual measurements. Error bars present the mean values +/- standard deviation. Connecting lines between the empty circles are included to guide the eye. An increase in Z-average size with rising temperature indicates the formation of large aggregates, which is a known behavior of cyclodextrins.[5] However, the aggregation does not influence the analyte sensing behavior of rotaxane **1**, see Supplementary Fig. 3

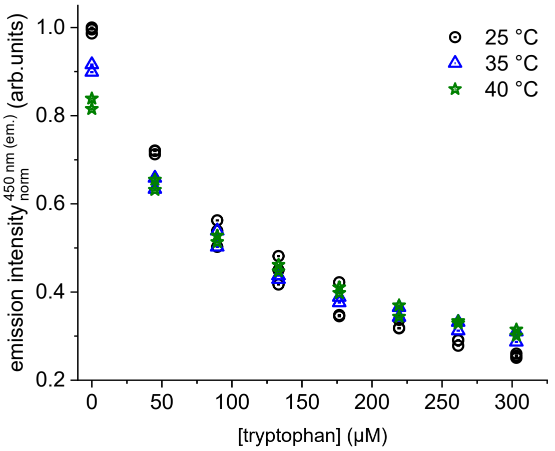

Supplementary Fig. 3. Emission-based titration measurements (λ_{ex} = 393 nm, λ_{em} = 450 nm) of Trp (c = 0 - 303 µM) to rotaxane **1** (c = 2 µM) in 1X PBS at 25 °C (black dots), 35 °C (blue triangles), and 40 °C (green stars), measured in n = 3 independent replicates. Independent replicates are shown as individual data points. The obtained binding isotherms all show a similar shape. The determined binding constants do not differ significantly between the different temperatures, indicating that the sensing ability of rotaxane **1** is not influenced by its temperature-induced aggregation. The binding affinity values are listed in Supplementary Table 3.

.

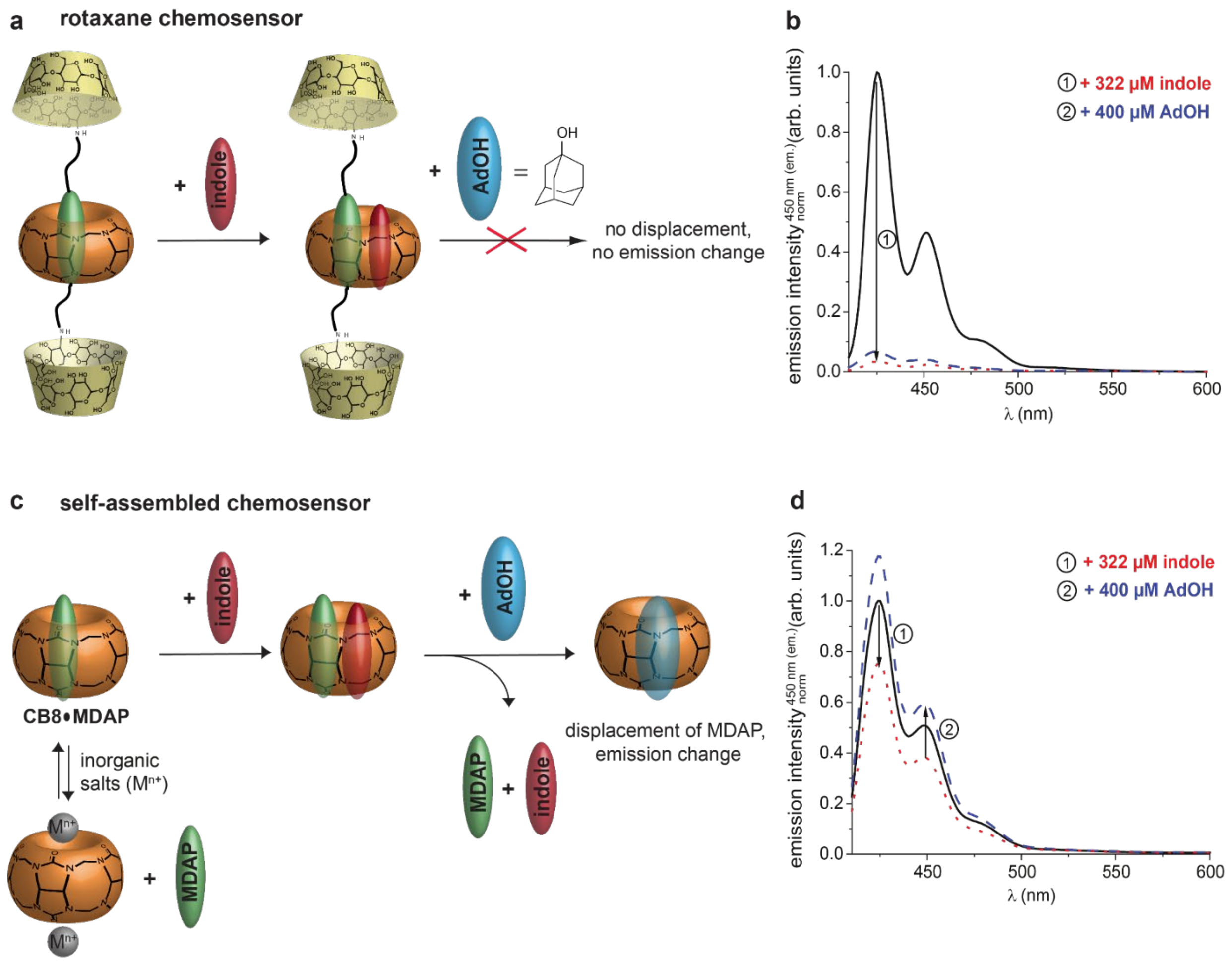

Supplementary Fig. 4. Test of the analyte-sensing functionality of rotaxane **1** as a chemosensor by an emission-based analyte assay with indole. The response of rotaxane **1** towards indole remained similarly strong in 1X PBS compared to water. In contrast, due to competing salt cations, the bimolecular chemosensor CB8•MDAP completely lost its functionality in 1X PBS. **a** Schematic representation of analyte binding of rotaxane **1** towards the electron-rich aromatic analyte indole and for the bulky (not fitting next to the dye due to its size) analyte 1-AdOH. **b** Emission quenching of rotaxane **1** (black line) was observed after the addition of indole (dotted red line) due to the dye-analyte interaction. The subsequent addition of 1-AdOH (dotted blue line) did not cause any significant signal change, since 1-AdOH is too bulky to bind next to the DAP dye and cannot displace the dye due to the interlocked system with the installed stoppers as anchor groups. **c** Schematic representation of analyte binding to a self-assembled CB8•MDAP chemosensor. Bulky and strongly binding analytes such as 1-AdOH displace the bound dye and disassemble the chemosensor. **d** Emission response of the CB8•MDAP chemosensor (black line) in the presence of indole (dotted red line) and 1-AdOH (dotted blue line). The higher emission signal compared to the starting signal after 1-AdOH addition indicated the disassembly of the CB8•MDAP complex (dye emission is slightly quenched in the host cavity). All emission spectra were recorded in 1X PBS (black) at 25 °C (λ_{ex} = 393 nm, λ_{em} = 450 nm).

2. Binding affinity determination in a direct binding assay (DBA)

$$\text{rotaxane} + \text{G} \rightleftarrows \text{rotaxane} \bullet \text{G} \tag{1}$$

$$K_\text{a}^{\text{rotaxane}\bullet\text{G}} = \frac{[\text{rotaxane} \bullet \text{G}]}{[\text{rotaxane}][\text{G}]} \tag{2}$$

$$[\text{rotaxane}]_0 = [\text{rotaxane} \bullet \text{G}] + [\text{rotaxane}] \tag{3}$$

$$[G]_0 = [\text{rotaxane} \bullet \text{G}] + [\text{G}] \tag{4}$$

$$I_c = I^0 + I^{\text{rotaxane}\bullet\text{G}} \cdot [\text{rotaxane} \bullet \text{G}] + I^{\text{G}} \cdot [\text{G}] \tag{5}$$

with K_a = binding constant, [G] = guest/analyte, I = emission intensity

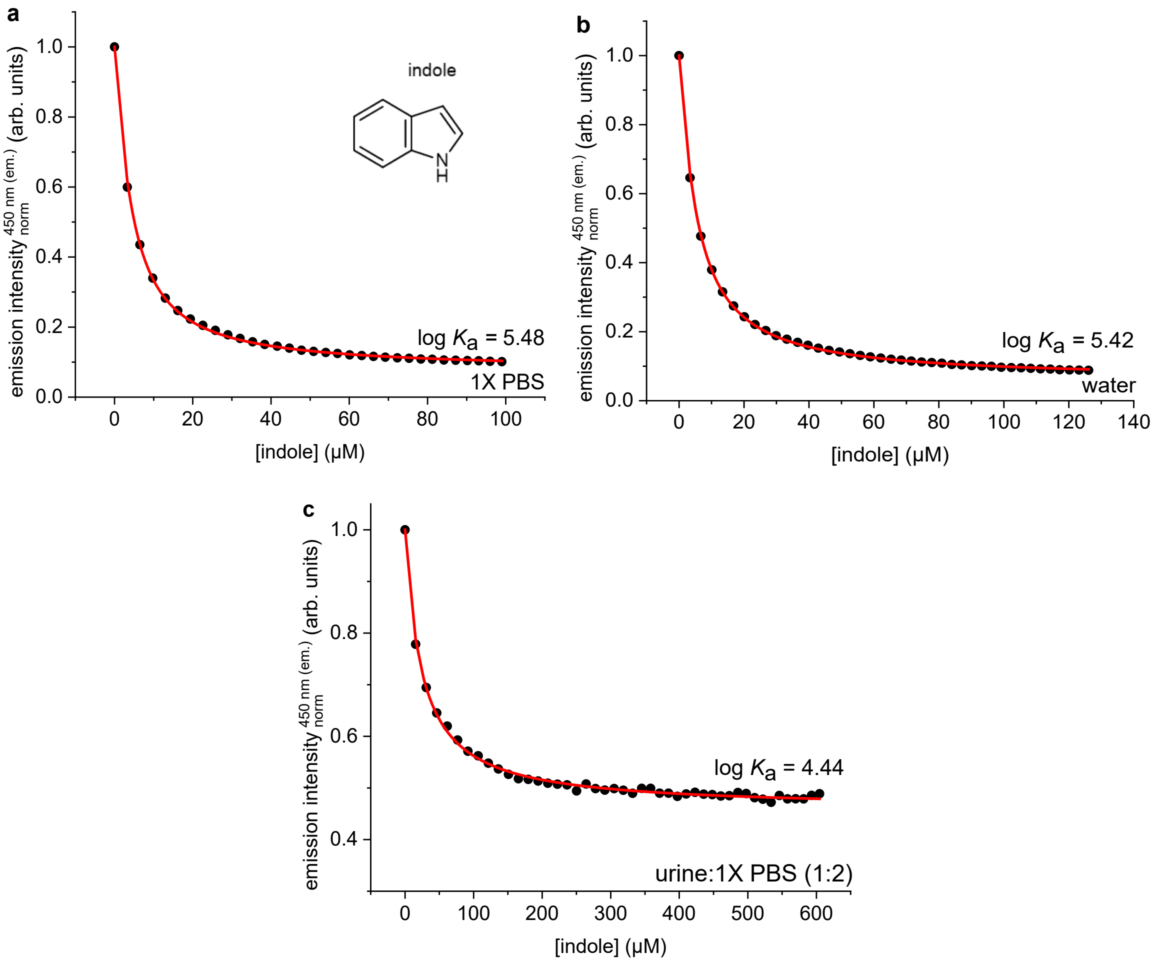

Supplementary Fig. 5. Representative DBA binding isotherms determined by emission-based titration measurements (λ_{ex} = 393 nm, λ_{em} = 450 nm): **a** indole (c = 0 - 100 µM) to rotaxane **1** (c = 2.5 µM) in 1X PBS at 25 °C, **b** indole (c = 0 - 126 µM) to rotaxane **1** (c = 2.5 µM) in water at 25 °C, and **c** indole (c = 0 - 662 µM) to rotaxane **1** (c = 1.0 µM) in urine (1:2 in 1X PBS) at 25 °C. Black circles depict the acquired data. The fit according to a 1:1 binding model is shown as red line. Log K_a was determined in at least triplicates and therefore errors in log K_a are estimated to be not larger than 0.2.

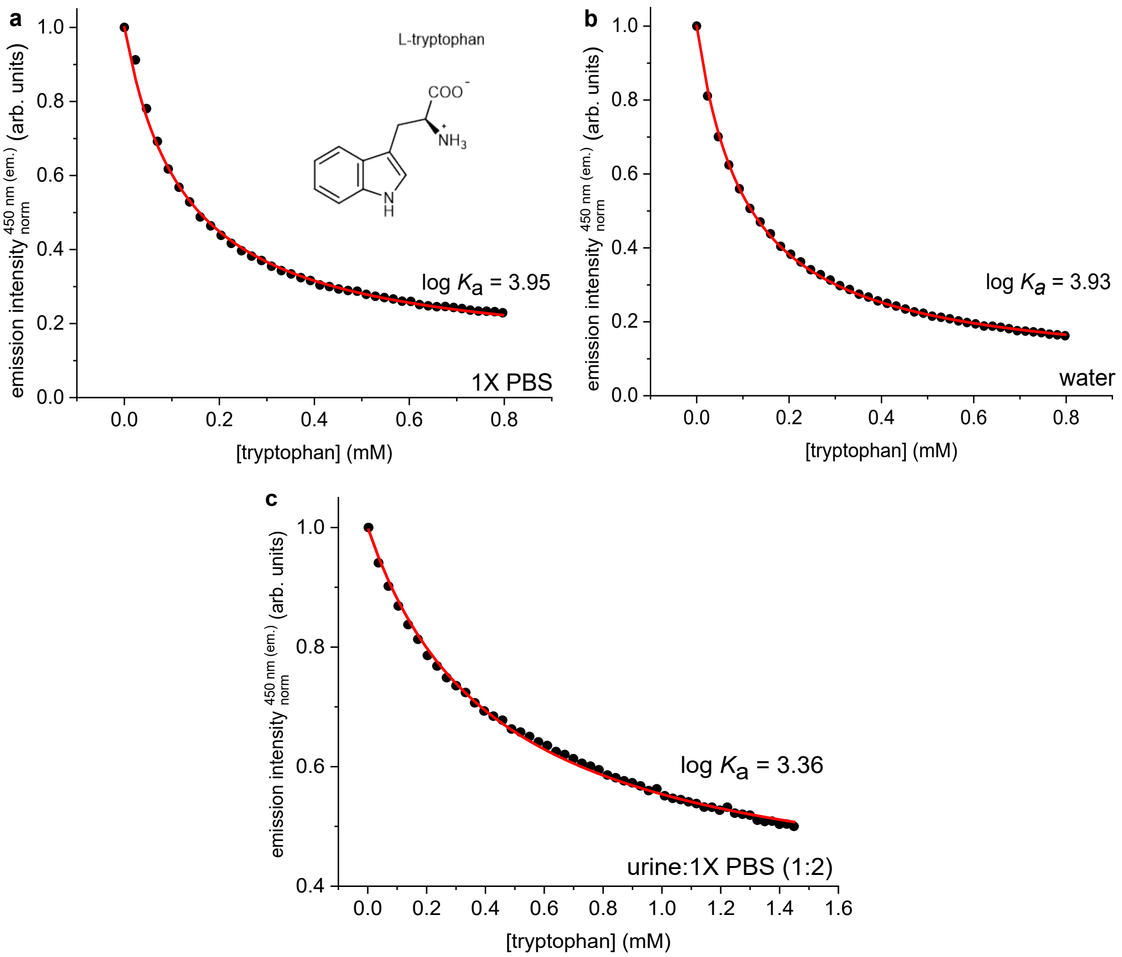

Supplementary Fig. 6. Representative DBA binding isotherms determined by emission-based titration measurements (λ_{ex} = 393 nm, λ_{em} = 450 nm): **a** tryptophan (c = 0 - 797 µM) to rotaxane **1** (c = 2.5 µM) in 1X PBS at 25 °C, **b** tryptophan (c = 0 - 797 µM) to rotaxane **1** (c = 2.5 µM) in water at 25 °C, and **c** tryptophan (c = 0 - 1.44 mM) to rotaxane **1** (c = 2.5 µM) in urine (1:2 in 1X PBS) at 25 °C. Black circles depict the acquired data. The fit according to a 1:1 binding model is shown as red line. Log K_a was determined in at least triplicates and therefore errors in log K_a are estimated to be not larger than 0.2.

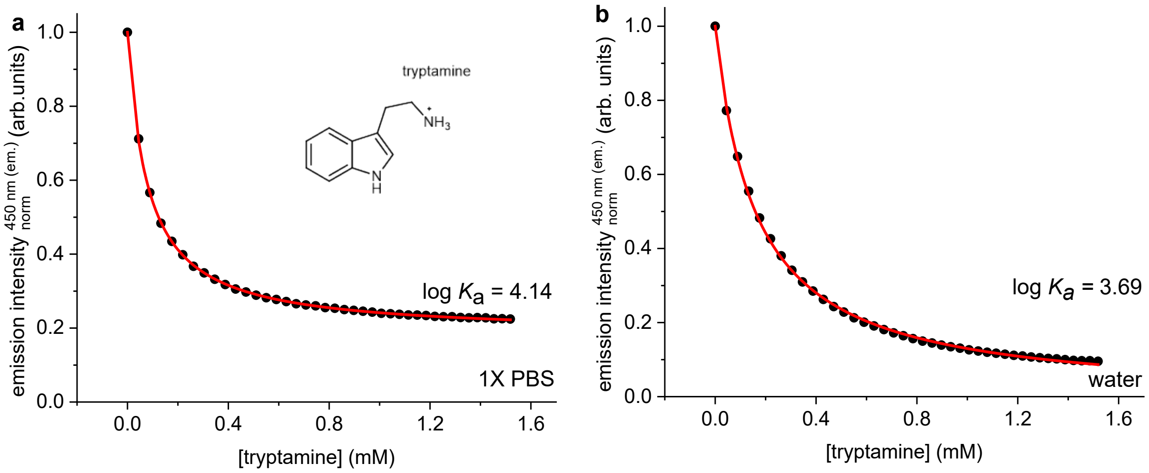

Supplementary Fig. 7. Representative DBA binding isotherms determined by emission-based titration measurements (λ_{ex} = 393 nm, λ_{em} = 450 nm): **a** tryptamine (c = 0 - 1.5 mM) to rotaxane **1** (c = 2.5 µM) in 1X PBS at 25 °C and **b** tryptamine (c = 0 - 1.5 mM) to rotaxane **1** (c = 2.5 µM) in water at 25 °C. Black circles depict the acquired data. The fit according to a 1:1 binding model is shown as red line. Log K_a was determined in at least triplicates and therefore errors in log K_a are estimated to be not larger than 0.2.

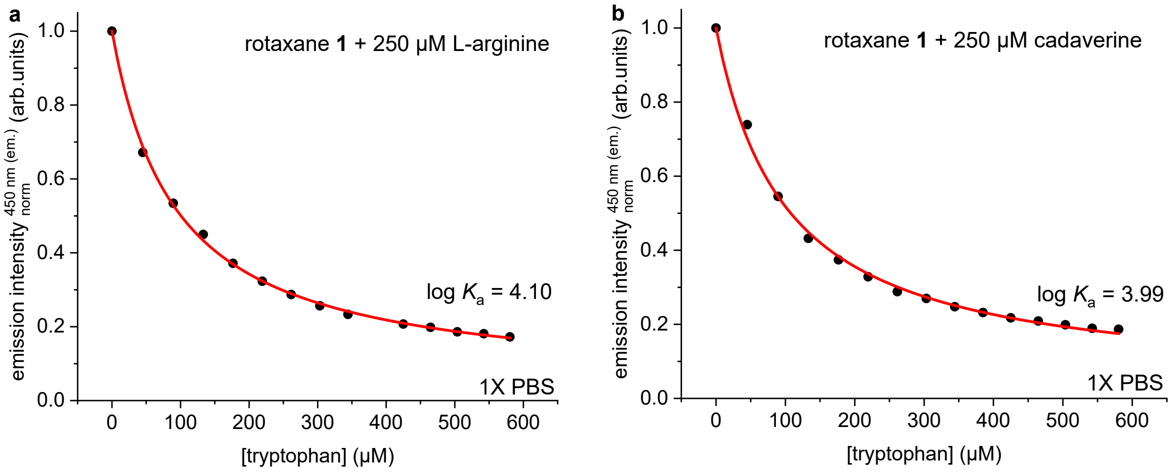

Supplementary Fig. 8. Representative binding isotherms determined by emission-based titration measurements (λ_{ex} = 393 nm, λ_{em} = 450 nm) of tryptophan (c = 0 - 581 µM) to rotaxane **1** (c = 2 µM) in the presence of **a** 250 µM L-arginine and **b** 250 µM cadaverine in 1X PBS at 25 °C. The determined binding affinities for Trp and rotaxane **1** in the presence of the potential interferents did not differ significantly from the binding affinities determined by a direct binding assay of Trp to rotaxane **1**, verifying that neither of the tested analytes influences the sensing abilities of rotaxane **1** towards Trp. Black circles depict the acquired data. The fit according to a 1:1 binding model is shown as red line. Log K_a was determined in at least triplicates and therefore errors in log K_a are estimated to be not larger than 0.2.

3. HPLC-based quantification of L-Trp in serum samples

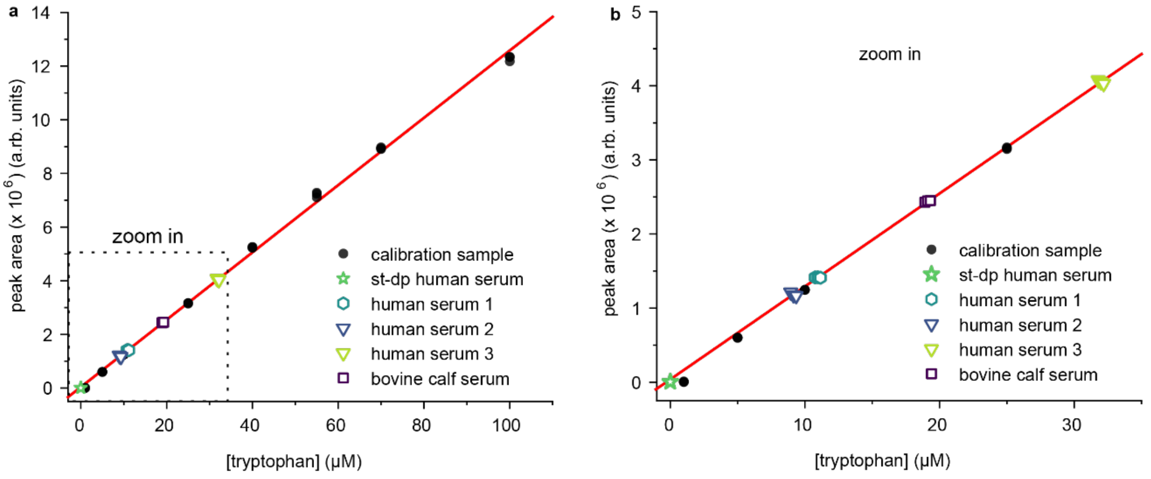

Supplementary Fig. 9. a Plots of the linear calibration curve (black dots and red line) and the integrated peak area of the fluorescence signal (λ_{ex} = 285 nm, λ_{em} = 353 nm) of the unknown serum samples, all measured in n = 3 independent replicates. **b** Zoom in. All independent replicate measurements are shown as individual data points.

4. Rotaxane assay for L-Trp determination in non-deproteinized blood serum samples

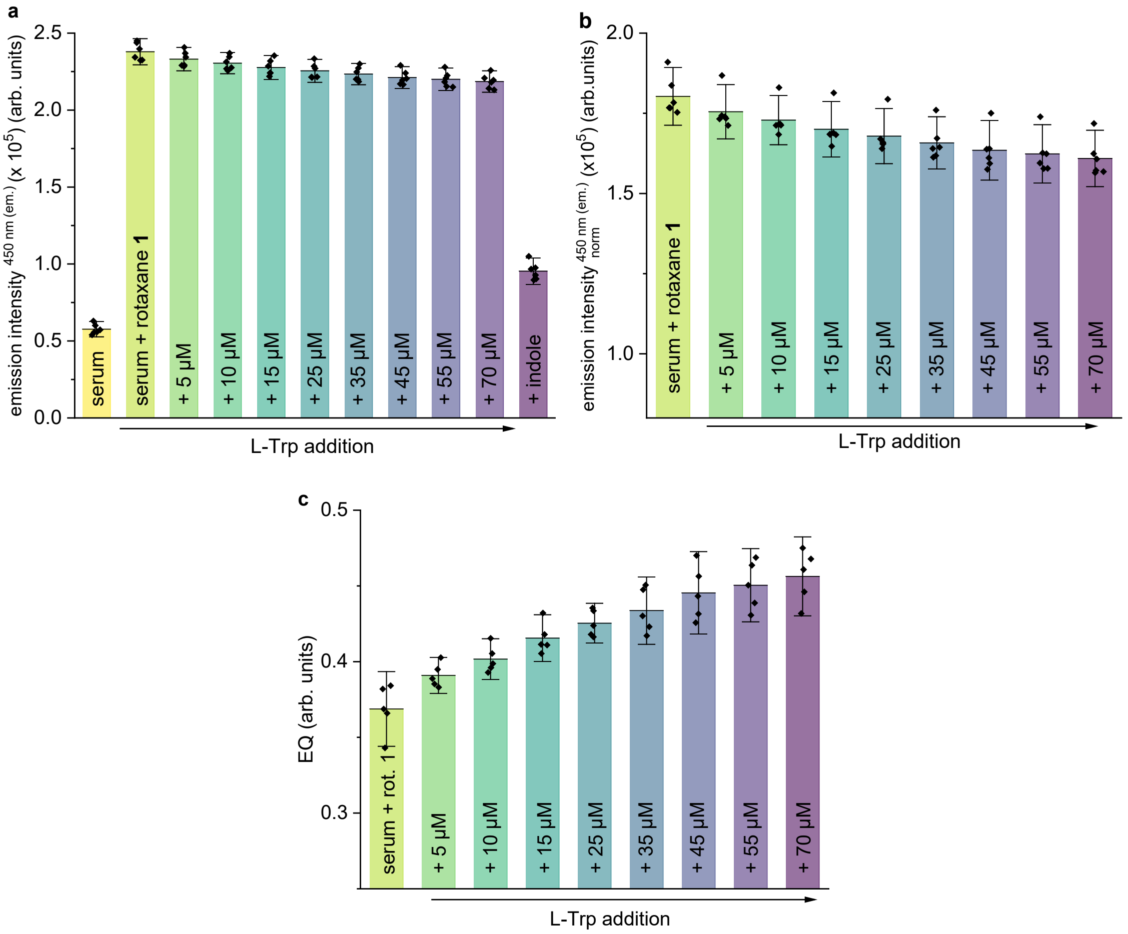

Supplementary Fig. 10. Bar graphs of **a** the emission intensity of the steroid-depleted human serum sample prior (yellow) and after the addition of 10 µM of rotaxane **1** (light green), after stepwise spiking with Trp (green to blue), and after indole addition (dark violet). **b** Bar graphs of emission intensities of rotaxane **1** upon the Trp addition corrected for the auto emission of the serum sample and **c** emission quenching of rotaxane **1** in serum. The excitation wavelength $\lambda_{ex} = 393$ nm and the emission wavelength $\lambda_{em} = 450$ nm were used. All measured data points are shown; bar graph height indicates the mean (middle line), and error bars represent the standard deviation, with n = 5 independent replicates (coefficient 1.5).

5. HPLC-based quantification of L-Trp in urine samples

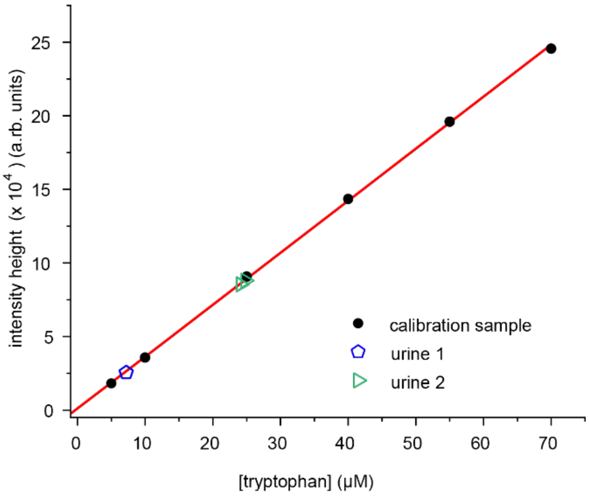

Supplementary Fig. 11. Plot of the linear calibration curve (black dots and red line) and the intensity height of the fluorescence signal (λ_{ex} = 295 nm, λ_{em} = 340 nm) of the unknown urine samples, measured in n = 2 independent replicates for urine sample 1 and n = 3 independent replicates for urine sample 2 and for the Trp standard solutions.

6. Rotaxane assay for L-Trp determination in urine samples

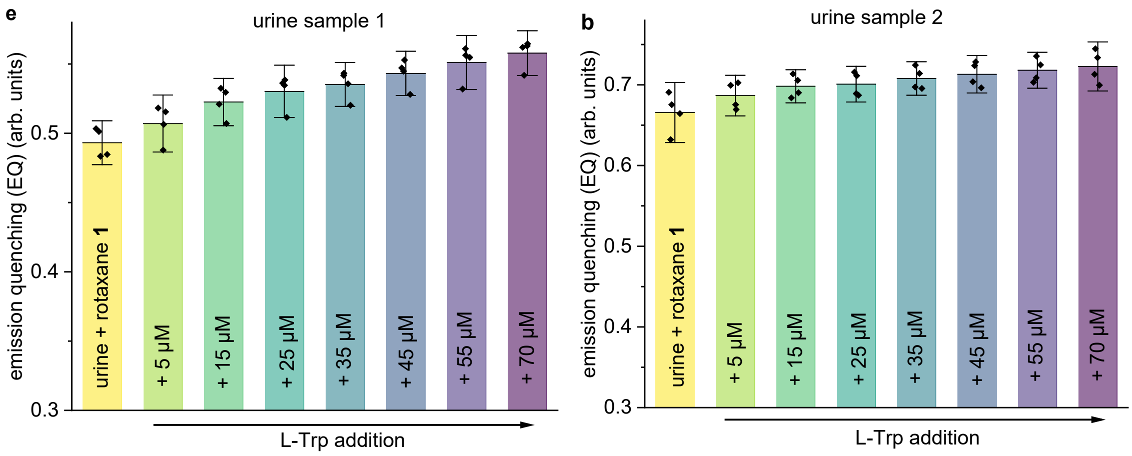

Supplementary Fig. 12. Bar graphs of the emission quenching at λ_{em} = 450 nm (λ_{ex} = 393 nm) of rotaxane **1** (yellow) in **a** urine sample 1 and **b** urine sample 2 upon adding different amounts of Trp (green to violet). All measured data points are shown. Bar graph height indicates the mean (middle line), and error bars represent the standard deviation, with n = 4 independent replicates (coefficient 1.5). Trp concentrations of urine 1 (c = 7.2 ± 0.7 µM) and urine 2 (c = 24.3 ± 2.4 µM) were determined using HPLC.

7. Monitoring of label-free enzymatic reactions in real time

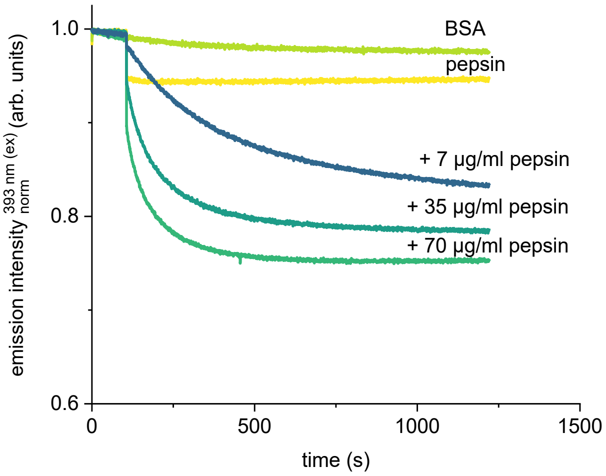

Supplementary Fig. 13. Fluorescence kinetic traces for the enzymatic hydrolysis of 100 µg/mL BSA in the presence of rotaxane **1** (c = 4.5 µM) by pepsin (c = 0 - 70 µg/mL, dark blue to dark green) monitored at 450 nm in 1X PBS (pH 2.0) at 25 °C. BSA cannot bind to rotaxane **1** due to its compact structure in which hydrophobic amino acids such as Phe and Trp are buried inside the protein core. However, the endopeptidase pepsin digests the protein into smaller peptides carrying exposed amino acids on their surface, which can be bound and thus detected by the rotaxane chemosensor. The expected increase in the hydrolysis rate of BSA with increasing pepsin concentration was observed.

8. Rotaxane microarrays for analyte detection

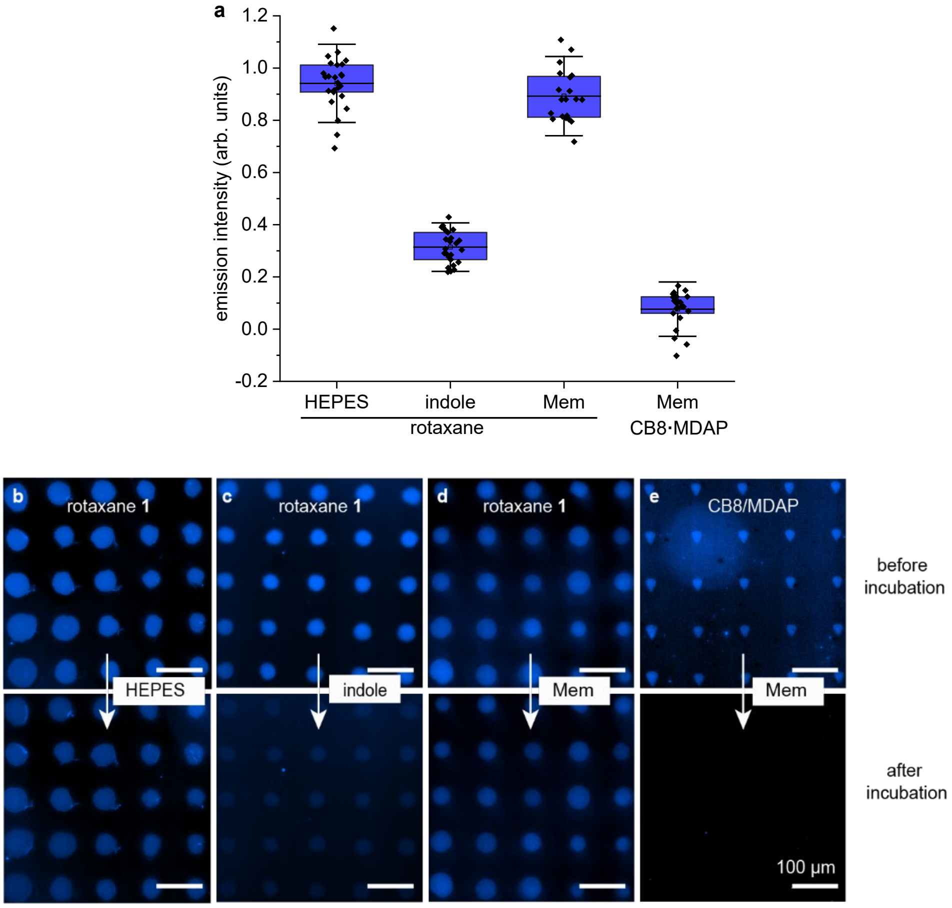

Supplementary Fig. 14. Detection of different analytes with rotaxane **1** and CB8•MDAP microarrays. **a** Quantification of the fluorescence intensities of the microarray spots before and after the incubation with different analytes. Box plots indicate the mean (middle line), 25th, 75th percentile (box) and the standard deviation (whiskers, with a coefficient of 1.5) of all data points (single points), measurements were performed in n = 3 independent replicates. **b-e** Fluorescence images of the microarrays before and after incubation with an analyte (10 µM) or pure HEPES as control. Images were taken with 10 s exposure time and using a DAPI filter. Scale bars equal 100 µm. Measurements were performed in n = 3 independent replicates per analyte.

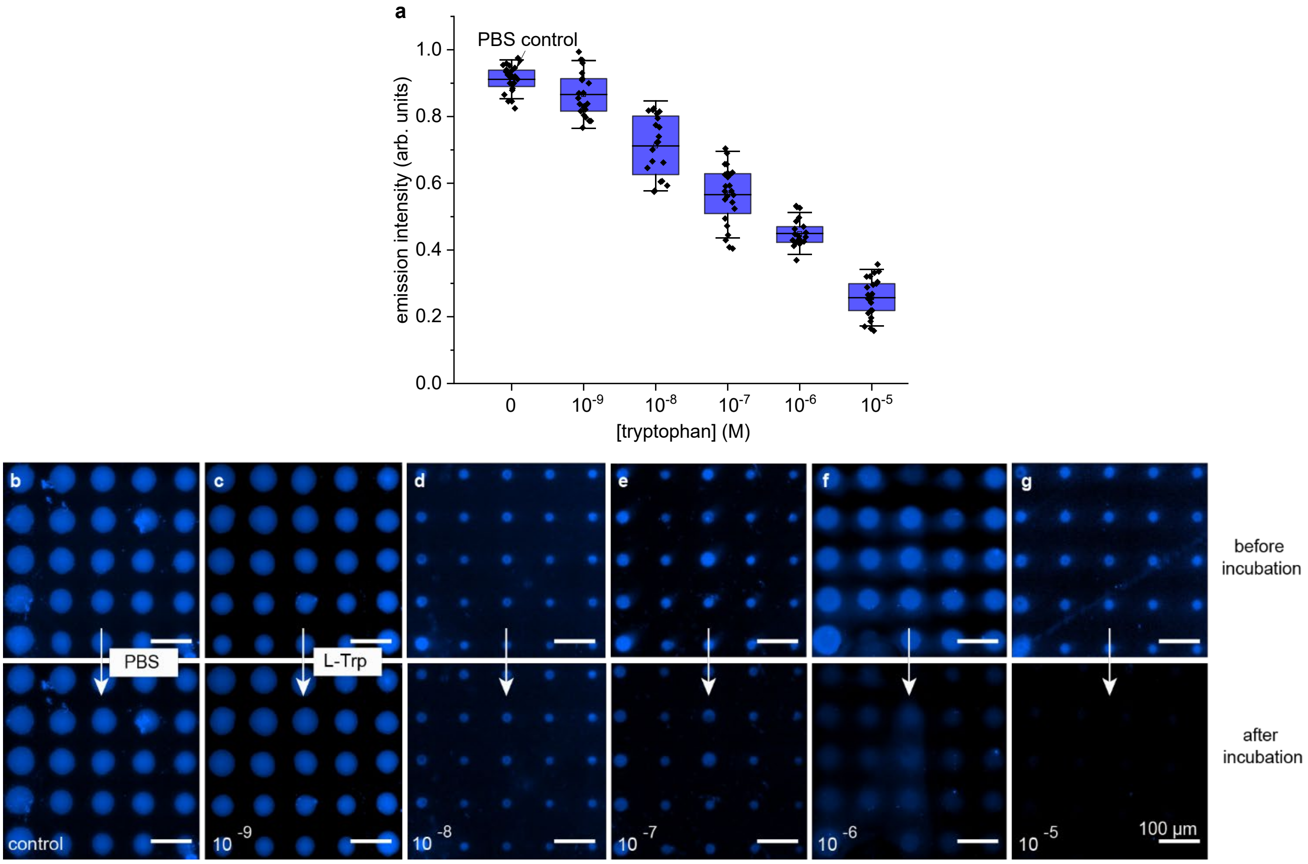

Supplementary Fig. 15. Tryptophan detection at different concentrations in 1X PBS on rotaxane **1** microarrays with n =3 independent replicates. **a** Box plot of the quantification of the fluorescence intensities of the rotaxane **1** microarray spots before and after the incubation with tryptophan solutions of different concentrations and with 1X PBS as control. Box plots indicate the mean (middle line), 25th, 75th percentile (box) and the standard deviation (whiskers, with a coefficient of 1.5) of all data points (single points). **b-g** Fluorescence images of the rotaxane **1** microarrays before and after incubating with tryptophan solutions of various concentrations. Images were taken with 10 s exposure time and a DAPI filter. Scale bars equal to 100 µm. Images were obtained in n = 3 independent replicates per Trp concentration.

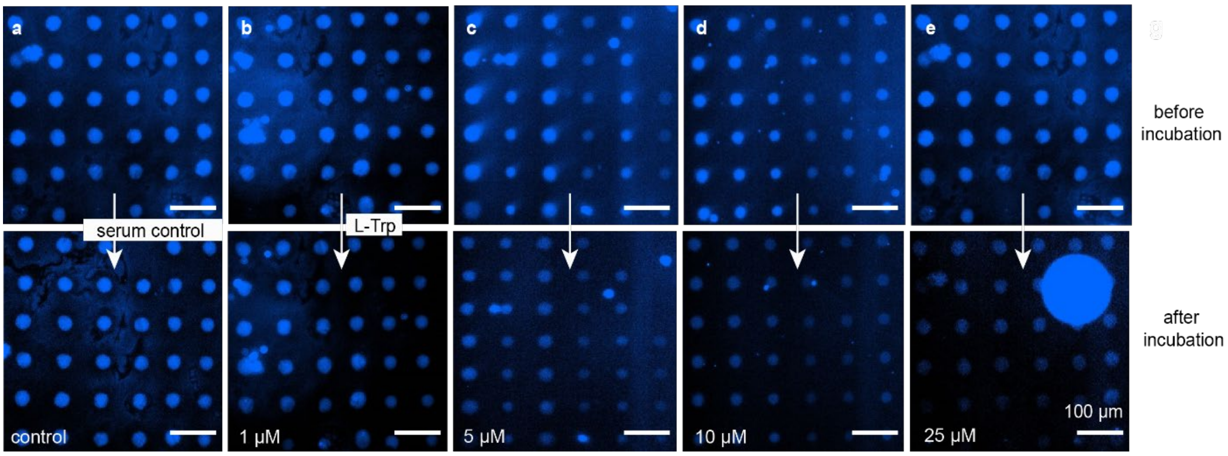

Supplementary Fig. 16. Serum experiments with rotaxane **1** microarrays. **a-f** Fluorescence images of rotaxane **1** microarrays before and after incubating with tryptophan solutions of various concentrations. Images were taken with 10 s exposure time and a DAPI filter. Scale bars equal to 100 µm. Images were obtained in n = 1 measurement per Trp concentration.

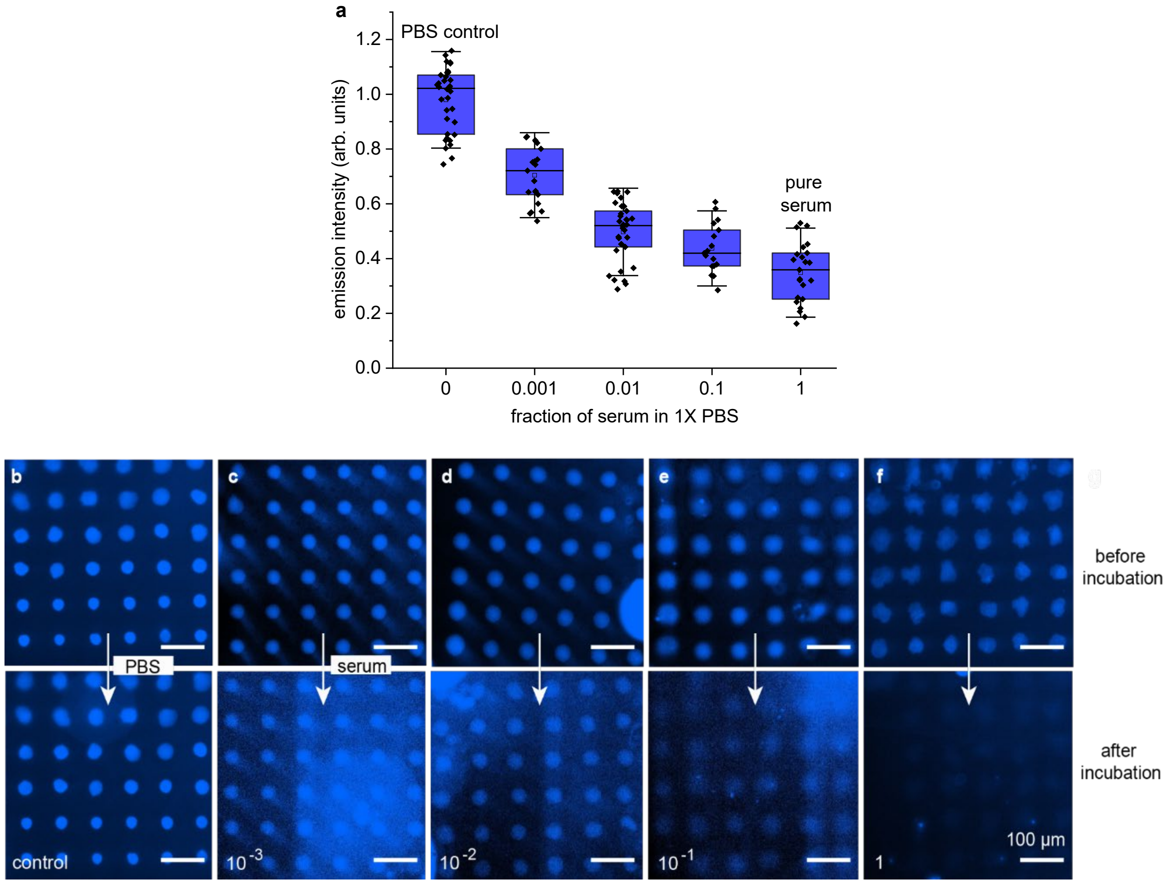

Supplementary Fig. 17. L-Trp detection in human blood serum at different dilutions with 1X PBS on rotaxane **1** microarrays. **a** Box plot of the quantification of the fluorescence intensities of the rotaxane **1** microarray spots before and after the incubation with tryptophan solution of different concentrations and with pure serum with an initial Trp concentration less than 1 µM as control. Box plots indicate the mean (middle line), 25th, 75th percentile (box) and the standard deviation (whiskers, with a coefficient of 1.5) of all data points (single points) with n = 1 measurement per dilution. **b-f** Fluorescence images of the rotaxane **1** microarrays before and after incubating with tryptophan solutions of various concentrations. Images were taken with 10 s exposure time and a DAPI filter. Scale bars equal to 100 µm. Images were obtained in n = 1 measurement per dilution.

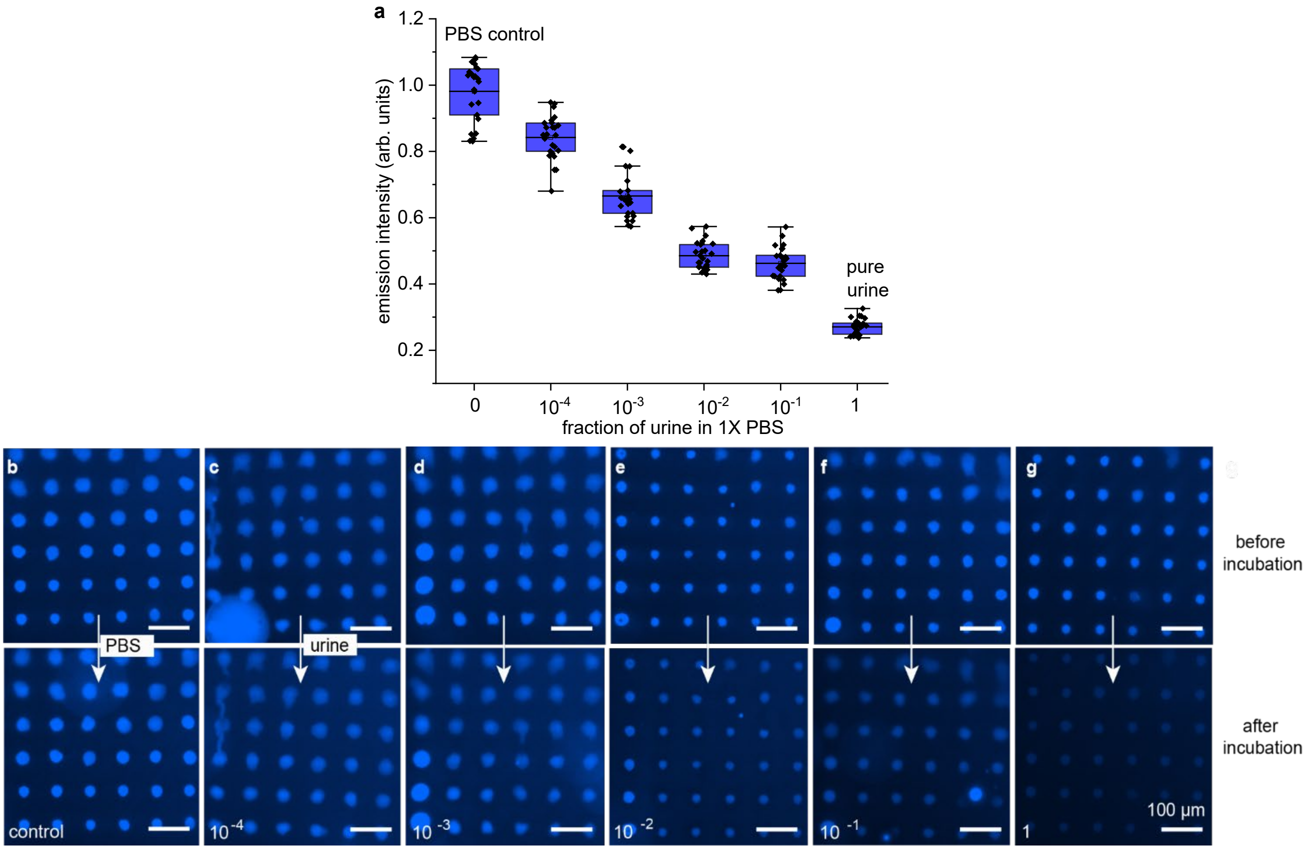

Supplementary Fig. 18. L-Trp detection in human urine at different dilutions with 1X PBS on rotaxane **1** microarrays. **a** Box plot of the fluorescence intensities of the rotaxane **1** microarray spots before and after the incubation with tryptophan solutions of different dilutions and with 1X PBS as control. Box plot indicate mean (middle line), 25th, 75th percentile (box) and the standard deviation (whiskers, with a coefficient of 1.5) of all data points (single points) with n = 1 measurement per concentration. **b-g** Fluorescence images of rotaxane **1** microarrays before and after incubating with tryptophan solutions of various urine fractions in 1X PBS. Images were taken with 10 s exposure time and a DAPI filter. Scale bars equal to 100 µm. Images were obatained in n = 1 measurement per concentration.

9. Representative synthetic binders for Trp

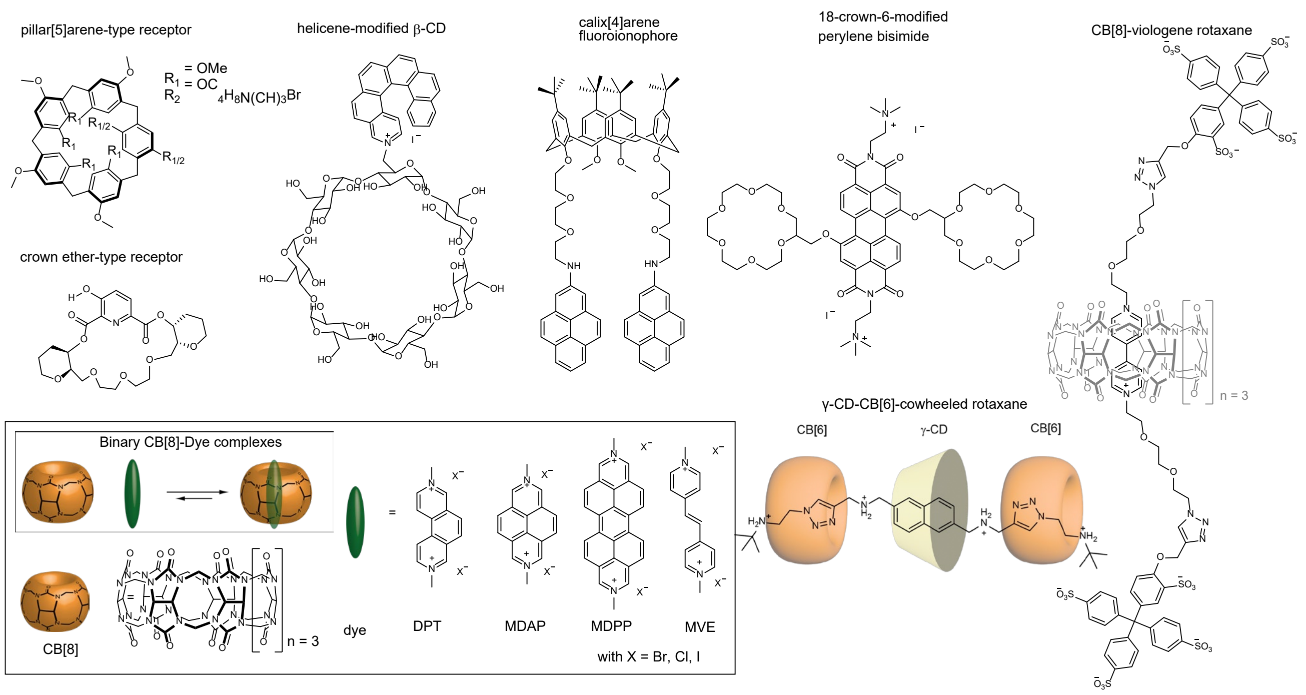

Supplementary Fig. 19. Chemical structures of representative synthetic binders for tryptophan. See also Table 1 in the main text.

Supplementary Notes

1. Selection of commonly used non-covalent supramolecular chemosensors in several aqueous media and biomedia

The following section briefly summarizes the stability and sensing abilities of a selection of commonly used non-covalent supramolecular chemosensors. In these experiments, we wanted to compare their functionality for sensing in aqueous media, saline buffer, and biomedia such as human blood serum. The chemical structures of the selected chemosensor systems are shown in Supplementary Fig. 20. Each system was examined in a 96-well plate format by measuring its emission intensity at 25 °C. The concentration of each of the host components was adjusted to 10 µM to use comparable concentrations to rotaxane **1** in human blood serum (see Supplementary Fig. 23).

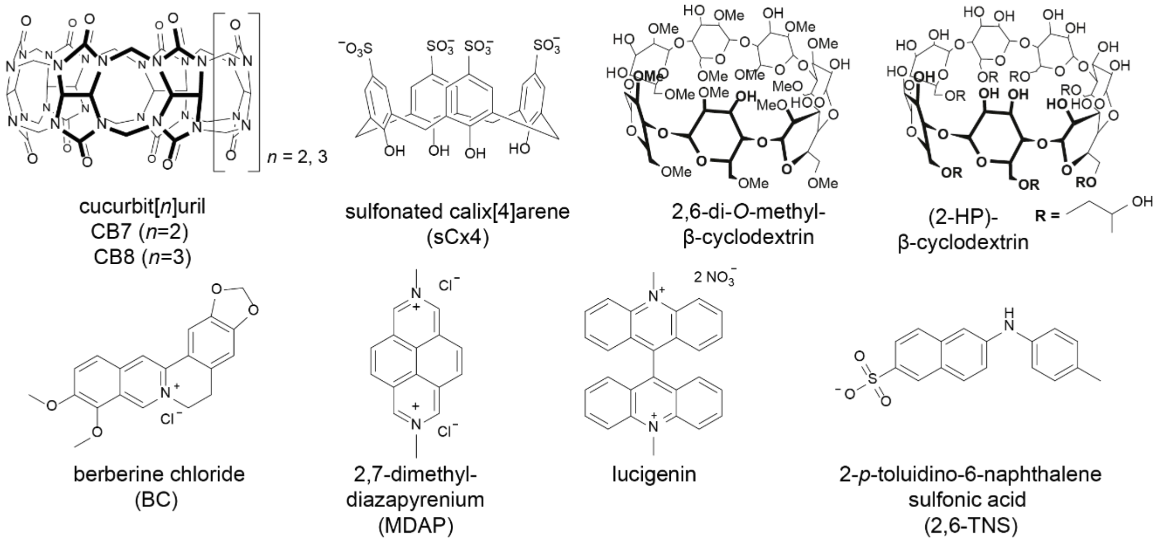

Supplementary Fig. 20. Chemical structure of host (top) and dye (bottom) molecules of the selected chemosensor systems.

All tested dyes show the commonly found emission turn-on when complexed by one of the macrocycles. The supramolecular complexes CB7•berberine chloride and sCx4•lucigenin both show a strong emission signal in water (see black spectra in Supplementary Fig. 21a and b), verifying that they are stable systems in water. However, their transfer to a saline buffer system, *e.g.*, 1X PBS, results in a strongly decreased emission intensity. Their emission intensity is similarly quenched in human blood serum and urine. These results indicate that both supramolecular complexes dissociate in the presence of salts due to the competitive binding of cations (CB*n* portals and calix cavity). The quenching of lucigenin by chloride may also play a role.[6,7] Be this as it may, both supramolecular systems cannot be used as chemosensors in saline media or even biofluids. A different problematic behavior was observed for the mixture of β-cyclodextrin as host and 2-*p*-toluidino-6-naphthalenesulfonic acid (2,6-TNS) as dye. While the addition of 2,6-TNS to β-CD and its derivatives usually causes an emission increase in water,[8] we observed in our experiments in the micromolar concentration range no emission enhancement in water, buffers, or urine due to the low binding affinity (*e.g.*, log $K_a \sim 3$).[9] Only in

human blood serum an emission enhancement was observed, which can be attributed to dye binding to the protein human serum albumin.[10,11] These results show that both systems are unsuitable for sensing in aqueous media or biomedia. In addition, the interference of the dye with proteins in human serum further limits its application in biofluids.

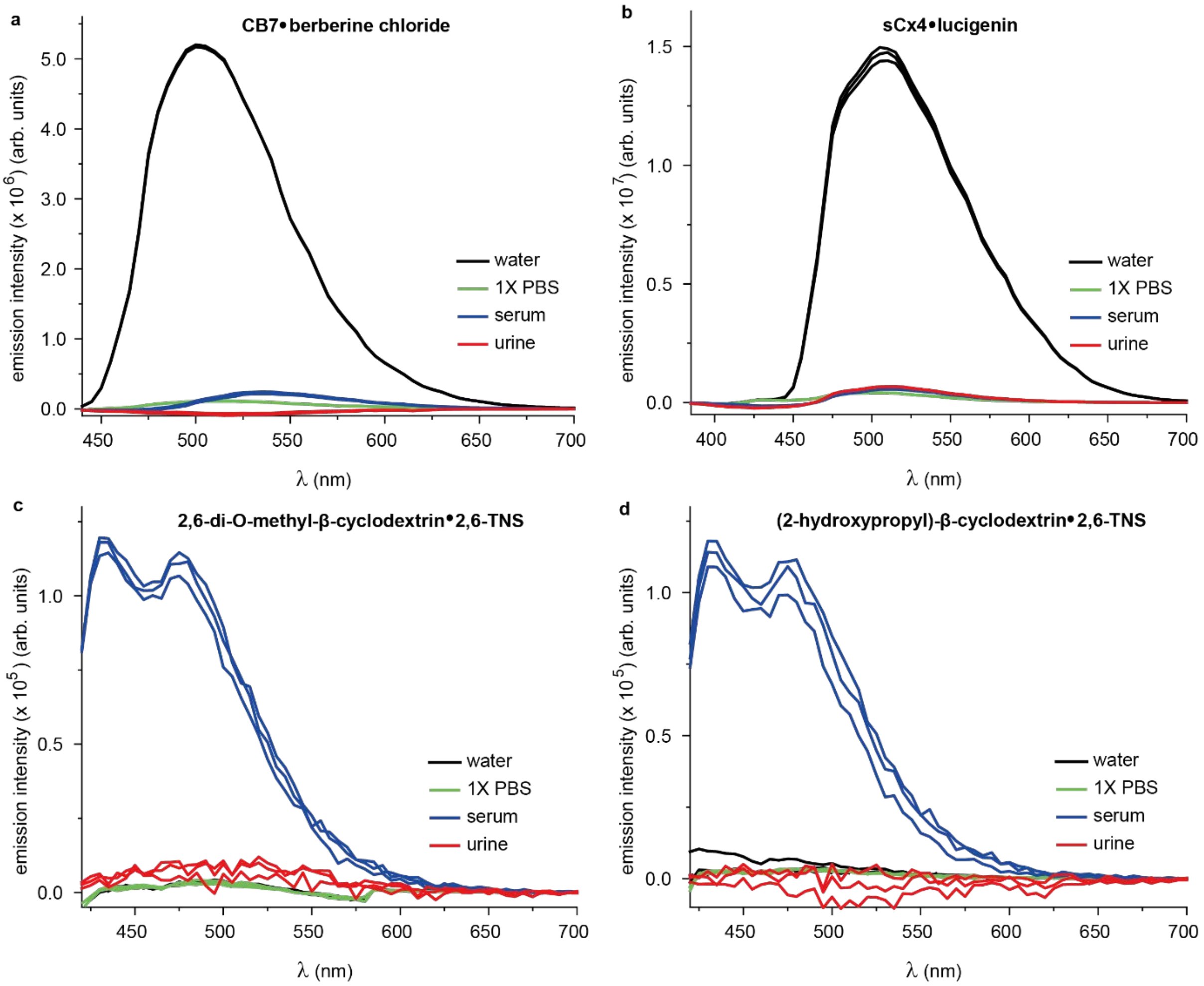

Supplementary Fig. 21. Emission spectra of four selected chemosensor systems at λ_{ex} = 393 nm in water (black line), 1X PBS (green line), human blood serum (blues line), and human urine (red line) with: **a** CB7•BC (c(CB7) = 10 μM, c(BC) = 15 μM), **b** sCx4•lucigenin (c(sCx4) = 10 μM, c(lucigenin) = 15 μM), **c** 2,6-di-O-methyl-β-cyclodextrin•2,6-TNS (c(β-CD) = 10 μM, c(2,6-TNS) = 15 μM), and **d** (2-HP)-β-cyclodextrin•2,6-TNS (c(β-CD) = 10 μM, c(2,6-TNS) = 15 μM). All measurements were conducted in triplicates at 25 °C in a 96-microwell plate format.

Another CB*n*-based supramolecular complex, CB7•MDAP, is less affected by salt cations since CB7 has a larger binding affinity towards the dye 2,7-dimethyldiazapyridinium (MDAP, log K_a = 9.4)[9] compared to berberine chloride (log K_a = 7.2).[9] This system is therefore to a certain extent stable in saline buffers and even human blood serum (see Supplementary Fig. 22), although it displays a reduced emission intensity compared to water as a solvent. Nevertheless, the system is not suitable for sensing low micromolar concentrations of tryptophan (log K_a ~ 3 for CB7 in water), especially not in biomedia due to the presence of many competitive binders such as biogenic amines, steroids, and proteins, and due to the high binding affinity of the indicator dye for the host. For example, Supplementary Fig. 22 shows that the addition of 66-times excess of tryptophan to CB7•MDAP in human blood serum does not significantly alter the emission intensity.

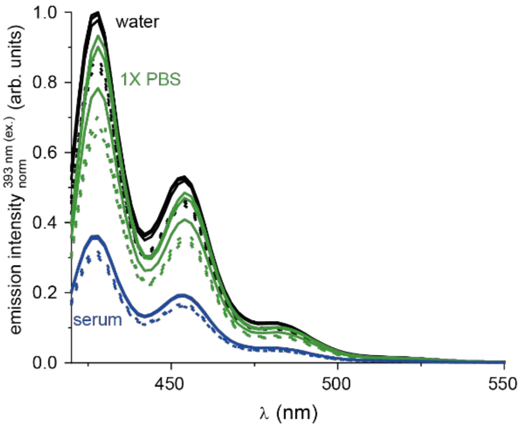

Supplementary Fig. 22. Emission spectra of CB7•MDAP (c(CB7) = 10 µM, c(MDAP) = 15 µM) in water (black), 1X PBS (green), and human blood serum (blue) before (solid line) and after the addition of 655 µM Trp (dashed line). All measurements were conducted in at least duplicates at 25 °C in a 96-microwell plate format.

Unlike CB7, CB8 can bind two analytes at the same time as a result of its bigger cavity size.[12] This unique property allows the formation of 1:1:1 complexes consisting of the macrocycle CB8, a dye, and an analyte. In this case, the interaction of the dye with the analyte inside the cavity of the CB8 leads to a quenching of the dye's emission. We compared rotaxane **1** with the non-covalent chemosensing system CB8•MDAP as both contain the same dye moiety. In short, the emission intensity of CB8•MDAP and rotaxane 1 were measured upon the stepwise addition of tryptophan (c = 0 - 425 µM) in water, 1X PBS, and human serum at λ_{em} = 450 nm (λ_{ex} = 393 nm) (see Supplementary Fig. 23). Both systems show a significant emission response upon the addition of tryptophan in water (black dots) and 1X PBS (blue triangles).

In human blood serum, the initial emission intensity of CB8•MDAP is more than 20% reduced compared to rotaxane **1**. Moreover, the observed emission quenching upon the addition of an excess Trp (> 400 µM) is significantly larger for rotaxane 1 than for CB8•MDAP, see Supplementary Fig. 23. Therefore, rotaxane 1 offers a larger useable detection window, *i.e.*, the partial emission quenching per tryptophan unit, compared to CB8•MDAP, which makes the rotaxane more sensitive for smaller amounts of tryptophan in a more extended concentration range. In addition, the salt influence ("matrix effect") on the tryptophan detection of both chemosensor systems was evaluated by measuring the emission intensity quenching of a 25 µM tryptophan

solution in the presence of different amounts of sodium chloride. The results are shown in Supplementary Fig. 23. The colored boxes illustrate the disturbing influence of an unknown salt concentration on the sensing abilities of the corresponding chemosensors. The results demonstrate that rotaxane **1** is less affected by salts than CB8•MDAP. In addition, the integrity of CB8•MDAP, but not of rotaxane **1**, is destroyed in the presence of hydrophobic interferents (see also Supplementary Fig. 4).

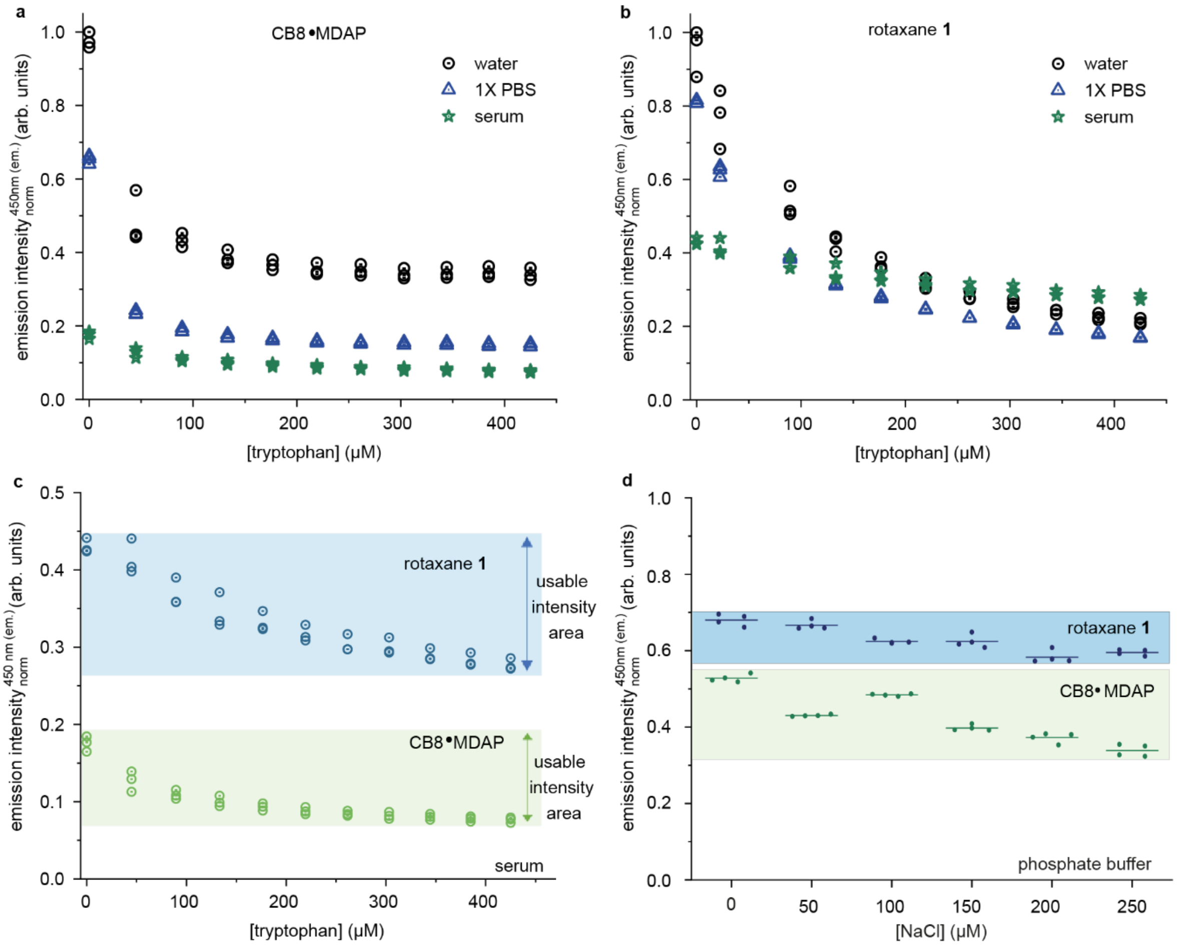

Supplementary Fig. 23. Emission-based titration measurements (λ_{em} = 450 nm) of Trp (c = 0 - 425 μM) to **a** CB8•MDAP (c(CB8) = 10 μM, c(MDAP) = 11 μM) and **b** rotaxane **1** (c = 10 μM) in water (black dots), 1X PBS (blue triangles), and human blood serum (green stars). **c** Emission-based titration measurements (λ_{em} = 450 nm) of Trp (c = 0 - 425 μM) to rotaxane **1** (c = 10 μM, blue dots) and CB8•MDAP (c(CB8) = 10 μM, c(MDAP) = 11 μM, green dots) in human blood serum. The colored boxes indicate the available intensity region for the corresponding chemosensor. **d** Quenching intensity (λ_{em} = 450 nm) of 25 μM tryptophan for rotaxane **1** (c(**1**) = 10 μM) and CB8•MDAP (c(CB8) = 10 μM, c(MDAP) = 11 μM) in the presence of different amounts of sodium chloride in 10 mM sodium phosphate buffer at pH 7.7. All measurements were conducted in at least triplicates at 25 °C in a 96-microwell plate format.

2. Structured immobilization by microcontact printing of rotaxane 1

The immobilization of rotaxane **1** was conducted using microchannel cantilever spotting (μCS). For a detailed description of the experimental setup, see the methods section in the main part. As control experiments, a CB8•MDAP chemosensor microarray was set up according to previous work.[13] CB8 ink containing 1 mg/mL of propargyl-functionalized CB8 (in DMSO containing 40% TCEP dissolved in water (3 mg/mL) and 20% glycerol) was micropatterned on thiol surfaces (SiO2–SH) via μCS to obtain the CB8 microarray. After printing, the substrate was irradiated with UV light (254 nm) for 10 min, washed with water and ethanol, and dried by a N_2 stream. The remaining free thiols of the surface were blocked through incubation with an aqueous *N*-ethylmaleimide (10mg/mL, blocking agent) solution at pH 7.0 for 2 h. Then, the substrate was covered with 20 μL of a 50 μM MDAP solution for 1 min, washed with water and dried with an N_2 stream to obtain the CB8•MDAP chemosensor array.

Initial test of analyte detection

The response of the CB8•MDAP microarray towards the presence of an analyte is shown in comparison to the response of rotaxane **1** microarrays with different analytes in HEPES buffer (see Supplementary Fig. 14). The incubation of the rotaxane **1** array with pure HEPES buffer as a negative control shows no significant decrease in fluorescence. As expected, the emission intensity of rotaxane **1** decreased for the incubation with indole showing that indole binds to the immobilized rotaxane **1** and thereby quenches the emission of the DAP dye. In contrast, the incubation of the rotaxane **1** microarray with memantine, an analyte with a larger binding affinity towards CB8 than DAP, shows only an insignificant emission change, indicating that the analyte is not bound. In contrast, the CB8•MDAP microarray shows an almost 100% emission quenching upon incubation with memantine (Mem), indicating that the MDAP dye is displaced by the bigger and stronger binding analyte memantine.[13]

Sensitivity tests

The sensitivity of the rotaxane **1** microarrays for analyte detection was first examined by incubation with L-tryptophan solutions at different concentrations in 1X PBS. The emission intensities of the spots of the microarrays before and after the incubation with the analyte and the corresponding fluorescence images are shown in Supplementary Fig. 15. It is possible to detect tryptophan concentrations down to 10^{-8} M, which is indicated by the greater emission decrease of the rotaxane **1** microarray after the incubation with 10^{-8} M compared to the control with 1X PBS. However, a concentration of 10^{-9} M of L-tryptophan is no longer discernible from pure 1X PBS.

Measurements in serum

Besides that, the Trp detection in spiked serum was established. A human blood serum sample with a Trp concentration lower than 1 µM (quantified via HPLC) was spiked with 1, 5, 10 and 25 µM of Trp. The detection was done by incubation of the rotaxane **1** microarrays with the Trp-spiked serum solutions, and the obtained emission intensities are shown in Supplementary Fig. 16. The plotted emission intensities in Supplementary Fig. 16a show a quantitative emission switch-off with increasing Trp concentration and thereby confirm the applicability of the rotaxane **1** microarray.

The applicability of the rotaxane **1** microarrays was then examined by incubation with untreated human blood serum in its untreated form and was diluted with 1X PBS. The untreated, pure serum sample contained around 58 µM Trp, quantified by analytical HPLC (see section 3). The emission intensities of the spots of the microarrays before and after the incubation with the corresponding analyte and the corresponding fluorescence images are shown in Supplementary Fig. 17. The bigger emission decrease of the rotaxane **1** microarray after the incubation with serum and diluted serum compared to the control with 1X PBS shows that it is possible even to detect small amounts of Trp containing serum in 1X PBS. Even undiluted serum (with the highest concentration of potential interfering small molecules/proteins) is not hindering Trp detection.

Urine measurements

The applicability of the rotaxane **1** microarrays was further examined by incubation with a human urine sample that contains ~ 60 µM tryptophan (determined via HPLC) at different dilutions with 1X PBS. The emission intensities of the spots of the microarrays before and after the incubation with urine at various dilutions and the corresponding fluorescence images are shown in Supplementary Fig. 18. It proves that it is possible to detect tryptophan in urine as a biofluid which is indicated by the greater emission decrease of the rotaxane **1** microarray after the incubation with urine compared to the control with 1X PBS.

3. NMR and ESI MS spectra

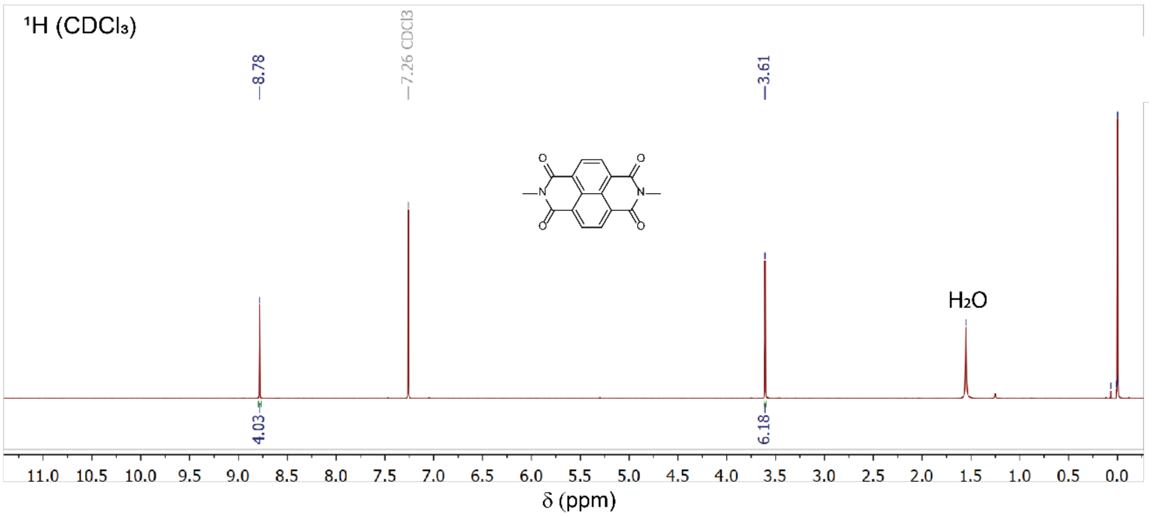

Supplementary Fig. 24. ^{1}H NMR spectrum (500 MHz) of 2,7-dimethylbenzo[*lmn*][3,8]phenanthroline-1,3,6,8-(2*H*,7*H*)-tetraon (**2**) in $CDCl_3$.

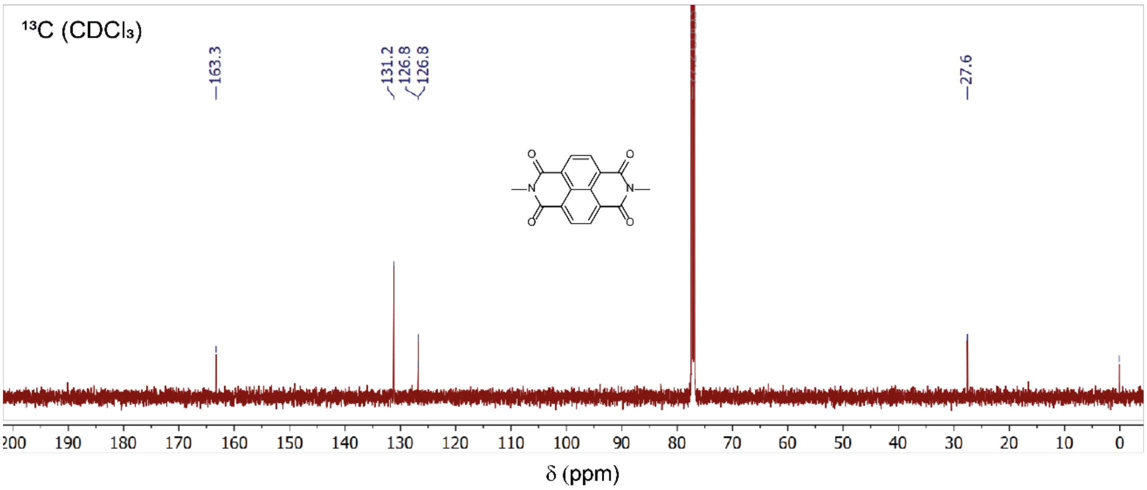

Supplementary Fig. 25. ^{13}C NMR spectrum (126 MHz) of 2,7-dimethylbenzo[*lmn*][3,8]phenanthroline-1,3,6,8-(2*H*,7*H*)-tetraon (**2**) in $CDCl_3$.

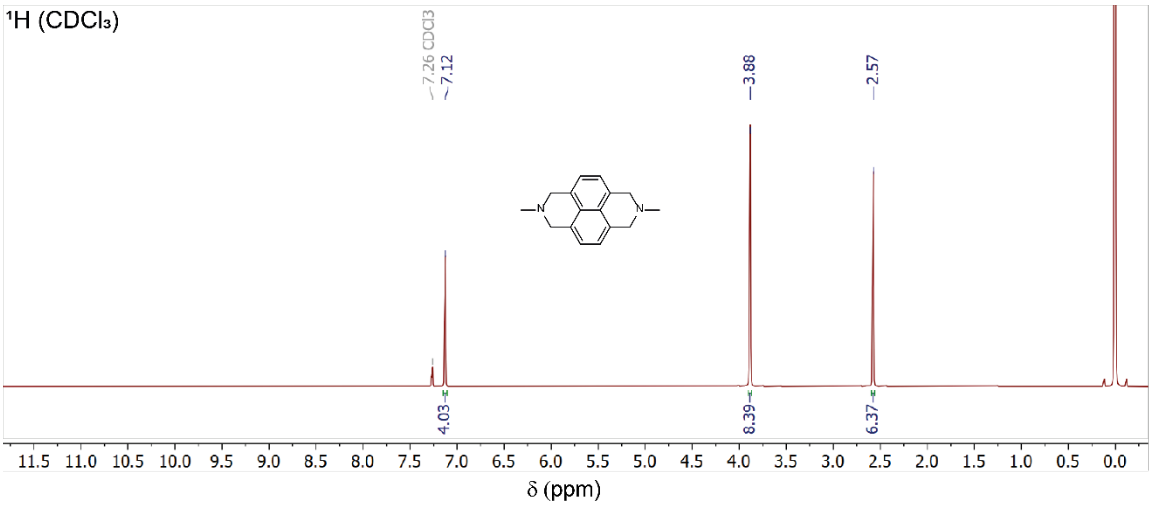

Supplementary Fig. 26. ^{1}H NMR spectrum (500 MHz) of 2,7-dimethyl-1,2,3,6,7,8-hexahydrobenzo[*lmn*][3,8]-phenanthroline (**3**) in $CDCl_3$.

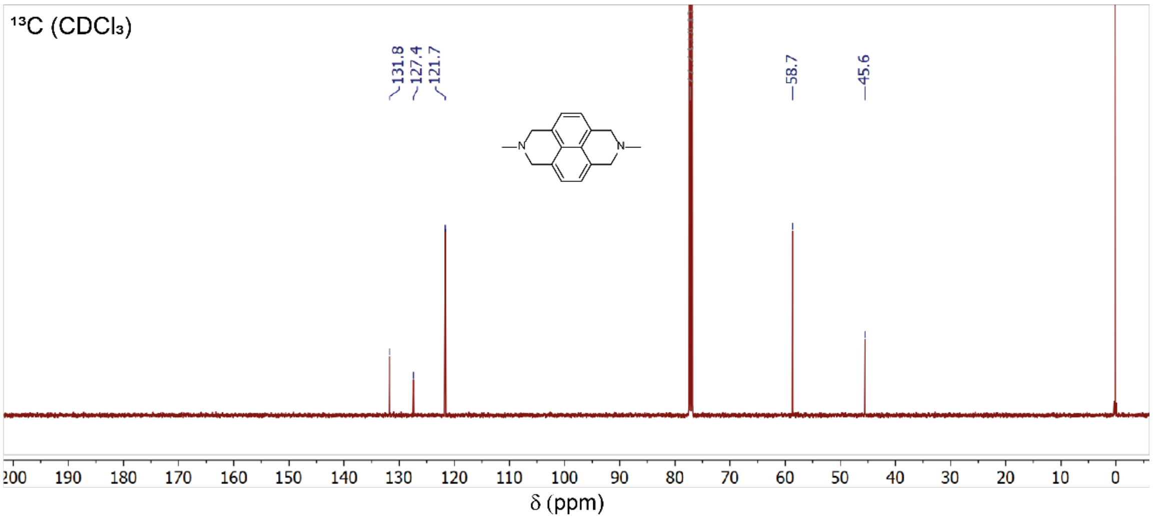

Supplementary Fig. 27. ^{13}C NMR spectrum (126 MHz) of 2,7-dimethyl-1,2,3,6,7,8-hexahydrobenzo[*lmn*][3,8]-phenanthroline (**3**) in $CDCl_3$.

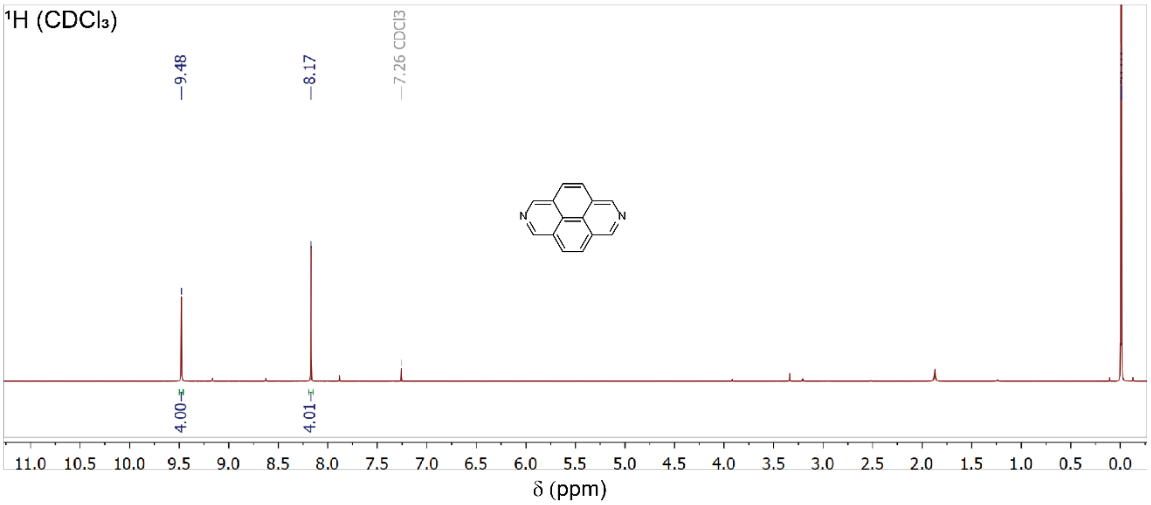

Supplementary Fig. 28. ^{1}H NMR spectrum (500 MHz) of benzo[*lmn*][3,8]phenanthroline (DAP) (**4**) in $CDCl_3$.

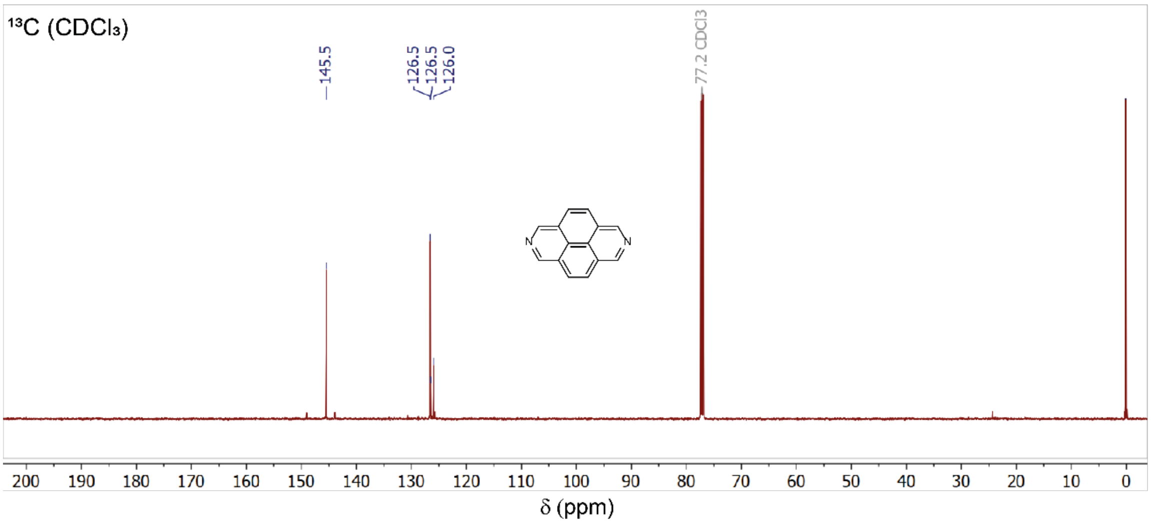

Supplementary Fig. 29. ^{13}C NMR spectrum (126 MHz) of benzo[*lmn*][3,8]phenanthroline (DAP) (**4**) in $CDCl_3$.

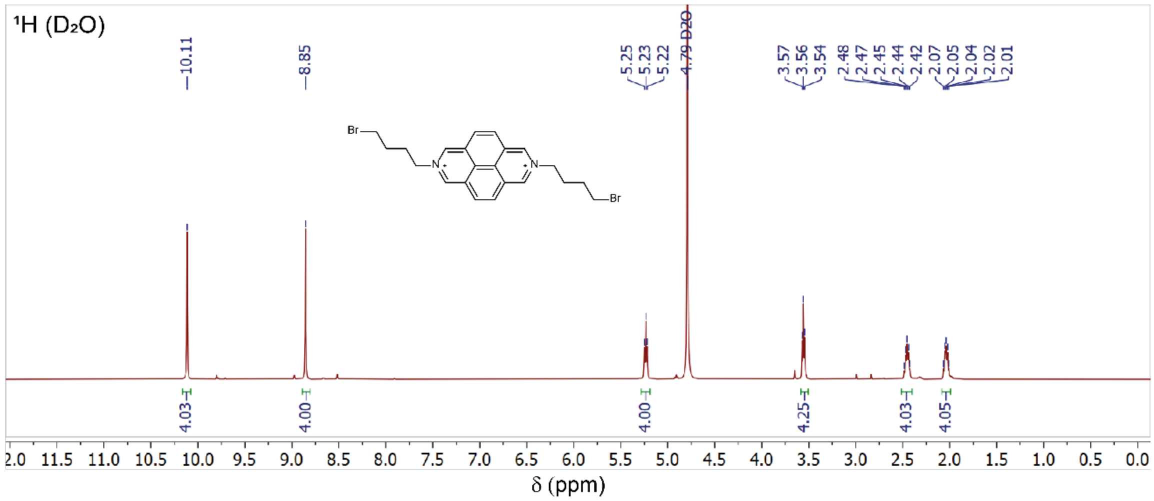

Supplementary Fig. 30. ^{1}H NMR spectrum (500 MHz) of 2,7-bis(4-bromobutyl)benzo[*lmn*][3,8]phenanthroline-2,7-diium dibromide (**5**) in D_2O.

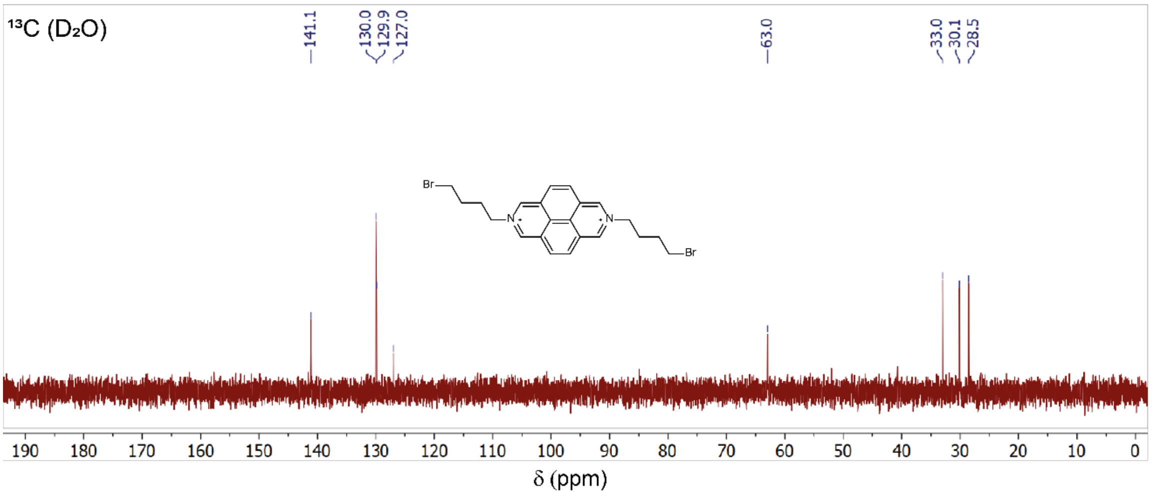

Supplementary Fig. 31. ^{13}C NMR spectrum (126 MHz) of 2,7-bis(4-bromobutyl)benzo[*lmn*][3,8]phenanthroline-2,7-diium dibromide (**5**) in D_2O.

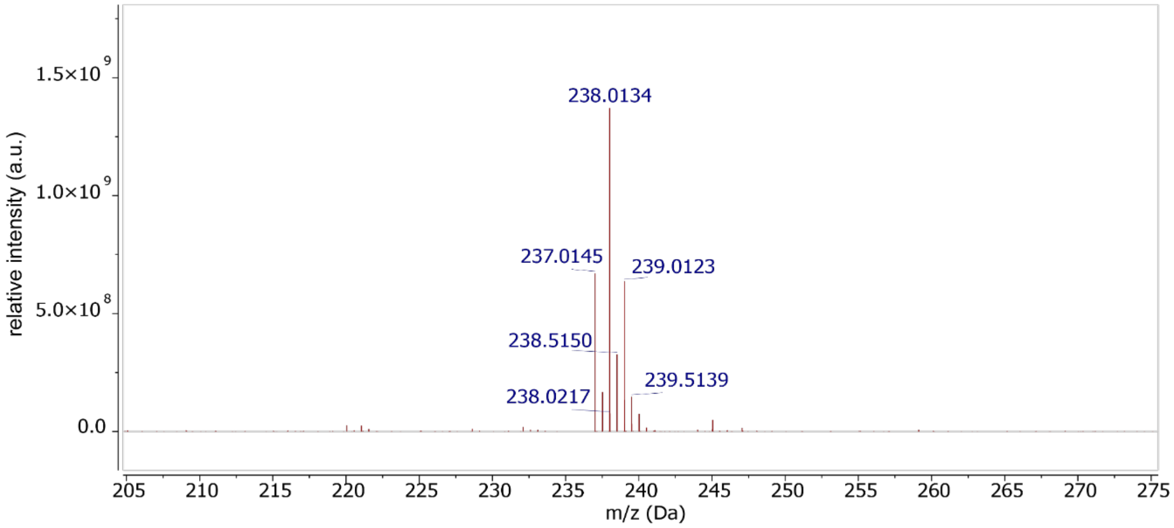

Supplementary Fig. 32. ESI MS spectra of 2,7-bis(4-bromobutyl)benzo[*lmn*][3,8]phenanthroline-2,7-diium dibromide (**5**).

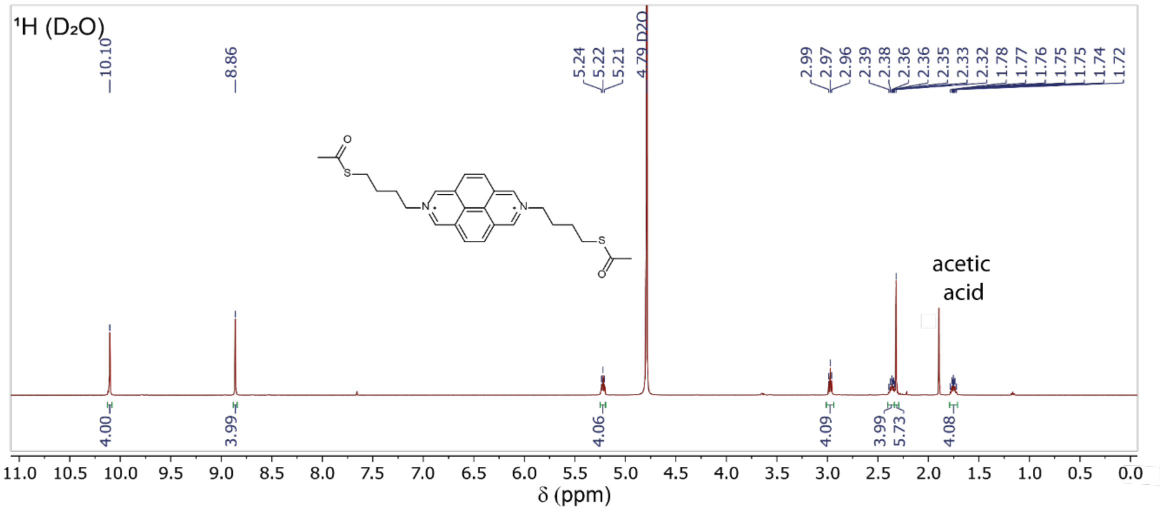

Supplementary Fig. 33. ^{1}H NMR spectrum (500 MHz) 2,7-bis(4-(acetylthio)butyl)benzo[*lmn*][3,8]phenanthroline-2,7-diium dibromide (**6**) in D_2O.

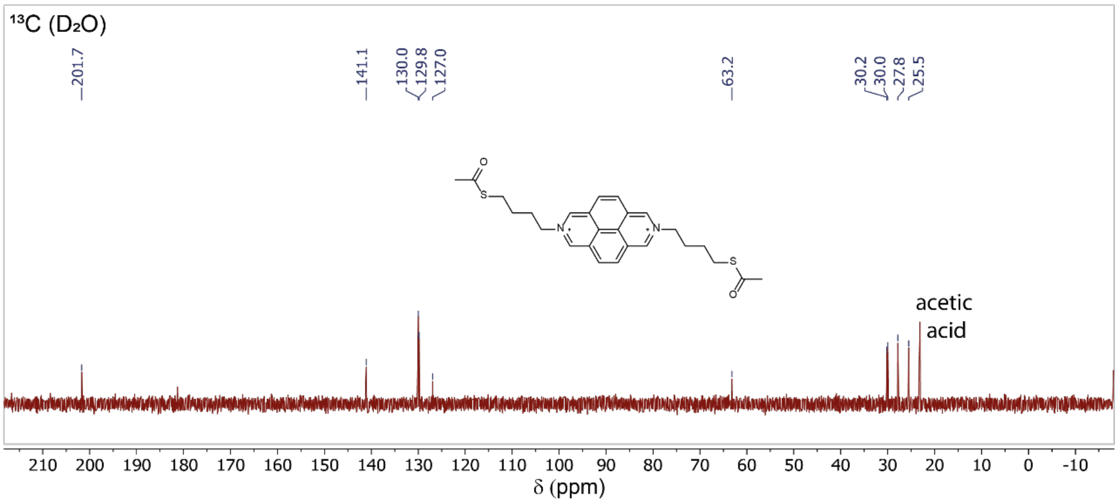

Supplementary Fig. 34. ^{13}C NMR spectrum (126 MHz) of 2,7-bis(4-(acetylthio)butyl)benzo[*lmn*][3,8]phenanthroline-2,7-diium dibromide (**6**) in D_2O.

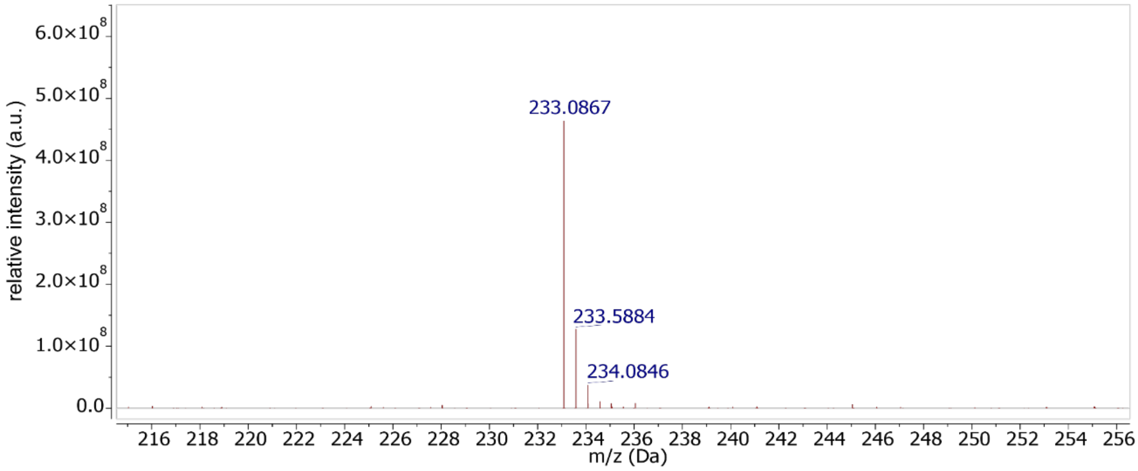

Supplementary Fig. 35. ESI MS spectra of 2,7-bis(4-(acetylthio)butyl)benzo[*lmn*][3,8]phenanthroline-2,7-diium dibromide (**6**).

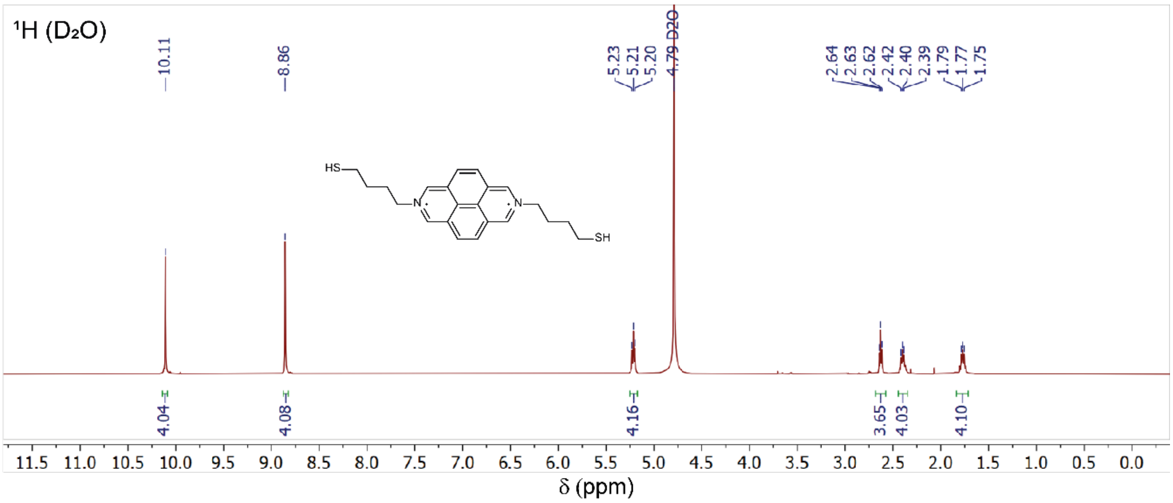

Supplementary Fig. 36. ^{1}H NMR spectrum (500 MHz) of 2,7-bis(4-mercaptobutyl)benzo[*lmn*][3,8]phenanthroline-2,7-diium dibromide (**7**) in D_2O.

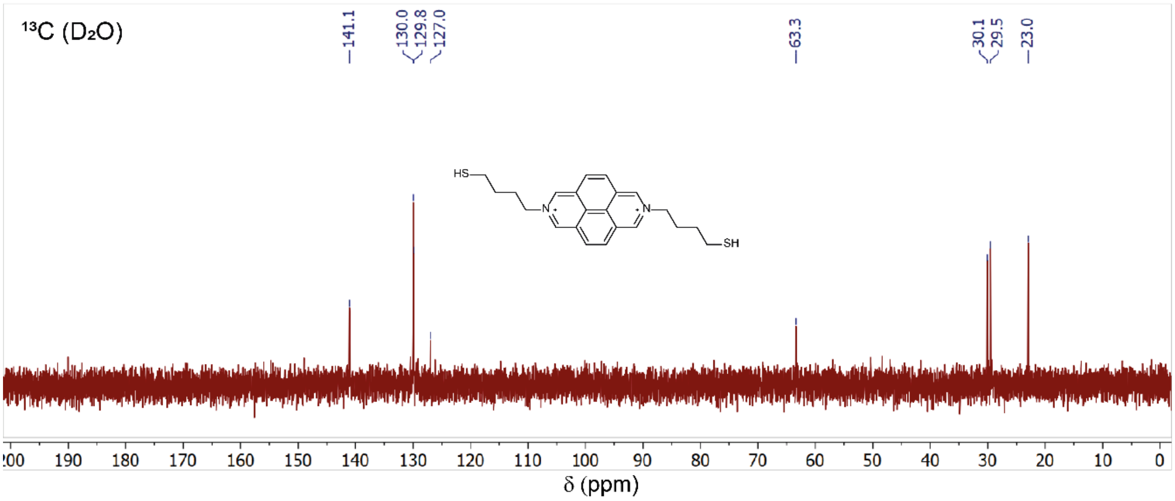

Supplementary Fig. 37. ^{13}C NMR spectrum (126 MHz) 2,7-bis(4-mercaptobutyl)benzo[*lmn*][3,8]phenanthroline-2,7-diium dibromide (**7**) in D_2O.

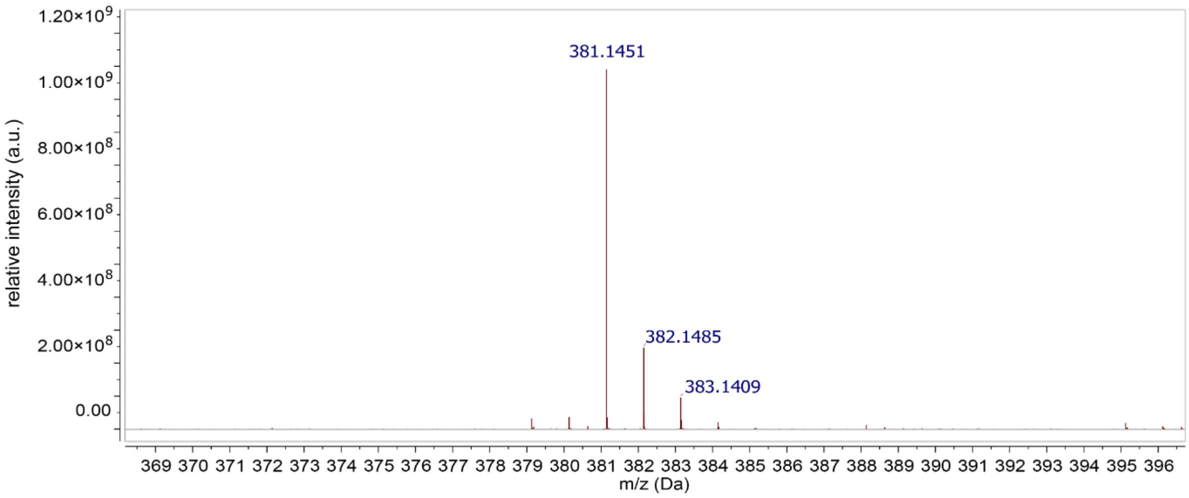

Supplementary Fig. 38. ESI MS spectra of 2,7-bis(4-mercaptobutyl)benzo[*lmn*][3,8]phenanthroline-2,7-diium dibromide (**7**).

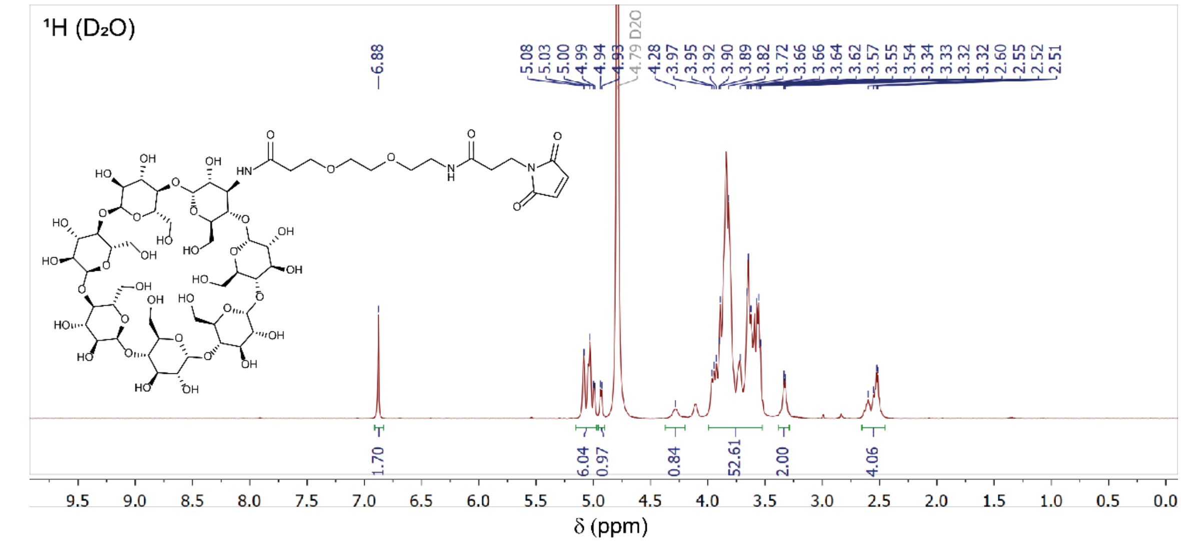

Supplementary Fig. 39. ^{1}H NMR spectrum (500 MHz) TEG-maleimide-β-CD (**8**) in D_2O.

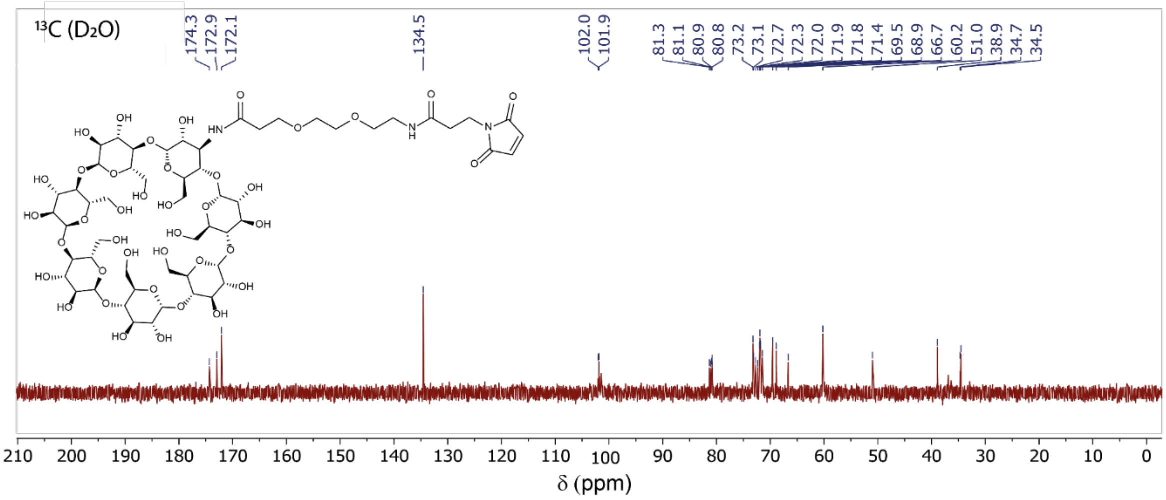

Supplementary Fig. 40. ^{13}C NMR spectrum (126 MHz) of TEG-maleimide-β-CD (**8**) in D_2O.

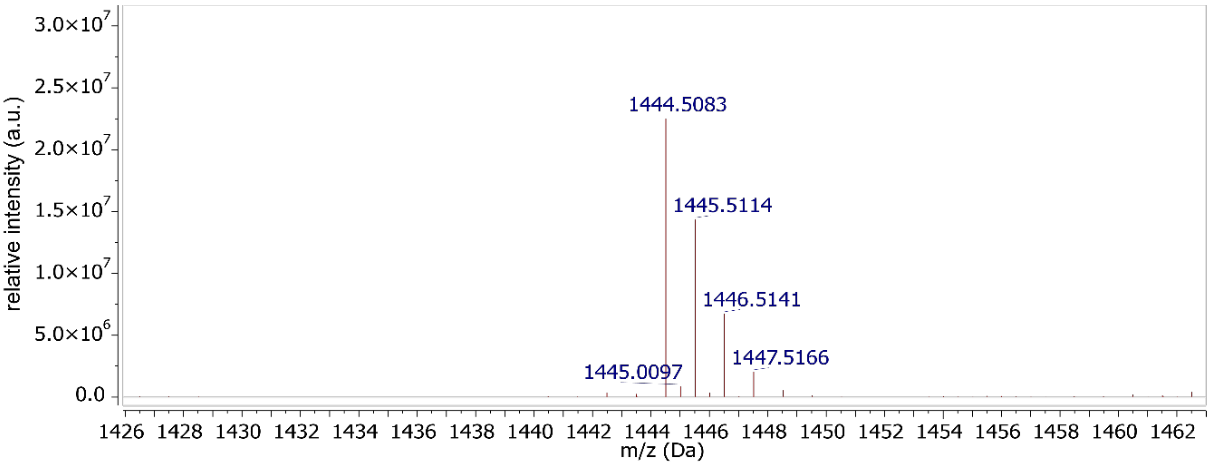

Supplementary Fig. 41. ESI MS spectra of TEG-maleimide-β-CD (**8**).

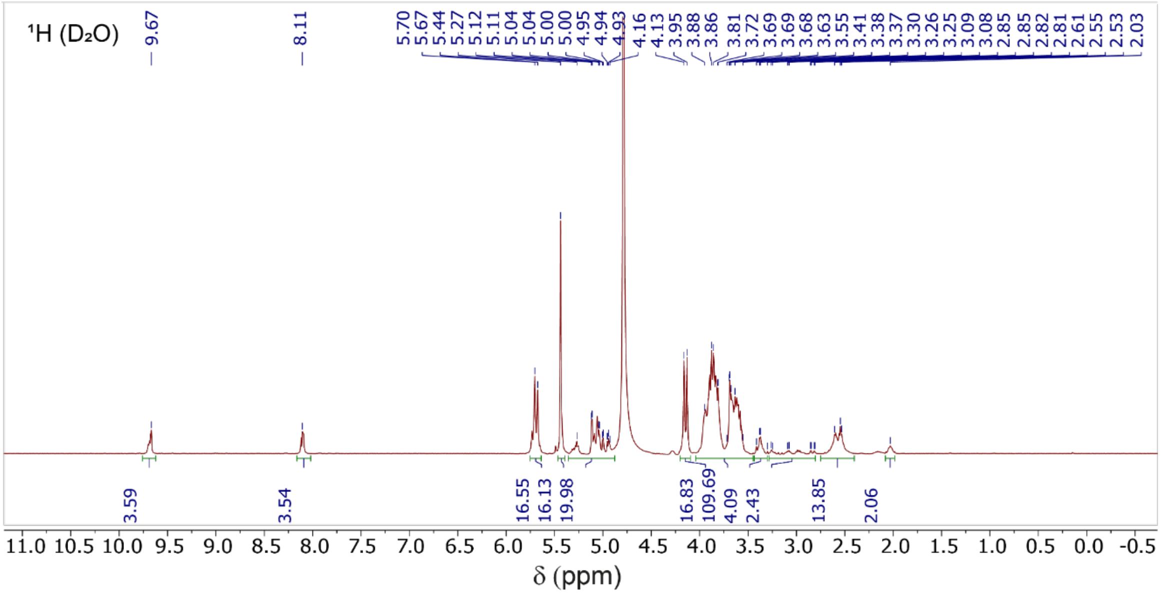

Supplementary Fig. 42. ^{1}H NMR spectrum (500 MHz) of rotaxane (**1**) in D_2O.

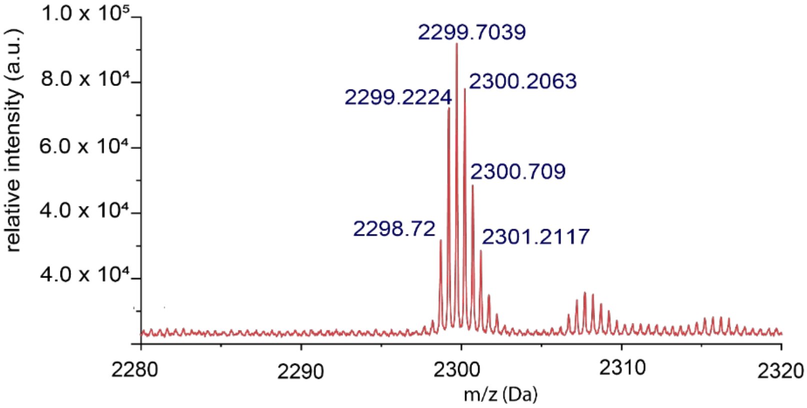

Supplementary Fig. 43. ESI MS spectra of rotaxane (**1**).

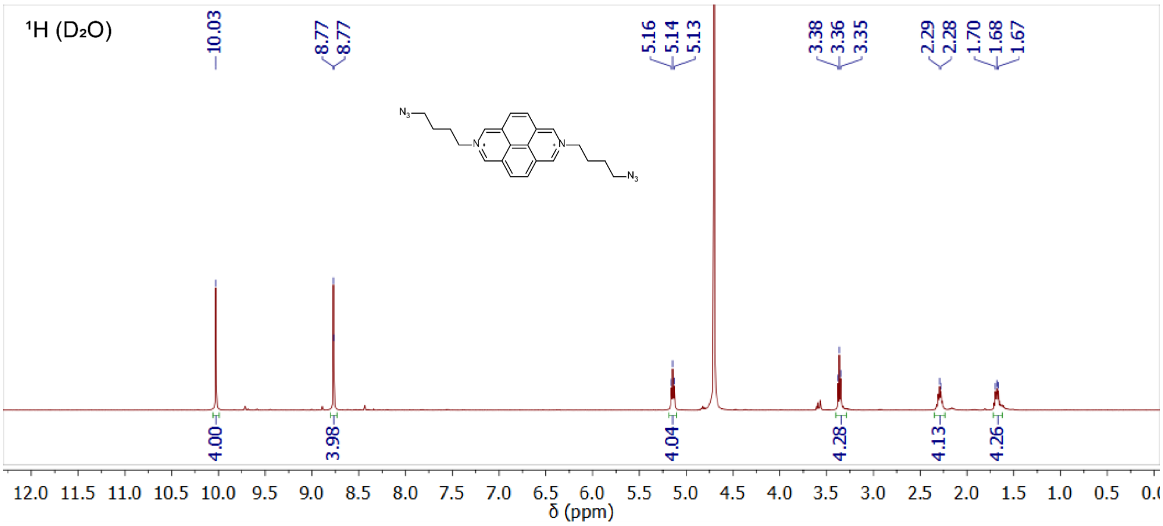

Supplementary Fig. 44. ^{1}H NMR spectrum (500 MHz) of 2,7-bis(4-azidobutyl)benzo[*lmn*][3,8]phenanthroline-2,7-diium dibromide (**9**) in D_2O.

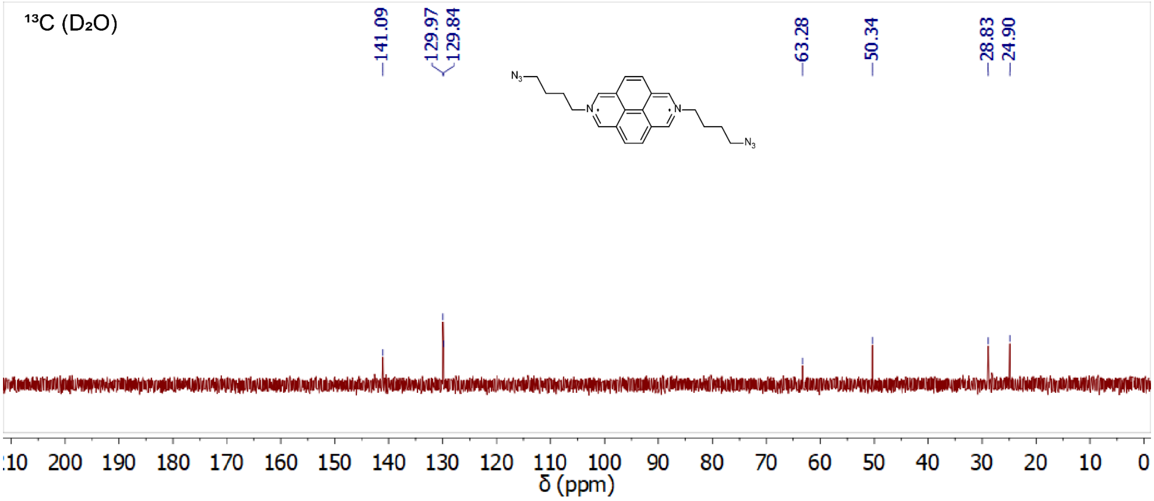

Supplementary Fig. 45. ^{13}C NMR spectrum (126 MHz) of 2,7-bis(4-azidobutyl)benzo[*lmn*][3,8]phenanthroline-2,7-diium dibromide (**9**) in D_2O.

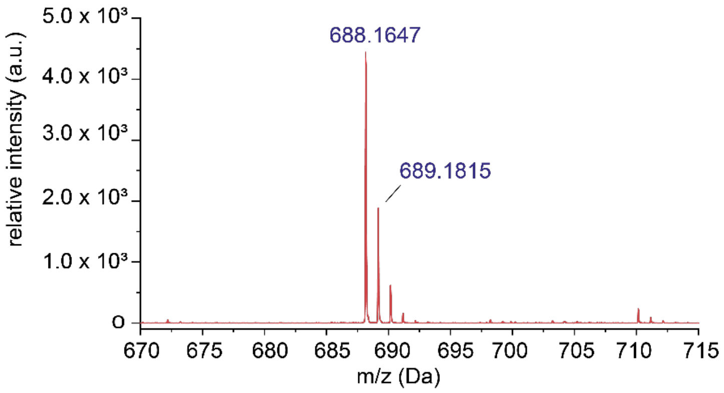

Supplementary Fig. 46. ESI MS spectra of DBCO-fluorescein (**10**)

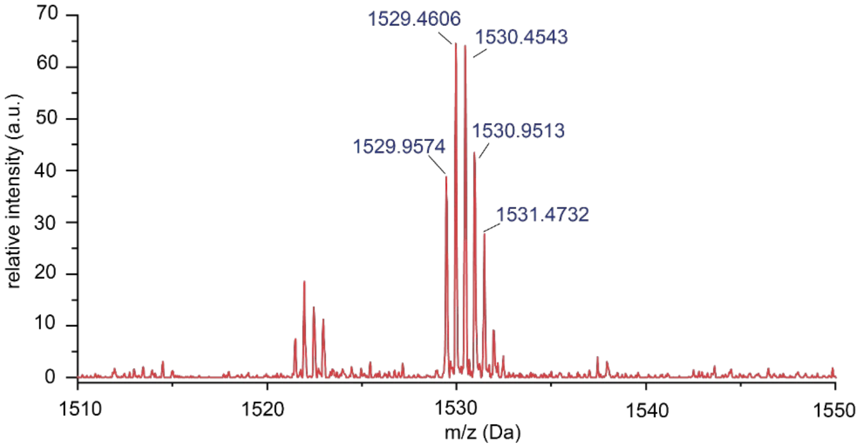

Supplementary Fig. 47. ESI MS spectra of DBCO-fluorescein rotaxane (**11**).

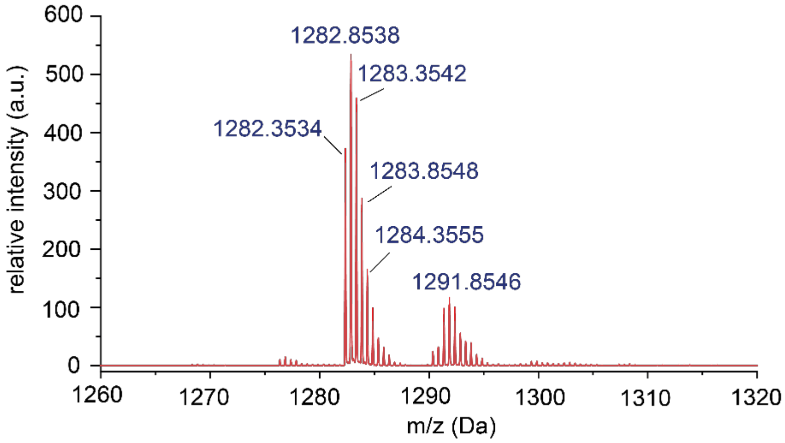

Supplementary Fig. 48. ESI MS spectra of FITC-rotaxane (**12**).

4. Abbreviations

ACN	Acetonitrile
AdOH	1-Adamantanol
Arg	Arginine
β-CD	beta-Cyclodextrin
BC	Berberine chloride
BSA	Bovine serum albumin
C	Concentration
CB7	Cucurbit[7]uril
CB8	Cucurbit[8]uril
Cps	Counts per seconds
CT	Charge transfer
γ	Gyromagnetic constant
D	Translational diffusion constant
D_2O	Deuterium oxide
Δ	Diffusion delay
δ	Chemical shift, gradient pulse length
DAP	2,7-Diazapyrene
DAPI	4',6-Diamidin-2-phenylindol
DBA	Direct binding assay
DBCO	Dibenzocyclooctyne
DBTDL	Dibutyltin dilaurate
DEPT	Distortionless enhancement by polarization transfer
DPT^{2+}	2,7-Dimethyl-diazaphenanthrenium
DOSY	Diffusion ordered spectroscopy
ESI	Electrospray ionization
ECD	Electronic circular dichroism
e.g.	Exempli gratia = 'for example'
ε	Extinction coefficient
Eq / Equiv	Equivalent
EQ	Emission quenching
G	Guest
GDA	Guest displacement assay
g	Gradient parameter or gram
HEPES	4-(2-Hydroxyethyl)-1-piperazineethane-sulfonic acid
HTS	High-throughput screening
HPLC	High-performance liquid chromatography
K_a	Binding constant
I_{em}	Emission intensity
I_0	Unattenuated signal intensity (DOSY)
IAA	Indole acetic acid
i.e.	Id est = 'that is'
λ	Wavelength
LC	Liquid chromatography
LOD	Limit of detection
Lys	Lysine
$MDAP^{2+}$	2,7-Dimethylpyrenium
MDI	4,4′-Diisocyanato methylendibenzol
$MDPP^{2+}$	2,9-Dimethyl peroperylenium
MS	Mass spectromety
MVE^{2+}	1,2-Dimethyl(4-pyridinyl)ethenium
m/z	Mass-to-charge ratio
µCS	Microchannel cantilever spotting
NMR	Nuclear magnetic resonance
PBS	Phosphate buffered saline
PEG	Polyethylene glycol
Phe	Phenylalanine
ref.	Reference
Sccm	Standard cubic centimetre per minute
sCx4	Sulfonated calix[4]arene
TCEP	Tris-(2-carboxyethyl)phosphine
TEG	Tetraethylene glycol
TFA	Trifluoroacetic acid
2,6-TNS	2-*p*-Toluidino-6-naphthalenesulfonic acid
Trp	Tryptophan
UV-Vis	Ultraviolet-visible
wt%	Mass fraction (weight percentage)

Supplementary Tables

Supplementary Table 1. Typical L-tryptophan concentration ranges for healthy and diseased adults in blood serum, blood plasma, and urine.

	[Trp] (μM)	medium	ref.
no disease	53 - 77	blood serum	14
	21 - 93	urine	15
Alzheimer's disease	24 - 42	blood serum	16
	15 - 22	urine	16
chronic kidney disease	17 - 26	blood serum	17
cardiovascular disease	27 - 76	blood serum	18
anaemia of inflammation	22 - 43	blood serum	19
coronary heart disease	44 - 63	blood plasma	20
sepsis	25 - 40	blood plasma	21

Supplementary Table 2. Additional examples for synthetic binders of Trp.

synthetic binder	optical signal	reported medium	log K_a	concentration range	ref.
pillar[5]arene-type receptor	emission	H_2O: DMF (1:1)	~ 5	μM range (0 - 240 μM)	22
binary CB8•porphyrin-tin(IV) complex	emission	saline buffer	~ 4.5	μM range (0 - 14 μM)	23
fluorescent CB6 analogue	emission	acetate buffer	~ 6.5	low μM range*	24
binary CB8•dye complexes	emission	saline buffer	~ 4.5	low μM range**	25
calix[4]pyrrole	absorbance	MeOH	n.a.	low μM range	26

* not selective for Trp. ** not selective for Trp, as Trp-containing peptides were mainly examined. n.a. =not available.

Supplementary Table 3. Binding affinities (log K_a) of rotaxane **1** for L-tryptophan in 1X PBS at different temperatures with an estimated error of log K_a = 0.2 for repetitive measurements.

temperature (°C)	log K_a
25	3.94
35	3.98
40	3.87

Supplementary Table 4. Typical concentration ranges of various tested analytes for healthy adults in blood serum, blood plasma, and urine. (Typical creatinine levels in human urine are 97 – 230 μmol kg^{-1} d^{-1}. [27])

analyte	[Trp] (μM/mM creatinine) in urine	ref.	[Trp] (μM) in serum	ref.
indole	[a]		[a]	
kynurenine	0.08 - 1.31	28	0.7 – 3.0	29
tryptamine	0.01 - 0.049	30	0.23	31
dopamine	0.2 - 07	28	0.0008 – 0.009 [b]	32

indole acetic acid	0.6 - 5.4	28	11.4 – 45.7	33
L-Phe	3.5 - 11.2	28	57.6 – 98.5	15
amantadine	c		c	
cadaverine	0.0513 – 0.0647	34	0.25 - 0.39	34
histamine	0.01 - 0.1	28	0.0005 – 0.0018 [a]	35
L-Lys	3.6 – 56.1	28	120.4 – 236.8	15
L-Arg	3.2 – 14.6	28	99 – 128.2	15

[a] Only found in human feces. [b] In human blood plasma. [c] Amantadine is a drug used to treat Parkinson's disease and therefore does not naturally occur in blood serum or urine.

Supplementary Table 5. Determined L-tryptophan concentrations of different blood serum samples using quantitative HPLC with an estimated error of 10% for repetitive measurements.

medium	[L-Trp] (µM) in serum
steroid-depleted human serum	< 1. ± 0.1
human serum 1	18.3 ± 0.2
human serum 2	21.9 ± 0.2
human serum 3	64.0 ± 0.6
bovine calf serum	38.2 ± 0.4

Supplementary Table 6. Determined HPLC recoveries of L-tryptophan concentrations in human blood serum for two different orders of sample treatment using quantitative HPLC with an estimated error of 10% for repetitive measurements.

sample treatment	[Trp] added (µM)	expected [Trp] (µM)	found (µM)	recoveries (%)
1) serum spiked with Trp	10	19.2	18.7	95.7
2) serum deproteinized	25	34.2	33.0	95.3
1) serum deproteinized	10	19.2	20.9	117
2) serum spiked with Trp	25	34.2	37.4	113

Supplementary Table 7. L-Tryptophan concentrations of different urine samples were determined using quantitative HPLC with an estimated error of 10% for repetitive measurements. Creatinine concentrations were determined using a commercially available colorimetric quantification kit.

medium	[L-Trp] (µM) in urine	[creatinine] (mg/dL) in urine
urine sample 1	7.2 ± 0.7	59
urine sample 2	24.3 ± 2.4	110

Supplementary References

1. Stang, P.J., Cao, D.H., Saito, S. & Arif, A.M. Self-assembly of cationic, tetranuclear, pt(ii) and pd(ii) macrocyclic squares. X-ray crystal structure of [pt2+(dppp)(4,4'-bipyridyl).Cntdot.2-oso2cf3]4. *J. Am. Chem. Soc.* **117**, 6273-6283 (1995).
2. Hünig, S., Groβ, J., Lier, E.F. & Quast, H. Über zweistufige redoxsysteme, xii1) synthese und polarographie von quartärsalzen der phenanthroline, des 2,7-diazapyrens sowie der diazoniapentaphene. *Liebigs Ann. Chem.* **1973**, 339-358 (1973).
3. Ellman, G.L. Tissue sulfhydryl groups. *Arch. Biochem. Biophys.* **82**, 70-77 (1959).
4. Verch, A., Hahn, H., Krause, E., Cölfen, H. & Börner, H.G. A modular approach towards functional decoration of peptide–polymer nanotapes. *Chem. Commun.* **46**, 8938-8940 (2010).
5. Loftsson, T., Saokham, P. & Sá Couto, A.R. Self-association of cyclodextrins and cyclodextrin complexes in aqueous solutions. *Int. J. Pharm.* **560**, 228-234 (2019).
6. Legg, K.D. & Hercules, D.M. Quenching of lucigenin fluorescence. *J. Phys. Chem.* **74**, 2114-2118 (1970).
7. Zhang, S., Grimm, L., Miskolczy, Z., Biczók, L., Biedermann, F. & Nau, W.M. Binding affinities of cucurbit[n]urils with cations. *Chem. Commun.* **55**, 14131-14134 (2019).
8. Liu, Y. & You, C.-C. Inclusion complexation of β-cyclodextrin and 6-o-α--maltosyl- and 2-o-(2-hydroxypropyl)-β-cyclodextrins -with some fluorescent dyes. *J. Phys. Org. Chem.* **14**, 11-16 (2001).
9. Sinn, S., Krämer, J. & Biedermann, F. Teaching old indicators even more tricks: Binding affinity measurements with the guest-displacement assay (gda). *Chem. Commun.* **56**, 6620-6623 (2020).
10. Shaw, A.K. & Pal, S.K. Resonance energy transfer and ligand binding studies on ph-induced folded states of human serum albumin. *J. Photochem. Photobiol. B: Biol.* **90**, 187-197 (2008).
11. Haque, N. & Prabhu, N.P. Insights into protein–tns (2-p-toluidinylnaphthalene-6-sulfonate) interaction using molecular dynamics simulation. *J. Mol. Struct.* **1068**, 261-269 (2014).
12. Lee, J.W., Samal, S., Selvapalam, N., Kim, H.-J. & Kim, K. Cucurbituril homologues and derivatives: New opportunities in supramolecular chemistry. *Acc. Chem. Res.* **36**, 621-630 (2003).
13. Zhong, C., Hu, C., Kumar, R., Trouillet, V., Biedermann, F. & Hirtz, M. Cucurbit[n]uril-immobilized sensor arrays for indicator-displacement assays of small bioactive metabolites. *ACS Appl. Nano Mater.* **4**, 4676-4687 (2021).
14. Wirleitner, B., *et al.* Immune activation and degradation of tryptophan in coronary heart disease. *Eur. J. Clin. Investig.* **33**, 550-554 (2003).
15. Psychogios, N., *et al.* The human serum metabolome. *PLoS One.* **6**, e16957 (2011).
16. Fonteh, A.N., Harrington, R.J., Tsai, A., Liao, P. & Harrington, M.G. Free amino acid and dipeptide changes in the body fluids from alzheimer's disease subjects. *Amino Acids.* **32**, 213-224 (2007).
17. Li, Y., Tang, A.-G. & Mu, S. Hplc–fld determination of serum aromatic amino acids: Application in chronic kidney disease patients. *Clin. Chim. Acta.* **412**, 1032-1035 (2011).
18. Konje, V.C., *et al.* Tryptophan levels associate with incident cardiovascular disease in chronic kidney disease. *Clin. Kidney J.* **14**, 1097-1105 (2020).
19. Weiss, G., Schroecksnadel, K., Mattle, V., Winkler, C., Konwalinka, G. & Fuchs, D. Possible role of cytokine-induced tryptophan degradation in anaemia of inflammation. *Eur. J. Haematol.* **72**, 130-134 (2004).
20. Herrera-Van Oostdam, A.S., *et al.* Immunometabolic signatures predict risk of progression to sepsis in covid-19. *PLoS One.* **16**, e0256784 (2021).
21. Ploder, M., *et al.* Accelerated tryptophan degradation predicts poor survival in trauma and sepsis patients. *Int. J. Tryptophan Res.* **3**, IJTR.S3983 (2010).
22. Zhang, Y.-M., *et al.* N-(2-aminoethyl)-2-(hexylthio) acetamide-functionalized pillar[5]arene for the selective detection of l-trp through guest-adaptive multisupramolecular interactions. *J. Phys. Chem. A.* **124**, 9811-9817 (2020).
23. Shee, N.K., Kim, M.K. & Kim, H.-J. Fluorescent chemosensing for aromatic compounds by a supramolecular complex composed of tin(iv) porphyrin, viologen, and cucurbit[8]uril. *Chem. Commun.* **55**, 10575-10578 (2019).
24. Lagona, J., Wagner, B.D. & Isaacs, L. Molecular-recognition properties of a water-soluble cucurbit[6]uril analogue. *J. Org. Chem.* **71**, 1181-1190 (2006).

25. Bush, M.E., Bouley, N.D. & Urbach, A.R. Charge-mediated recognition of n-terminal tryptophan in aqueous solution by a synthetic host. *J. Am. Chem. Soc.* **127**, 14511-14517 (2005).
26. Desai, A.L., *et al.* Calix[4]pyrrole based scrupulous probe for track on of tryptophan: Host-guest interaction, in silico modeling and molecular docking insights. *Chem. Phys.* **554**, 111426 (2022).
27. J. Krämer, L.M.G., R. Kang, L. De Cola, P. Picchetti, F. Biedermann Molecular probes, chemosensors, and nanosensors for optical detection of biorelevant molecules and ions in aqueous media and biofluids. *Chem. Rev.* **in press**, (2021).
28. Bouatra, S., *et al.* The human urine metabolome. *PLoS One.* **8**, e73076 (2013).
29. Hervé, C., Beyne, P., Jamault, H. & Delacoux, E. Determination of tryptophan and its kynurenine pathway metabolites in human serum by high-performance liquid chromatography with simultaneous ultraviolet and fluorimetric detection. *J. Chromatogr. B Biomed. Sci. Appl.* **675**, 157-161 (1996).
30. Magos, L. C. Lentner (ed.). Geigy scientific tables, 8th edition. Vol. 1. Units of measurement. Body fluids. Composition of the body. Nutrition. 1981, 298 pp. Vol. 2. Introduction to statistics. Statistical tables. Mathematical formulae. 1982, 241 pp. Vol. 3. Physical chemistry. Composition of the blood. Haematology. Human somatometric data. 1984, 359 pp. Vol. 4. Biochemistry. Metabolism of xenobiotics. Inborn error of metabolism. Pharmacogenetics and ecogenetics. 1986, 330 pp. Ciba-geigy, basel, £12.50 each volume. Distributed in u.K. By farrand press. *J. Appl. Toxicol.* **7**, 413-413 (1987).
31. Borg, J., Warter, J.M., Schlienger, J.L., Imler, M., Marescaux, C. & Mack, G. Neurotransmitter modifications in human cerebrospinal fluid and serum during hepatic encephalopathy. *J. Neurol. Sci.* **57**, 343-356 (1982).
32. Lambert, G., Naredi, S., Edén, E., Rydenhag, B. & Friberg, P. Monoamine metabolism and sympathetic nervous activation following subarachnoid haemorrhage: Influence of gender and hydrocephalus. *Brain Res Bull.* **58**, 77-82 (2002).
33. JOURDE-CHICHE, N., *et al.* Levels of circulating endothelial progenitor cells are related to uremic toxins and vascular injury in hemodialysis patients. *J. Thromb. Haemost.* **7**, 1576-1584 (2009).
34. Löser, C., Fölsch, U.R., Paprotny, C. & Creutzfeldt, W. Polyamine concentrations in pancreatic tissue, serum, and urine of patients with pancreatic cancer. *Pancreas.* **5**, (1990).
35. Gill, D.S., Fonseca, V.A., Barradas, M.A., Balliod, R., Moorhead, J.F. & Dandona, P. Plasma histamine in patients with chronic renal failure and nephrotic syndrome. *J Clin Pathol.* **44**, 243-245 (1991).
