# Supplementary material for: A supramolecular cucurbit[8]uril-based rotaxane chemosensor for the optical tryptophan detection in human serum and urine
Source: Nat Commun. 2023 Jan 31;14:518. doi: 10.1038/s41467-023-36057-3 (PMC9889744; doi:10.1038/s41467-023-36057-3)
Supplement: Supplementary file 2 — Reporting Summary [file 41467_2023_36057_MOESM2_ESM.pdf]

nature portfolio

Corresponding author(s): Dr. Frank Biedermann, Dr Michael Hirtz

Last updated by author(s): Dec 14, 2022

Reporting Summary

Nature Portfolio wishes to improve the reproducibility of the work that we publish. This form provides structure for consistency and transparency in reporting. For further information on Nature Portfolio policies, see our Editorial Policies and the Editorial Policy Checklist.

Statistics

For all statistical analyses, confirm that the following items are present in the figure legend, table legend, main text, or Methods section.

n/a	Confirmed	
☐	☒	The exact sample size (*n*) for each experimental group/condition, given as a discrete number and unit of measurement
☐	☒	A statement on whether measurements were taken from distinct samples or whether the same sample was measured repeatedly
☒	☐	The statistical test(s) used AND whether they are one- or two-sided<br>*Only common tests should be described solely by name; describe more complex techniques in the Methods section.*
☒	☐	A description of all covariates tested
☒	☐	A description of any assumptions or corrections, such as tests of normality and adjustment for multiple comparisons
☐	☒	A full description of the statistical parameters including central tendency (e.g. means) or other basic estimates (e.g. regression coefficient) AND variation (e.g. standard deviation) or associated estimates of uncertainty (e.g. confidence intervals)
☒	☐	For null hypothesis testing, the test statistic (e.g. *F*, *t*, *r*) with confidence intervals, effect sizes, degrees of freedom and *P* value noted<br>*Give P values as exact values whenever suitable.*
☒	☐	For Bayesian analysis, information on the choice of priors and Markov chain Monte Carlo settings
☒	☐	For hierarchical and complex designs, identification of the appropriate level for tests and full reporting of outcomes
☒	☐	Estimates of effect sizes (e.g. Cohen's *d*, Pearson's *r*), indicating how they were calculated

Our web collection on statistics for biologists contains articles on many of the points above.

Software and code

Policy information about availability of computer code

Data collection	Data was obtained on a fluorimeter, CD, and UV/Vis spectrometer from JASCO with Spectra Manager Version 2, on a microplate reader from Perkin Elmer as well as BMG Labtech, running Kaleido 3.0 and MARS 4.01 R2 as softwares, on a HPLC from JASCO using ChromNav Version 2.0 software, DLS measurements were performed on a Malvern Zetasizer Nano ZS instrument running Nanosoftware V3.30 and fluorecence imaging of printed microarrays were performed on an inverted fluorescence microscope NIKON Eclipse Ti2 using NIKON's proprietary software NIS-Elements.
Data analysis	Fitting routines implemented in Origin 9.8 were used, all binding constants were determined according to a 1:1 fit of the obtained data points with an R^2 >/= 0.98 for each fit. Binding constants were averaged over at least 3 measurements and an error of log Ka = 0.2 was generously estimated to include errors such as pipetting errors. Emission quenching in serum samples were determined using the described formula and calculations were done in Excel (Version 2209) and Origin 9.8. HPLC-based quantifications were done by integrating the absolute peak area or height of each fluorescence signal using the ChromNav software (Version 2.0) provided by JASCO and an error of 5% was estimated for each mean. Analysis of the microarrays was performed using NIS-Elements giving the mean of at least 3 repetitions and their standard deviation. NMR and ESI MS data were analyzed using MNova 14.2, DOSY NMR were analyzed using TopSpin3.1. Bargraphs and spectra were plotted in Origin 9.8, Chemical structures were drawn using Chemdraw 19.1.1.21.

For manuscripts utilizing custom algorithms or software that are central to the research but not yet described in published literature, software must be made available to editors and reviewers. We strongly encourage code deposition in a community repository (e.g. GitHub). See the Nature Portfolio guidelines for submitting code & software for further information.

Data

Policy information about availability of data

All manuscripts must include a data availability statement. This statement should provide the following information, where applicable:

- Accession codes, unique identifiers, or web links for publicly available datasets
- A description of any restrictions on data availability
- For clinical datasets or third party data, please ensure that the statement adheres to our policy

Source data are provided with this paper. All raw and processed data that support the findings of this study are available on Zenodo.org with the identifier (DOI: 10.5281/zenodo.7434298). Furthermore, binding parameters and chemical structures of the chemosensors of this study are available on suprabank.org with the identifier (DOI: 10.34804/supra.20220128415). Synthetic procedures and analysis data that support the findings of this study can be found in the Supplementary Information and are readily available on chemotion-repository.net with the following links for the individual compounds:
https://dx.doi.org/10.14272/reaction/SA-FUHFF-UHFFFADPSC-FERUHHWVLZ-UHFFFADPSC-NUHFF-NUHFF-NUHFF-ZZZ.1
https://dx.doi.org/10.14272/reaction/SA-FUHFF-UHFFFADPSC-VHNSKBKZGH-UHFFFADPSC-NUHFF-NUHFF-NUHFF-ZZZ
https://dx.doi.org/10.14272/reaction/SA-FUHFF-UHFFFADPSC-ZPIPUFJBRZ-UHFFFADPSC-NUHFF-NUHFF-NUHFF-ZZZ.1
https://dx.doi.org/10.14272/reaction/SA-FUHFF-UHFFFADPSC-LHBYXQPXZL-UHFFFADPSC-NUHFF-LUHFF-NUHFF-ZZZ
https://dx.doi.org/10.14272/reaction/SA-FUHFF-UHFFFADPSC-LNNFMCALYY-UHFFFADPSC-NUHFF-LUHFF-NUHFF-ZZZ
https://dx.doi.org/10.14272/reaction/SA-FUHFF-UHFFFADPSC-DYWOOYJZBS-UHFFFADPSC-NUHFF-NUHFF-NUHFF-ZZZ
https://dx.doi.org/10.14272/reaction/SA-FUHFF-UHFFFADPSC-UFNDHZJPQR-UHFFFADPSC-NUHFF-NUOFM-NUHFF-ZZZ
https://dx.doi.org/10.14272/reaction/SA-FUHFF-UHFFFADPSC-YRODUCXAHM-UHFFFADPSC-NUHFF-NDLGW-NUHFF-ZZZ

Human research participants

Policy information about studies involving human research participants and Sex and Gender in Research.

Reporting on sex and gender	Pooled human serum samples were purchased from Merck, Biowest, and Seqens. The suppliers did not give information on the sex or gender of the sample donors. For the human urine samples, neither sex nor gender data were collected in this study since urine only served as a biomatrix for a proof-of-principle and was exclusively used in spiking experiments. Collected data were not used to investigate diseases, and therefore reporting sex and gender was not relevant to this study.
Population characteristics	Population characteristics are not reported in the manuscript as the used serum and urine were exclusively utilized as a medium for spiking with Trp to show the functionality of the designed system, and therefore population characteristics are not relevant.
Recruitment	Pooled human serum samples were purchased from Merck, Biowest, and Seqens. For the human urine samples, the participation of interested healthy individuals was voluntary after detailed information about the study was given. Informed consent was obtained from all participants. Urine samples were collected anonymously, placing the urine sample into a numbered container (blinded) at a defined time. A total number of five urine samples was collected of which three samples were chosen randomly for investigations. The filled containers were taken from the deposit storage place in the fridge and used for HPLC analysis and plate reader assays within that day. No compensation was provided to the participants.
Ethics oversight	The guidelines of the KIT Ethics Commission were followed.<br>All procedures performed in this studies were in accordance with the formal statement of ethical principles published by the World Medical Association in the Declaration of Helsinki in 1964 and its later amendments or comparable ethical standards.

Note that full information on the approval of the study protocol must also be provided in the manuscript.

Field-specific reporting

Please select the one below that is the best fit for your research. If you are not sure, read the appropriate sections before making your selection.

☒ Life sciences ☐ Behavioural & social sciences ☐ Ecological, evolutionary & environmental sciences

For a reference copy of the document with all sections, see nature.com/documents/nr-reporting-summary-flat.pdf

Life sciences study design

All studies must disclose on these points even when the disclosure is negative.

Sample size	No statistical methods for sample size predetermination were used for any measurements. The sample size of binding affinities (with n = 3 independent replicates and a minimum of 30 data points) was selected after careful evaluation of former binding studies conducted in our group, which gave good R2 values (> 0.98) for the fits of the obtained data. For the measurements using serum samples, a sample size of five samples from three different suppliers was selected to get a wide variety of serum samples from different origins. For the urine measurements, a sample size of three was chosen to show the system's functionality in urine as a complex biomedia (two samples used in microwell plate reader-based measurements and one sample used in printing experiments). For plate reader-based measurements, single-point measurements were conducted at a fixed wavelength with one data point for each well.
Data exclusions	No data points were excluded for fitting fluorescence-based titration binding affinities and microarray analysis. For plate reader-based measurements of the calibration curve in deproteinized serum and HPLC-based serum measurements, a maximum of four data points was excluded for analysis. For platereader-based measurements in urine, a maximum of two data points were excluded. The exclusion was caused by the examination of big outliers which can be related to bubbles in microplate wells due to premeasurement shaking in the plate reader as well as titration errors (i.e., air bubbles in pipette tip and, therefore, different solution volume). For HPLC-based measurements, errors occurred overnight in the automated sample loading, e.g., when the sample vial ran dry. These data were therefore excluded from the analysis.
Replication	Titration Experiments were repeated at least three times and were successful. The platereader-based calibration curve of deproteinized serum was measured in 12 replicates. However, due to titration errors, 4 repetitions were unsuccessful and excluded from the data analysis. For reader-based measurements, each serum sample was measured in eight/six/five repetitions (exact n values are stated for each experiment in the Figure caption and method section), and all repetitions were successful. For plate reader-based measurements, each urine sample was measured in six repetitions of which only four replicates were successful. For the screening of quenching efficiencies of different analytes in 1X PBS four repetitions were conducted for each analyte concentration and were successful. HPLC-based quantification of serum samples was conducted in at least three replicates and was apart from technical errors successful. Analyte detection using fluorescence imaging of printed rotaxane microarrays was performed in at least three measurements and was successful. All concentration-dependent rotaxane microarray printing measurements were done once per concentration or dilution and were successful. However, several concentrations/dilutions (e.g., 5) were measured within each experiment. Also, several plates (e.g., 5) were printed for the experiments, as each concentration/dilution measurement had to be performed on a new plate. Therefore, we consider these measurements as independent replicates which prove the prints' reproducibility.
Randomization	Randomization was used to select three urine samples out of five, by pre-selecting numbers between 1 and 5 to pick a urine sample using dice.
Blinding	Doubly blinded procedures were used for all tested urine samples. For all other experiments, the blinding was not relevant.

Reporting for specific materials, systems and methods

We require information from authors about some types of materials, experimental systems and methods used in many studies. Here, indicate whether each material, system or method listed is relevant to your study. If you are not sure if a list item applies to your research, read the appropriate section before selecting a response.

Materials & experimental systems

n/a	Involved in the study	
☒	☐	Antibodies
☒	☐	Eukaryotic cell lines
☒	☐	Palaeontology and archaeology
☒	☐	Animals and other organisms
☒	☐	Clinical data
☒	☐	Dual use research of concern

Methods

n/a	Involved in the study	
☒	☐	ChIP-seq
☒	☐	Flow cytometry
☒	☐	MRI-based neuroimaging
